# A novel scaffold for EGFR inhibition: Introducing *N*-(3-(3-phenylureido)quinoxalin-6-yl) acrylamide derivatives

**DOI:** 10.1038/s41598-018-36846-7

**Published:** 2019-01-09

**Authors:** Daniel Nascimento do Amaral, Jonas Lategahn, Harold Hilarion Fokoue, Eduardo Miguez Bastos da Silva, Carlos Mauricio R. Sant’Anna, Daniel Rauh, Eliezer J. Barreiro, Stefan Laufer, Lidia Moreira Lima

**Affiliations:** 1 Instituto Nacional de Ciência e Tecnologia de Fármacos e Medicamentos (INCT-INOFAR; http://www.inct-inofar.ccs.ufrj.br/), Laboratório de Avaliação e Síntese de Substâncias Bioativas (LASSBio®, http://www.lassbio.icb.ufrj.br) Universidade Federal do Rio de Janeiro, CCS, Cidade Universitária, Rio de Janeiro, RJ Brazil; 2 Programa de Pós-Graduação em Farmacologia e Química Medicinal, Instituto de Ciências Biomédicas, Universidade Federal do Rio de Janeiro, Avenida Carlos Chagas Filho, 373, Ilha do Fundão, 21941-912 Rio de Janeiro, RJ Brazil; 3 Department of Pharmaceutical/Medicinal Chemistry, Institute of Pharmacy and Interfaculty Center for Pharmacogenomics and Drug Research (ICEPHA), Eberhard-Karls-University Tübingen, Auf der Morgenstelle 8, 72076 Tübingen, Germany; 4 Faculty of Chemistry and Chemical Biology, TU Dortmund University, Otto-Hahn-Straße 4a, D-44227 Dortmund, Germany; 5 Instituto de Macromoléculas (IMA), Universidade Federal do Rio de Janeiro, Rio de Janeiro, RJ Brazil; 6 Departamento de Química, Universidade Federal Rural do Rio de Janeiro, Seropédica, RJ Brazil

## Abstract

Clinical data acquired over the last decade on non-small cell lung cancer (NSCLC) treatment with small molecular weight Epidermal Growth Factor Receptor (EGFR) inhibitors have shown significant influence of EGFR point mutations and in-frame deletions on clinical efficacy. Identification of small molecules capable of inhibiting the clinically relevant EGFR mutant forms is desirable, and novel chemical scaffolds might provide knowledge regarding selectivity among EGFR forms and shed light on new strategies to overcome current clinical limitations. Design, synthesis, docking studies and *in vitro* evaluation of *N*-(3-(3-phenylureido)quinoxalin-6-yl) acrylamide derivatives (7a-m) against EGFR mutant forms are described. Compounds 7h and 7l were biochemically active in the nanomolar range against EGFR_wt_ and EGFR_L858R_. Molecular docking and reaction enthalpy calculations have shown the influence of the combination of reversible and covalent binding modes with EGFR on the inhibitory activity. The inhibitory profile of 7h against a panel of patient-derived tumor cell lines was established, demonstrating selective growth inhibition of EGFR related cells at 10 μM among a panel of 30 cell lines derived from colon, melanoma, breast, bladder, kidney, prostate, pancreas and ovary tumors.

## Introduction

**Figure 1:**
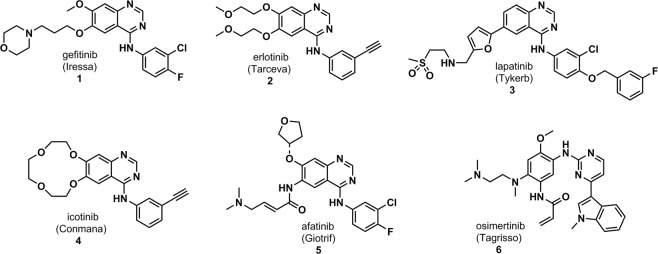
EGFR inhibitor drugs.

Over the last years there has been a growing interest for the identification of protein kinase inhibitors to treat several disorders^1,2^. Tyrosine kinase inhibitors (TKIs) drug discovery projects have emerged as an attractive field once approved drugs have been considered as efficient pharmacological tools mainly in cancer treatment^3,4^. To date, more than 35 small molecular weight protein kinase inhibitors have been approved for clinical use targeting a small number of protein kinases^5,6^. Protein kinases are druggable targets and their inhibition induces apoptosis in tumor cell lines presenting the oncogenic addiction phenomenon^7,8^. This feature assures the protein kinase inhibitors’ drugs-selective cytotoxicity to tumor cells overexpressing the targeted protein and sparing non-tumor cells. Therefore, targeted cancer therapy is more tolerable to adverse effects than classic chemotherapy^9^. Yet, common adverse effects observed in targeted therapy are related to “on-target” inhibition of wild type EGFR^10,11^. Molecular modifications on chemical structures might dramatically change not only the potency of TKIs over their on-target^12–14^, but also affect their selectivity towards other non-related protein kinase (off-targets)^15,16^. This effect can be clearly observed in Epidermal Growth Factor Receptor inhibitors (EGFRi) drugs gefitinib (1), erlotinib (2), lapatinib (3), icotinib (4) afatinib (5), osimertinib (6) (Fig. 1).

Among the kinome, the Epidermal Growth Factor Receptor (EGFR) is one of the most studied protein kinases and its relationship with cancer has been known since the 1980’s^17^. EGFR is a receptor tyrosine kinase, a member of ErbB family together with HER-2, HER-3 and HER-4, related to cellular proliferation, cellular differentiation and cell survival^18,19^. As a receptor, EGFR has an extracellular domain, a transmembrane segment and an intracellular kinase domain^20^. Ligands such as EGF, TGF-alpha, epiregulin and others, bind to the extracellular domain. The EGFR activation process is initiated by homo- or heterodimerization with ErbB family members and corresponding signaling pathways, mainly PI3K/AKT and MAPK, are triggered. Deregulated activity of EGFR is related to aggressive tumors with poor prognosis^21,22^.

Clinical data acquired over the last decade, especially on non-small cell lung cancer (NSCLC) treatment with small molecular weight EGFR inhibitors, have shown significant influence of EGFR point mutations and in-frame deletions on clinical efficacy^23,24^. Activating EGFR point mutation L858R and exon19 in-frame deletion mutations are related to clinical response to erlotinib, gefitinib and afatinib^25,26^. These mutations comprise more than 80% of driver mutations found in EGFR driven NSCLC^27,28^. After one year of treatment, initially responsive patients showed a resistance to erlotinib or gefitinib-based tumor treatment associated to a secondary point mutation at the gatekeeper residue T790M^29,30^. Kinetic studies showed ATP and reversible EGFR inhibitors to have different affinities for EGFR wild-type and mutant forms^31^. ATP has a higher affinity to its catalytic binding site in EGFR-wt and EGFR harboring T790M mutation and, therefore, reversible inhibitors show lower potency, shorter residence time and lack of clinical efficacy in these forms. On the other hand, ATP has a lower affinity to EGFR L858R, and first generation EGFRi beat ATP with regard to its binding pocket, showing longer residence time and better clinical response.

The discovery of new TKI capable of inhibiting the clinically relevant EGFR mutant forms is desirable, and novel chemical scaffolds might provide knowledge regarding selectivity among EGFR forms and shed light on new strategies to overcome current clinical limitations. In this context, this work describes the design, synthesis and *in vitro* evaluation of new acrylamide-quinoxaline derivatives as a novel scaffold for EGFR inhibition.

## Results and Discussion

### Molecular design of quinoxaline EGFR inhibitors

**Figure 2:**
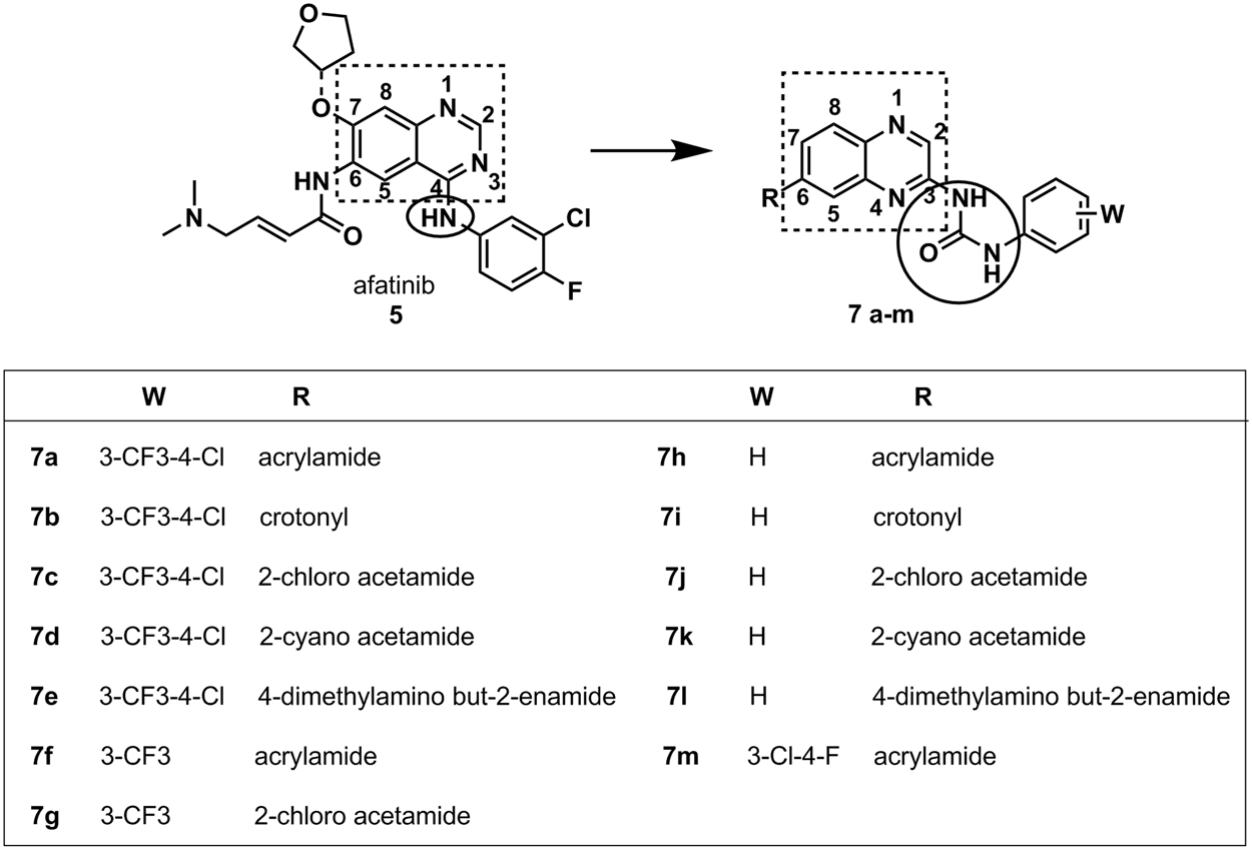
Molecular conception of quinoxaline urea derivatives **7a-m** designed as EGFR covalent inhibitors.

The molecular design conception was based on the bioisosteric replacement of the quinazoline aromatic ring by a quinoxaline scaffold^32^, maintaining sp^2^ nitrogen atoms for hydrogen bond interactions to the hinge region^33^. Subsequently, the aniline moiety was replaced by a urea subunit. Aiming to explore an eventual covalent interaction with EGFR cysteine 797 residue^34^, different electrophilic subunits were introduced to the position analogous to afatinib (**4**), allowing the design of compounds **7a-m** (Fig. 2). The election of the covalent reactive groups was based on previous works describing EGFR inhibition towards reversible and irreversible covalent bond with cysteine residues^35–38^. Additionally, chemical reactivity studies and promiscuity profiles of the covalent reactive groups were also considered^39,40^.

### Chemistry

**Figure 3:**
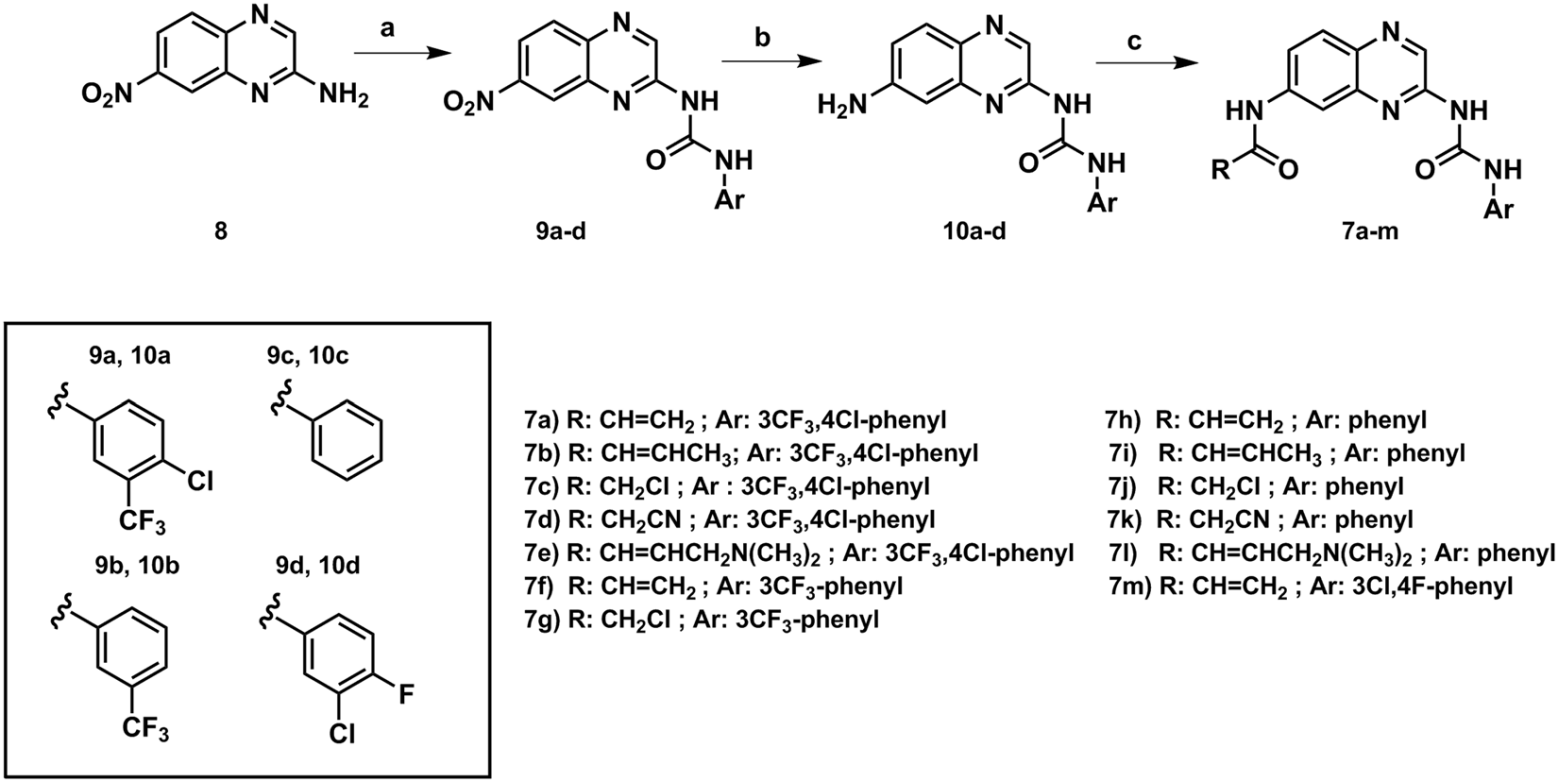
Reagents and conditions: (**a**) substituted phenyl isocyanates, dry toluene, reflux, 2–4 h, 58–82%; (**b**) SnCl_2_.2H_2_O, ethanol, reflux, overnight, 37–91%; (**c**) acyl chlorides, DIPEA, dry THF, 0 °C, 2–3 h, 12–70%.

Synthesis of the derivatives **7a-m** was performed through the synthetic methodology depicted in Fig. 3, employing 7-nitroquinoxaline-2-amine (**8**) as key intermediate. A simple multi-gram procedure to obtain 8 was developed, using the non-expensive and readily available *o*-phenylendiamine as starting material^41^. Substituted phenylureas (**9a-d**) were obtained by the reaction of a 7-nitroquinoxinoxaline-2-amine (**8**) derivative with isocyanates in dry toluene under reflux in moderate yields (58–82%)^42^. The nitro group was reduced to the corresponding aniline (**10a-d**) using tin (II) chloride dihydrate under reflux in ethanol with 37–91% yields^43^. In the final step, the anilines **10a-d** reacted with the previously selected acyl chlorides in dry THF in the presence of DIPEA as organic base to furnish the desired compounds **7a-m** after flash chromatography purification step^44^. Crotonoyl chloride, 2-cyanoacetyl chloride and (*E*)-4-(dimethylamino) but-2-enoyl chloride were prepared using their corresponding commercial available carboxylic acids in a procedure previous to the acylation reaction using oxalyl chloride in dichloromethane and catalytic DMF^45^.

### EGFR wt and mutant forms inhibition

EGFR-wt and mutant forms biochemical inhibitory activities for **7a-m** are depicted in Table 1. Enzymatic *in vitro* determination showed that *para*-substituted compounds **7a-e** and **7m** have weak or no inhibitory activity on EGFR forms. Moreover, *meta*-trifluoromethyl-substituted derivatives (**7f, 7g**) showed weak activity against EGFR_**wt**_ and EGFR_L858R_ and no inhibitory activity against EGFR_L858R/T790M_. These results indicate that substitutions at the phenyl ring of the urea moiety are deleterious to EGFR inhibitory activity. Biochemical data also suggest that the replacement of the aniline quinazoline scaffold, common in the structure of well-known EGFR inhibitors (e.g. afatinib, gefitinib), with quinoxaline phenylurea results in compounds sensitive to steric constraints at the binding site, probably associated with the augmentation of the distance between the heteroaromatic ring’s nitrogen, which is able to interact with the hinge region, and the urea phenyl ring. However, non-substituted phenylurea derivatives **7h** and **7l**, bearing as covalent reactive groups the acrylamide and 4-dimethylamino but-2-enamide moieties, respectively, displayed notorious EGFR inhibitory activity in the low nanomolar range. Compound **7h**, was identified as the most potent inhibitor of EGFR_**wt**_ (IC_**50**_ = 25 ± 4 nM) and EGFR_**L858R**_ (IC_**50**_ = 18 ± 3 nM). Despite that, weak inhibition of EGFR_L858R/T790M_ was observed. Compound **7l** was able to inhibit all EGFR forms (IC_**50**_ = 101 ± 12 nM for EGFR_**wt**_, IC_**50**_ = 32 ± 14 nM for EGFR_**L858R**_ and IC_**50**_ = 132 ± 49 nM for EGFR_**L858R/T790M**_) in 2–3 digits nanomolar range. As the covalent inhibition process relies on two steps^46^, an initial reversible ligand-receptor interaction followed by a covalent reaction of a nucleophile (EGFR C797 residue) with an electrophile (covalent reactive group), potency differences for EGFR inhibition observed between compounds **7h-l** (phenyl substituted) are expected. The presence of the tertiary amine group at the covalent reactive group can not only provide a better solubility profile, but can also act as an “*in situ*” catalyst during the covalent bond formation with cysteine residue^35,39^. Therefore, it might be responsible for the apparent potency in EGFR inhibition, including EGFR_**L858R/T790M**_, exhibited by compound **7l**. Compounds harboring 2-chloro acetamide as covalent reactive group were able to inhibit EGFR forms, even when the urea moiety was substituted with 3-CF_3_ and 4-Cl groups (compounds **7c, 7g**). This data reinforces that compounds with highly reactive warheads might inhibit protein kinase activity in a non-specific manner, making this warhead an improper subunit to design selective TKIs.

**Table 1:** EGFR (EGFR_wt_, EGFR_L858R_ and EGFR_L858R/T790M_) IC_50_ values (nM ± S.D./n ≥ 3) determined for compounds **7a-m**.

IC_50_ (nM) ± S.D.			
compounds	EGFR_**wt**_	EGFR_**L858R**_	EGFR_**L858R/T790M**_
gefitinib	<1	<1	185 ± 98
afatinib	<1	<1	<1
osimertinib	1 ± 0.6	<1	<1
**(7a)**	>10000	>10000	>10000
**(7b)**	>10000	>10000	>10000
**(7c)**	>10000	3237 ± 1257	2710 ± 412
**(7d)**	>10000	>10000	9867 ± 265
**(7e)**	>10000	>10000	6139 ± 3469
**(7f)**	2499 ± 735	2163 ± 1623	>10000
**(7g)**	179 ± 61	2099 ± 505	9829 ± 297
**(7h)**	25 ± 4	18 ± 3	1682 ± 1506
**(7i)**	>10000	5577 ± 4163	>10000
**(7j)**	123 ± 134	270 ± 202	823 ± 613
**(7k)**	3117 ± 787	445 ± 105	6293 ± 2789
**(7l)**	101 ± 12	32 ± 14	132 ± 49
**(7m)**	>10000	>10000	>10000

According to our data, compound **7h**, which has the simplest structure in the series, showed the highest inhibitory activity on EGFR_**wt**_ and EGFR_**L858R**_ forms. In comparison to the data obtained for compounds **7a**, **7f** and **7m**, these data endorse the deleterious contribution of substituents at the phenylurea moiety; and point out that further molecular modifications are needed for more efficient EGFR mutant forms’ inhibitors’ identification.

### Molecular docking

The experimental data are indicative that the presence of a *meta* or *para* substituent at the phenyl group was deleterious for the EGFR inhibition, so attempts to elucidate the binding mode with the enzyme were only implemented with the non-substituted compounds **7h-7l**, by means of molecular docking with GOLD 5.4 in the afatinib-containing wt-EGFR structure (PDB code: 4G5J).

Compounds **7h**, **7i** and **7l** have Michael acceptor groups, whereas compounds **7j** and **7k** have chloride and cyanide at the α-carbon to the carbonyl, respectively, which can act as leaving groups, so that a covalent bond can be possibly formed with the Cys797A sulfur atom by all compounds.

Initially, simple and covalent docking of the three Micheal acceptor inhibitors were performed to identify possible binding modes that could help in the explanation of the loss of activity of compound **7i** compared to the two other compounds.

The ChemPLP fitness function presented the best performance both in simple (RMSD equal to 2.81 Å) and covalent redocking studies (2.50 Å) based on the 4G5J [51] crystallographic structure. Simple docking studies confirmed the hypothesis that covalent ligands firstly form noncovalent adducts in the ATP binding site before the covalent bond is formed.

It was observed that all compounds have the same binding mode before the covalent bond is formed (Figs S1 and S2, supplementary material).

Covalent docking studies were performed at the electrophilic α-carbon of the carbonyl subunit (compounds **7j** and **7k**) and at the β-carbon of the enone subunit (**7h**, **7i** and **7l**).

**Figure 4:**
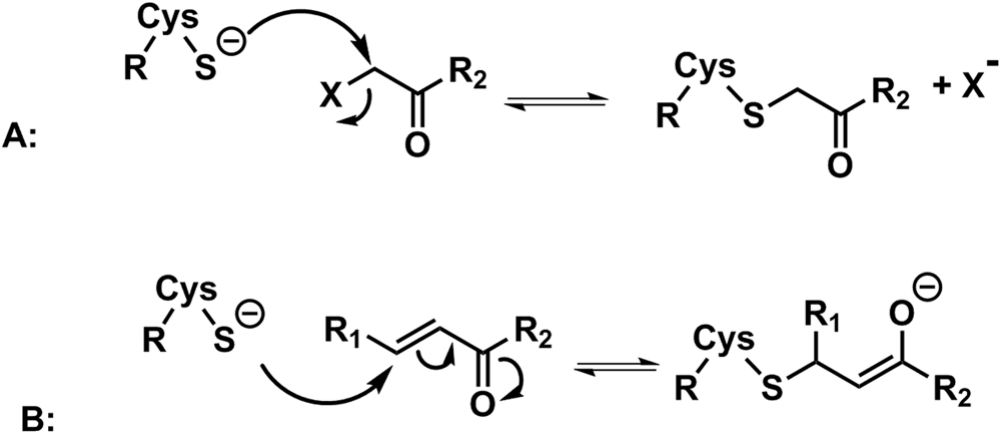
Cysteine (Cys797) residue attack scheme at the electrophilic carbon of the α-carbon of carbonyl subunit (**A**) and the enone subunit (**B)** of the quinoxaline urea derivatives.

Although molecular docking programs are effective in producing ligand-enzyme interaction geometries, the respective scores do not match the experimental activity data so well. For this reason, for compounds **7j** and **7k** the generated enzyme-inhibitor complexes (Fig. S3, supplementary material) were then used as input geometries for the calculation with the semi-empirical method PM7 [50] of the reaction enthalpies, which play a significant role in the enzyme-inhibitor complex stability. The results were analyzed from the point of view of the relative reaction enthalpies for the formation of a ligand-enzyme adduct, obtained by the nucleophilic substitution of the cysteine residue (Cys797) at the α-carbon of carbonyl subunit (Fig. 4A). As can be seen in Table 2, the reaction enthalpy for the formation of the enzyme-inhibitor complex of **7j** is much more favorable than that of **7k**, in qualitative accordance with the greater activity of the former.

**Table 2:** Calculated enzyme-inhibitor reaction relative enthalpies (kcal/mol) according to the reaction depicted in Fig. 6 (PM7 method, dielectric constant = 78.4).

Ligand	Relative Enthalpy
According to scheme 2A	
**7j**	0
**7k**	61.46
According to scheme 2B	
**7h**	0
**7i**	22.56
**7l**	2.61

In the same way for compounds **7h**, **7i** and **7l**, with Michael acceptor groups, the calculated enthalpies generated from the enzyme-inhibitor complexes (Fig. S4, supplementary material) were analyzed. The relative reaction enthalpies for the formation of the ligand-enzyme adducts were obtained from the nucleophilic attack of the cysteine residue (Cys797) at the electrophilic carbon of the enone subunit (Fig. 4B). The Michael complex formed from compound **7h** is slightly more stable than the complex generated from compound **7l** and both are much more stable than the one produced from compound **7i** (Table 2). Once again, the reaction enthalpy data are in qualitative accordance with the experimental results: the lower stability of the enzyme-inhibitor complex from compound **7i** may explain its lower activity, since the unfavorable reaction enthalpy would contribute to reduce the formation of the enzyme-inhibitor complex in comparison with compounds **7l** and **7h**.

**Figure 5:**
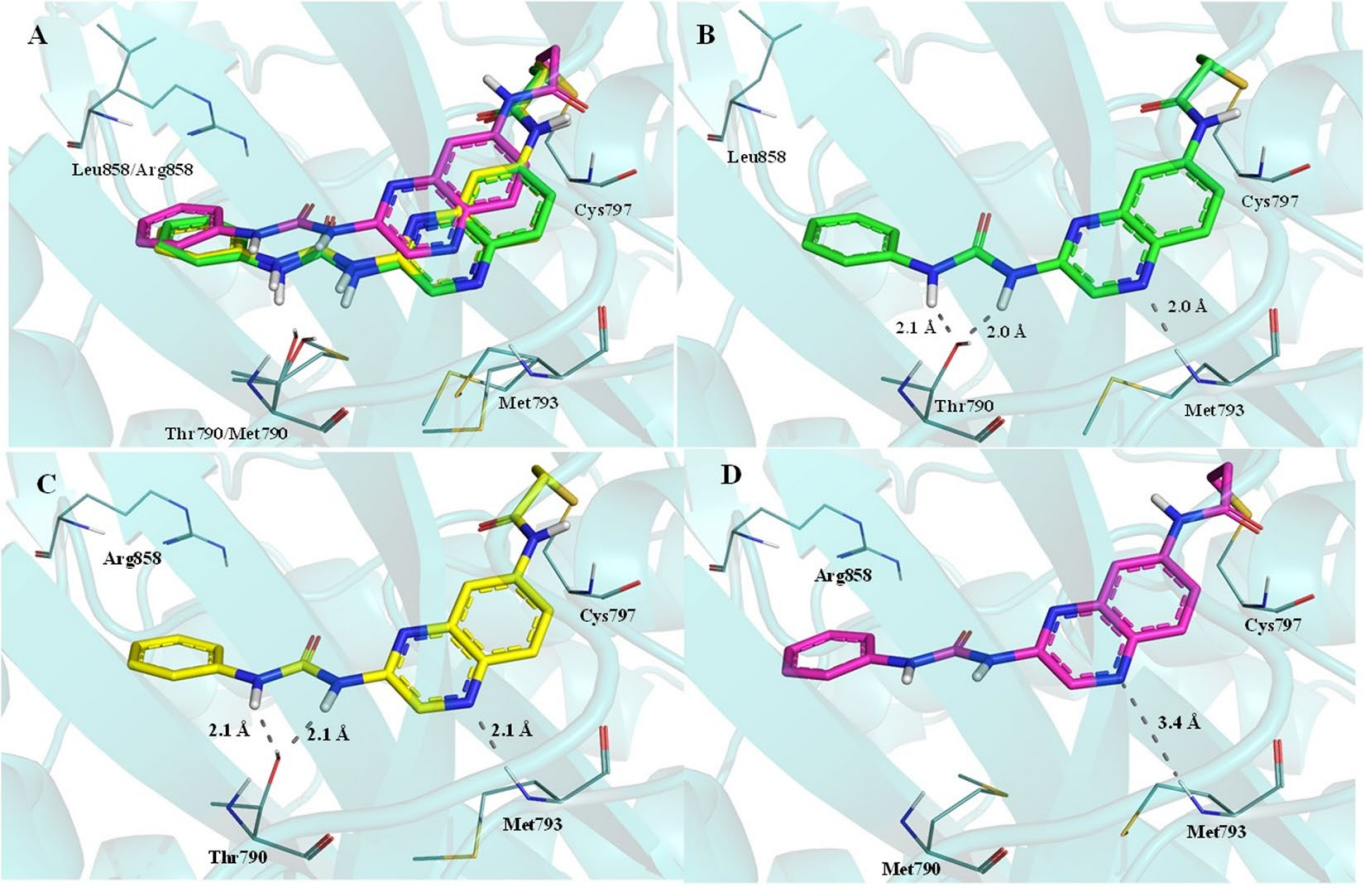
Compound **7h** interaction profile in the ATP binding site of EGFR_wt_, EGFR_L858R_ and EGFR_L858_R_/T790M_ (**A**). Interaction profile of compound **7h** in EGFR_wt_ (*carbon atoms in green*, **B**), in EGFR_L858R_ (*carbon atoms in yellow*, **C**) and in EGFR_L858R/T790M_ (*carbon atoms in magenta*, **D**); *Dashed gray lines*: Hydrogen bonds.

Additionally, covalent docking studies with the quinoxaline ureas **7h** and **7l** in the ATP binding site of EGFR_wt_, EGFR_L858R_ and EGFR_L858R/T790M_ were performed in an attempt to provide a structural rationale for the differences observed in their activities against these enzymes. Strong binding interactions in EGFR_wt_ and EGFR_L858R_ were observed for compound **7h**, involving the NH group of the hinge’s Met793 and the OH group of Thr790 (Fig. 5). On the other hand, no interaction with Met790 was observed for compound **7h** in the ATP binding site of EGFR double mutant form (i.e. EGFR_L858R/T790M_), although a weak interaction has been observed with Met793, probably due to the absence of the Thr790 hydroxyl group and the steric hindrance of the Met790 side chain (Fig. 5). These results can explain the equipotency of **7h** in inhibiting EGFR_wt_ and EGFR_L858R_, and its lower activity against EGFR_L858R/T790M_.

**Figure 6:**
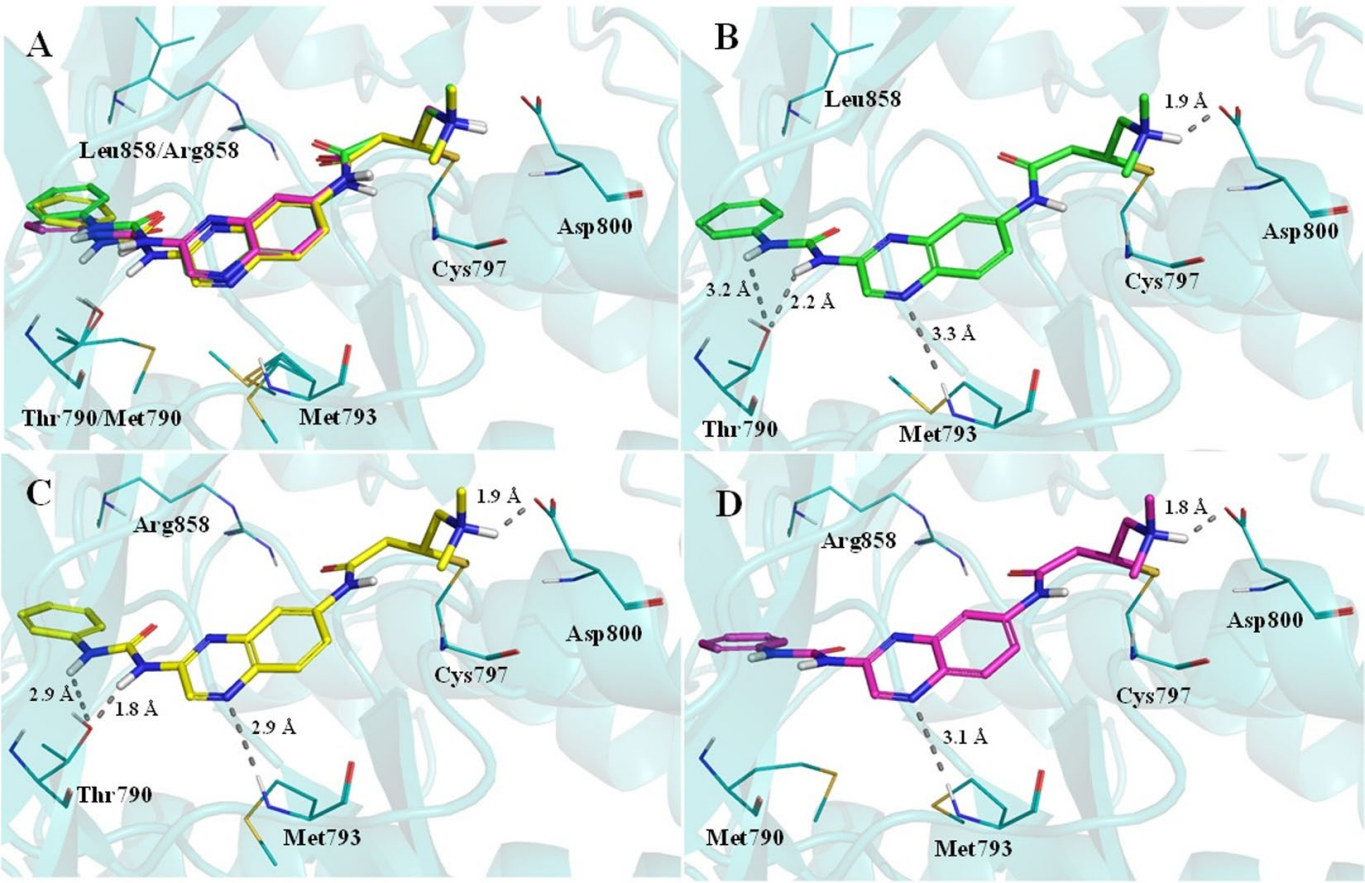
Compound **7l** interaction profile in the ATP binding site of EGFR_wt_, EGFR_L858R_ and EGFR_L858_R_/T790M_ (**A**). Interaction profile of compound **7l** in EGFR_wt_ (*carbon atoms in green*, **B**), in EGFR_L858R_ (*carbon atoms in yellow*, **C**) and in EGFR_L858R/T790M_ (*carbon atoms in magenta*, **D**). *Dashed gray lines*: Hydrogen bonds.

As depicted in Fig. 6, covalent docking showed that urea **7l** binds properly and similarly in EGFR_wt_, EGFR_L858R_ and EGFR_L858R/T790M_. The quinoxaline’s nitrogen makes a similar interaction with the Met793, and the quaternary amine establishes ionic interactions with Asp800. The urea subunit was able to interact with Thr790 hydroxyl group only in the EGFR_wt_ and EGFRL_858R_ forms (Fig. 6). The covalent adducts formed between Cys797 and compound **7l**, for all the EGFR forms, are presented in Fig. 6.

### Cellular panel screening

Patient-derived tumor cells proved to be an important approach to improve predictability of effectiveness during future drug development clinical phases^47,48^. Based on this premise, the inhibitory activity of compounds **7a-m** towards a patient-derived tumor cell panel^49^ was evaluated, using a single screening concentration of 10 μM. Despite its nanomolar potency against the three EGFR forms, compound **7l** did not inhibit the Oncotest® tumor cell lines’ growth in a significant manner. These results suggest permeation issues for compound **7l**. In fact, it is worth mentioning that, although the data observed in a cellular assay have no direct correlation to EGFR enzymatic tests, this phenomenon is not unusual and is related to the nature of the assays itself. In enzymatic assays there is no influence of membrane permeability issues, while in cell viability assays compounds must penetrate the cell membrane and resist the cellular environment for long enough for the ligand-receptor interaction to occur and to trigger the biological activity^50^. The most potent EGFR_wt_ and EGFR_L858R_ inhibitor, **7h**, showed important cytotoxic activity. It displayed a moderate growth inhibition effect (55–77%) upon sensible (GXF 251L, CXF DiFi and LXFA PC9) and first generation EGFR inhibitor resistant (LXFL 529L and LXFA NCI-H1975) patient-derived tumor cell lines (Table 3).

**Table 3:** Cytotoxic activity on a patient-derived (PD) tumor cell line panel determined for compound **7h** at 10 μM. Results are expressed by cellular growth inhibition percentage related to negative control.

Tumor type	PD tumor cell line	% inhibition	Tumor type	PD tumor cell line	% inhibition
Stomach	GXF 251L^b^	55%	Breast	MAXF 401NL	72%
	GXA MKN45	8%		MAXF MDA231	67%
Colon	CXF DIFI^b^	77%		MAXF MCF7	76%
	CXF Colo205	56%	Bladder	BXF 1218L	84%
	CXF SW620	82%		BXF T24	76%
	CXF RKO	82%	Kidney	RXF 486L	86%
	CXF HCT116	92%		RXF 786-O	80%
Lung	LXFA PC9^b^	75%	Glioblastoma	CNXF A172	86%
	LXFA NCI-H1975^c^	68%	Prostate	PRXF DU145	91%
	LXFL H460	33%	Uterus	UXF 1138L	91%
	LXFL 529L^a^	74%	Liver	LIXF 575L	40%
	LXFA 629L	76%	Ovary	OVXF 899L	87%
Melanoma	MEXF 276L	88%	Pancreas	PAXF 546L	58%
	MEXF 1737l	15%		PAXF 1657l	89%
	MEXF 1539L	93%	Sarcoma	SXF1301L	89%

^a^EGFR overexpressed, ^b^EGFR_del-exon19_ expressed, ^c^EGFR_wt_ expressed.

## Conclusions

A novel and original scaffold was identified for the wild-type and clinically relevant mutant forms of EGFR inhibition. Among the compounds herein described, **7h** is a potent inhibitor of EGFR_**wt**_ and E.GFR_**L858R**_ in the nanomolar range and showed cellular cytotoxic activity (>80% at 10 µM) on Oncotest® patient-derived tumor cell lines, while being selective for EGFR related cells. Molecular docking and reaction enthalpy calculations have shown the influence of the combination of reversible and covalent binding modes with EGFR on the inhibitory activity.

## Methods

### Synthesis and characterization of compounds

All commercially available reagents and solvents were used without further purification, except compound 7-nitroquinoxalin-2-amine (**8**) synthetized as previously described^41^. ^1^H and ^13^C nuclear magnetic resonance (NMR) and DEPT135 spectra were determined in DMSO-d_6_ or pyridine-d_5_ solutions using a Bruker AC-200 or a Bruker Avance 400 spectrometer. NOESY 1D experiments were determined using a Varian Unity-300 spectrometer. The chemical shifts are indicated in parts per million (δ) from solvent residual peaks and the coupling constant values (*J*) are indicated in Hz. Signal multiplicities are represented by: s (singlet), d (doublet), dd (double doublet), t (triplet), dt (double triplet), m (multiplet) and br (broad signal). Infrared spectra were obtained using a Thermo Scientific Nicolet’s Avatar iS10 spectrometer equipped with smart endurance diamond ATR unit for direct measurements. Mass spectra were obtained from a TLC-MS interface CAMAG in negative mode and from a Hewlett Packard HP 5973 mass selective detector (70 eV). Melting points (m.p.) were determined using a MP70 Mettler Toledo and are uncorrected. The purity of compounds was determined by HPLC (Merck Hitachi L- 6200 intelligent pump, Merck Hitachi AS-2000 auto sampler, Merck Hitachi L-4250 UV vis detector) using a ZORBAX Eclipse XDB C8 column (5 mm), employing a gradient of 0.01 M KH_2_PO_4_ (pH 2.3) and methanol as solvent system with a flow rate of 1.5 mL/min and detection at 254 nm.

### General methodology for the synthesis of 1-(7-nitroquinoxalin-2-yl)-3-phenylureas (**9a-d**)

To a round bottom flask containing a suspension of 7-nitroquinoxalin-2-amine (0.5 g, 2.5 mmol) in dry toluene, one equivalent (2.5 mmol) of substituted phenylisocyanate was added. Suspension was kept in refluxing conditions for 2–4 hours. After reactant consumption, at room temperature, solids were filtered and washed with toluene to obtain salmon color solids.

### 1-(7-nitroquinoxalin-2-yl)-3-(4-chloro-3-(trifluormethyl)phenyl)urea (9a)

Compound **9a** was synthetized via condensation of **8** with 4-chloro-3-(trifluoromethyl)phenyl isocyanate resulting in a salmon powder with 82% yield. Melting point (m.p.) was 266–269 °C. ^1^H NMR (200 MHz, DMSO-d_6_) δ (ppm): 10.90 (1H, s), 10.75 (1H, s), 9.14 (1H, s), 8.91 (1H, d, *J* = 4 Hz), 8.32 (1H, dd), 8.30 (1H, d, *J* = 4 Hz,), 8.19 (1H, d, *J* = 9 Hz), 7.91 (1H, dd), 7.70 (1H, d, *J* = 9 Hz). ^1^H NMR (200 MHz, piridine-d_5_) δ (ppm): 11.82 (1H, s), 9.29 (1H, s), 8.91 (1H, d, *J* = 2 Hz), 8.70 (1H, d, *J* = 2 Hz), 8.40 (1H, dd, J = 2, 10 Hz), 8.26 (1H, d, *J* = 8 Hz), 8.00 (1H, d, *J* = 8 Hz), 7.57 (1H, d, *J* = 8 Hz), 4.98 (br, exchange with D_2_O). ^13^C NMR (50 MHz, piridine-d_5_) δ (ppm): 153.0, 150.8, 149.3, 143.9, 142.0, 139.2, 139.0, 136.3, 132.8, 131.5, 129.2, 128.6, 124.3, 123.7, 121.4, 120.0–119.7. IR (ATR: cm^−1^): 2971, 1702, 1589, 1538, 1347, 737. MS: ESI-: *m/z* 410.2 [M-1]-; 412.2 [M + 2-1]-.

### 1-(7-nitroquinoxalin-2-yl)-3-(3-(trifluormethyl)phenyl)urea (9b)

Compound **9b** was synthetized via condensation of **8** with 3-(trifluoromethyl)phenyl isocyanate resulting in a salmon powder with 65% yield. m.p. was 250–252 °C. ^1^H NMR (200 MHz, DMSO-d6) δ (ppm): 10.84 (1H, s), 10.71 (1H, s), 9.17 (1H, s), 8.90 (1H, d, *J* = 4 Hz), 8.33 (1H, dd), 8.20 (2H, m), 7,85 (1H, d, *J* = 7 Hz), 7.61 (1H, t, *J* = 8 Hz), 7.45 (1H, d, *J* = 8 Hz). ^13^C NMR (50 MHz, DMSO-d6) δ (ppm): 151.7, 148.7, 148.1, 143.1, 143.0, 140.8, 139.1, 138.5, 130.4, 130.0, 129.3, 123.8, 122.9, 120.6, 119.8, 115.7. IR (ATR: cm^−1^): 3072, 2968, 1695, 1620, 1545, 1348, 728. MS: ESI-: *m/z* 376.2 [M-1]-.

### 1-(7-nitroquinoxalin-2-yl)-3-phenylurea (9c)

Compound **9c** was synthetized via condensation of **8** with phenyl isocyanate resulting in a salmon powder with 67% yield. m.p. was 253–255 °C. ^1^H NMR (200 MHz, DMSO-d_6_) δ (ppm): 10.63 (1H, s), 10.59 (1H, s), 9.16 (1H, s), 8.83 (1H, d, *J* = 2 Hz), 8.35 (1H, dd), 8.21 (1H, d, J = 8 Hz), 7.68 (2H, d, *J* = 8 Hz), 7.38 (2H, t, *J* = 8 Hz), 7.11 (1H, t, *J* = 8 Hz). ^13^C NMR and DEPT 135 (50 MHz, DMSO-d_6_) δ (ppm): 151.5, 148.8, 148.1, 143.2, 140.7, 138.5, 138.2, 130.5, 128.9, 123.5, 122.7, 120.5, 119.8. IR (ATR: cm^−1^): 3069, 2974, 1690, 1617, 1538, 1343, 737. MS: ESI-: *m/z* 308.2 [M-1]-.

### 1-(3-chloro-4-fluorophenyl)-3-(7-nitroquinoxalin-2-yl)urea (9d)

Compound **9d** was synthetized via condensation of **8** with 3-chloro-4-fluorophenyl isocyanate resulting in a salmon powder with 68% yield. m.p. was 253–256 °C.^1^H NMR (200 MHz, DMSO-d_6_) δ(ppm): 10.81 (1H, s), 10.73 (1H, s), 9.10 (1H, s), 8.95 (1H, d, *J* = 4 Hz), 8.35 (1H, dd, *J* = 2 and 9 Hz), 8.20 (1H, d, *J* = 9 Hz), 8.02 (1H, dd, *J* = 2 and 7 Hz), 7.69–7.61 (1H, m), 7.43 (1H, t, *J* = 9 Hz). ^13^C NMR (50 MHz, DMSO-d_6_) δ (ppm): 151.7, 148.6, 148.1, 143.2, 140.7, 138.4, 135.4, 135.4, 130.4, 122.9, 121.5, 120.7, 120.6, 119.5, 119.1, 117.2, 116.7. IR (ATR: cm^−1^): 2986, 1694, 1612, 1542, 1345, 728. MS: ESI-: *m/z* 360.2 [M-1]-; 362.2 [M + 2–1].

### General methodology for the synthesis of 1-(7-aminoquinoxalin-2-yl)-3-phenylureas (**10a-d**)

In a round bottom flask, derivatives **9a-d** and five equivalent of tin (II) chloride dihydrate (SnCl_2_.2H_2_O) in ethanol were refluxed for 17 hours. After the nitro group reduction to an aniline group, the solvent was evaporated under reduced pressure, sodium carbonate solution was used to adjust pH to 7, ethyl acetate was added for product extraction by the organic phase, further dried with anhydrous sodium sulfate, filtered and reduced to furnish anilines as yellow solids.

### 1-(7-aminoquinoxalin-2-yl)-3-(4-chloro-3-(trifluormethyl)phenyl) urea (10a)

Compound **10a** was synthetized via reduction of **9a** with SnCl_2_.2H_2_O resulting in a yellow powder with 91% yield and the m.p. was >300 °C. ^1^H NMR (200 MHz, DMSO-d_6_) δ (ppm): 11.40 (1H, s), 10.21 (1H, s), 8.51 (1H, s), 8.24 (1H, d, *J* = 2 Hz), 7.76–7.72 (2H, m), 7.63 (1H, d, *J* = 9 Hz), 7.03 (1H, dd), 6.82 (1H, d, *J* = 2 Hz), 6.07 (2H, br). ^13^C NMR and DEPT 135 (100 MHz, DMSO-d_6_) δ (ppm): 152.1, 151.4, 147.2, 141.1, 138.2, 136.8, 132.4, 132.3, 132.1, 129.4, 129.4, 127.9, 124.1, 123.6, 121.4, 119.0, 103.9. IR (ATR: cm^−1^): 3219, 2980, 1691, 1621, 1573, 745. MS: ESI-: *m/z* 380.2 [M-1]-; 382.2 [M + 2–1]-.

### 1-(7-aminoquinoxalin-2-yl)-3-(3-(trifluormethyl)phenyl)urea (10b)

Compound **10b** was synthetized via reduction of **9b** with SnCl_2_.2H_2_O resulting in a yellow powder with 82% yield and the m.p. was 257–261 °C. ^1^H NMR (200 MHz, DMSO-d_6_) δ (ppm): 11.43 (1H, s), 10.18 (1H, s), 8.51 (1H, s), 8.14 (1H, s), 7.69–7.61 (3H, m), 7.43 (1H, d, *J* = 8 Hz), 7.03 (1H, dd, *J* = 2 and 9 Hz), 6.83 (1H, d, *J* = 2 Hz), 6.06 (2H, br). ^13^C NMR (100 MHz, DMSO-d_6_) δ (ppm): 152.2, 151.4, 147.3, 141.1, 139.6, 132.3, 130.1, 129.7, 129.4, 128.9, 122.8, 119.2, 118.9, 115.0, 104.1, 103.8. IR (ATR: cm^−1^): 3361, 3222, 2986, 1692, 1621, 1567, 746. MS: ESI-: *m/z* 347.2 [M-1]-.

### 1-(7-aminoquinoxalin-2-yl)-3-phenylurea (10c)

Compound **10c** was synthetized via reduction of **9c** with SnCl_2_.2H_2_O resulting in a yellow powder with 61% yield and the m.p. was 243–247 °C. ^1^H NMR (200 MHz, DMSO-d_6_) δ (ppm): 11.35 (1H, s), 10.14 (1H, s), 8.54 (1H, s), 7.64–7.57 (3H, m), 7.35 (1H, t, *J* = 8 Hz), 7.06 (1H, t, *J* = 8 Hz), 7.03 (1H, dd, *J* = 2 and 8 Hz), 6.81 (1H, d, *J* = 2 Hz), 6.04 (1H, br). ^13^C NMR and DEPT 135 (50 MHz, DMSO-d_6_) δ (ppm): 152.1, 151.4, 147.6, 141.2, 138.8, 132.5, 132.2, 129.4, 129.0, 122.9, 118.9, 118.6 (C6), 103.8. IR (ATR: cm^−1^): 3463, 3339, 3217, 2984, 1687, 1627, 1562, 750. MS: ESI-: *m/z* 278.2 [M-1].

### 1-(7-aminoquinoxalin-2-yl)-3-(3-chloro-4-fluorophenyl) urea (10d)

Compound **10d** was synthetized via reduction of **9d** with SnCl_2_.2H_2_O resulting in a yellow powder with 37% yield. ^1^H NMR (200 MHz, DMSO-d_6_) δ (ppm): 11.36 (1H, s), 10.18 (1H, s), 8.46 (1H, s), 7.92 (1H, dd, *J* = 2 and 7 Hz), 7.62 (1H, d, *J* = 9 Hz), 7.48–7.41 (2H, m), 7.05 (1H, dd, *J* = 2 and 9 Hz), 6.83 (1H, d, *J* = 2 Hz), 6.05 (2H, br). IR (ATR: cm^−1^): 3461, 3333, 3217, 2981, 1693, 1622, 1564, 739. MS: ESI-: *m/z* 330.2 [M-1]-; 332.2 [M + 2–1]-.

### General methodology for the synthesis of N-(3-(3-phenylureido)quinoxalin-6-yl) amide derivatives

Anilines **10a-d** were solubilized in dry THF and 1.1 equivalent of *N,N*-diisopropylethylamine (DIPEA) was added in a round bottom flask under argon atmosphere. Then, corresponding acyl chloride (1 equivalent) was dropwise added into the mixture. The reaction mixture was kept in ice cold bath until the total conversion of reagents to products. Isolation and purification of compounds were performed by flash chromatography (mobile phases: dichloromethane: methanol 4–8%; dichloromethane: ethyl acetate 10–90%; ethyl acetate: methanol: ammonia in methanol 9:0.8:0.2 or ethyl acetate: methanol 10%).

### *N*-(3-(3-(4-chloro-3-(trifluoromethyl)phenyl)ureido)quinoxalin-6-yl) acrylamide (7a)

Compound **7a** was synthetized via condensation of **10a** with acryloyl chloride resulting in a white powder with 70% yield after flash chromatography (mobile phase: DCM:MeOH 4–8%) and the m.p. was 272–274 °C. ^1^H NMR (200 MHz, DMSO-d_6_) δ (ppm): 10.88 (1H, s), 10.60 (1H, s), 10.37 (1H, s), 8.97 (1H, s), 8.38 (1H, d, *J* = 2 Hz), 8.23 (1H, s), 7.95 (1H, d, *J* = 10 Hz), 7.81 (1H, dd, *J* = 2 and 9 Hz), 7.72 (2H, s), 6.46 (1H, dd, *J* = 10 and 17 Hz), 6.35 (1H, dd, *J* = 2 and 17 Hz), 5.85 (1H, dd, *J* = 2 and 9 Hz). ^13^C NMR (50 MHz, DMSO-d_6_) δ (ppm): 163.7, 151.9, 147.6, 140.9, 139.8, 138.1, 137.5, 135.4, 132.2, 131.5, 129.2, 127.9–126.6, 124.2, 123.8, 120.7, 120.0, 117.8, 114.2. IR (ATR: cm^−1^): 2924, 1687, 1584, 1130, 1032. MS: ESI-: *m/z* 434.2 [M-1]-; 436.2 [M + 2–1]-. Purity (HPLC at 254 nm; R.T.): 100%; 8.37 minutes.

### (*E*)-*N*-(3-(3-(4-chloro-3-(trifluoromethyl)phenyl)ureido)quinoxalin-6-yl)but-2-enamide (7b)

Compound **7b** was synthetized via condensation of **10a** with crotonyl chloride resulting in a salmon powder with 37% yield after flash chromatography (mobile phase: DCM:MeOH 4–8%) and the m.p. was 277–280 °C. ^1^H NMR (200 MHz, DMSO-d_6_) δ (ppm): 10.90 (1H, s), 10.40 (1H, s), 10.34 (1H, s), 8.95 (1H, s), 8.35 (1H, d, *J* = 2 Hz), 8.22 (1H, s), 7.93 (1H, d, *J* = 10 Hz), 7.78 (1H, dd, *J* = 2 and 10 Hz), 7.72 (2H, s), 6.88 (1H, dd, *J* = 7 and 15 Hz), 6.19 (1H, dd, *J* = 2 and 15 Hz), 1.90 (3H, dd, *J* = 1,5 and 7 Hz). ^13^C NMR (50 MHz, DMSO-d_6_) δ (ppm): 164.0, 151.9, 147.6, 141.2, 141.0, 139.8, 138.1, 137.3, 135.2, 132.2, 129.1, 127.2–126.6, 125.7, 124.1, 123.8, 120.7, 117.8, 113.9, 17.6. IR (ATR: cm^−1^): 3266, 2971, 1695, 1675, 1641, 1618, 1582, 1124, 1031. MS: ESI-: *m/z* 448.1 [M-1]-; 450.1 [M + 2–1]-. Purity (HPLC at 254 nm; R.T.): 100.0%; 8.75 minutes.

### 2-chloro-*N*-(3-(3-(4-chloro-3-(trifluoromethyl)phenyl)ureido)quinoxalin-6-yl)acetamide (7c)

Compound **7c** was synthetized via condensation of **10a** with chloroacethyl chloride resulting in a pearl powder with 38% yield after flash chromatography (mobile phase: DCM: AcOEt 10–90%) and the m.p. was 253–255 °C. ^1^H NMR (200 MHz, DMSO-d_6_) δ (ppm): 10.88 (1H, s), 10.76 (1H, s), 10.39 (1H, s), 8.99 (1H, s), 8.29 (1H, d, *J* = 2 Hz), 8.21 (1H, s), 7.97 (1H, d, *J* = 8 Hz), 7.75 (3H, m), 4.36 (2H, s). ^13^C NMR (50 MHz, DMSO-d_6_) δ (ppm): 165.3, 151.9, 147.7, 140.3, 139.7, 138.1, 137.8, 135.4, 132.2, 129.3, 127.2–126.6, 124.2, 123.8, 120.6, 117.9, 114.3, 43.6. IR (ATR: cm^−1^): 3335, 2962, 1687, 1615, 1584, 1130,1030. MS: ESI-: *m/z* 456.0 [M-1]-; 458.0 [M + 2–1]-. Purity (HPLC at 254 nm; R.T.): 97.0%; 8.60 minutes.

### *N*-(3-(3-(4-chloro-3-(trifluoromethyl)phenyl)ureido)quinoxalin-6-yl)-2-cyanoacetamide (7d)

Compound **7d** was synthetized via condensation of **10a** with 2-cyanoacethyl chloride resulting in a pearl powder with 17% yield after flash chromatography (mobile phase: DCM:AcOEt 10–90%) and the m.p. was 243–246 °C. 1H NMR (200 MHz, DMSO-d_6_) δ (ppm): 10.85 (1H, s), 10.76 (1H, s), 10.38 (1H, s), 8.99 (1H, s), 8.23 (2H, m), 7.95 (1H, d, *J* = 9 Hz), 7.70 (3H, m), 4.02 (2H, s). ^13^C NMR (50 MHz, DMSO-d_6_) δ (ppm): 161.9, 151.9, 147.7, 140.2, 139.7, 138.1, 137.9, 135.4, 132.2, 129.4, 127.2–126.6, 124.1, 123.8, 120.4, 120.0, 117.9, 115.7, 114.3, 27.1. IR (ATR: cm^−1^): 3336, 2971, 2264, 1694, 1596, 1127, 1032. MS: ESI-: *m/z* 447.0 [M-1]-; 449.0 [M + 2–1]-. Purity (HPLC at 254 nm; R.T.): 97.4%; 7.64 minutes.

### (*E*)-*N*-(3-(3-(4-chloro-3-(trifluoromethyl)phenyl)ureido)quinoxalin-6-yl)-4-(dimethylamino)but-2-enamide (7e)

Compound **7e** was synthetized via condensation of **10a** with 4-(dimethylamino)but-2-enamide chloride resulting in a pearl powder with 23% yield after flash chromatography (mobile phase: AcOEt:MeOH:NH_3_ in MeOH 9:0.8:0.2) and the m.p. was 217–220 °C. ^1^H NMR (200 MHz, DMSO-d_6_) δ (ppm): 10.88 (1H, s), 10.50 (1H, s), 10.34 (1H, s), 8.96 (1H, s), 8.37 (1H, d, *J* = 2 Hz), 8.22 (1H, s), 7.94 (1H, d, *J* = 9 Hz), 7.78 (1H, dd, *J* = 2 and 9 Hz), 7.22 (1H, s), 6.89–8.76 (1H, dt, *J* = 6 and 16 Hz), 6.34 (1H, d, *J* = 16 Hz), 3.09 (2H, d, *J* = 6 Hz), 2.20 (6H, s). ^13^C NMR (150 MHz, DMSO-d^6^) δ (ppm): 163.1, 151.9, 147.6, 140.8, 140.7, 139.9, 138.2, 137.5, 135.4, 132.2, 129.2, 127.0–126.9, 125.5, 124.0, 123.7, 121.8, 120.7, 117.7, 114.2, 58.1, 43.4 MS: ESI-: *m/z* 491.3 [M-1]-; 493.,3 [M + 2–1]-. Purity (HPLC at 254 nm; R.T.): 100.0%; 5.90 minutes.

### *N*-(3-(3-(3-(trifluoromethyl)phenyl)ureido)quinoxalin-6-yl)acrylamide (7f)

Compound **7f** was synthetized via condensation of **10b** with acryloyl chloride resulting in a pearl powder with 41% yield after flash chromatography (mobile phase: DCM: AcOEt 10–90%) and the m.p. was 268–271 °C. ^1^H NMR (200 MHz, DMSO-d_6_) δ (ppm): 10.89 (1H, s), 10.61 (1H, s), 10.34 (1H, s), 8.98 (1H, s), 8.38 (1H, d, *J* = 2 Hz), 8.15 (1H, s), 7.96 (1H, d, *J* = 9 Hz), 7.83 (1H, dd, *J* = 2 and 9 Hz), 7.79–7.62 (2H, m), 7.44 (1H, d, *J* = 8 Hz), 6.50 (1H, dd, *J* = 9 and 17 Hz), 6.35 (1H, dd, *J* = 2 and 17 Hz), 5.85 (1H, dd, *J* = 2 and 9 Hz). ^13^C NMR (50 MHz, DMSO-d_6_) δ (ppm): 163.7, 152.0, 147.7, 140.9, 139.8, 139.4, 137.6, 135.3, 131.6, 130.2, 129.4, 129.2, 127.9, 122.9, 121.4, 120.7, 117.8, 115.1, 114.2. IR (ATR: cm^−1^): 3271, 2976, 1687, 1666, 1625, 1577, 1120. MS: ESI-: *m/z* 400.3 [M-1]-. Purity (HPLC at 254 nm; R.T.): 100%; 7.78 minutes.

### 2-chloro-*N*-(3-(3-(3-(trifluoromethyl)phenyl)ureido)quinoxalin-6-yl)acetamide (7g)

Compound **7g** was synthetized via condensation of **10b** with chloroacethyl chloride resulting in a white powder with 45% yield after flash chromatography (mobile phase: DCM:AcOEt 10–90%) and the m.p. was 260–263 °C. ^1^H NMR (200 MHz, DMSO-d_6_) δ (ppm): 10.89 (1H, s), 10.76 (1H, s), 10.36 (1H, s), 8.99 (1H, s), 8.28 (1H, d, *J* = 2 Hz), 8.14 (1H, s), 7.97 (1H, d, *J* = 9 Hz), 7.75 (1H, dd, *J* = 2 and 9 Hz), 7.67–7.57) (2H, m), 7.44 (1H, d, *J* = 7 Hz), 4.36 (2H, s). ^13^C NMR (50 MHz, DMSO-d_6_) δ (ppm): 165.3, 151.9, 147.8, 140.3, 139.7, 139.4, 137.8, 135.4, 130.2, 130.0, 129.4, 129.3, 122.8, 120.5, 115.2, 114.3, 105.7, 43.64. IR (ATR: cm^−1^): 3305, 2970, 1684, 1666, 1621, 1575, 1120.MS: ESI-: *m/z* 422.1 [M-1]-; 423.9 [M + 2–1]-. Purity (HPLC at 254 nm; R.T.): 97,5%; 8.07 minutes.

### *N*-(3-(3-phenylureido)quinoxalin-6-yl)acrylamide (7h)

Compound **7h** was synthetized via condensation of **10c** with acryloyl chloride resulting in a white powder with 38% yield after flash chromatography (mobile phase: DCM: AcOEt 10–90%) and the m.p. was 280–283 °C. ^1^H NMR (200 MHz, DMSO-d_6_) δ (ppm): 10.85 (1H, s), 10.60 (1H, s), 10.27 (1H, s), 8.91 (1H, s), 8.36 (1H, d, J = 2 Hz), 7.95 (1H, d, *J* = 8 Hz), 7.82 (1H, dd, *J* = 2 and 8 Hz), 7.58 (2H, d, *J* = 8 Hz), 7.38 (2H, t, *J* = 8 Hz), 7.09 (1H, t, *J* = 8 Hz), 6.59–6.46 (1H, dd, *J* = 10 and 17 Hz), 6.40–6.30 (1H, dd, *J* = 2 and 16 Hz), 5.88–5.82 (1H, dd, *J* = 2 and 10 Hz). ^13^C NMR and DEPT135 (50 MHz, DMSO-d_6_) δ (ppm): 163.7, 151.8, 147.9, 140.9, 139.6, 138.5, 137.7, 135.1, 131.5, 129.2, 129.1, 127.9, 123.2, 120.4, 119.1, 114.0. IR (ATR: cm^−1^): 3266, 3032, 2977, 1686, 1662, 1621, 1585. MS: ESI-: m/z 332.2 [M-1]-. Purity (HPLC at 254 nm; R.T.): 95.5%; 6.13 minutes.

### (*E*)-*N*-(3-(3-phenylureido)quinoxalin-6-yl)but-2-enamide (7i)

Compound **7i** was synthetized via condensation of **10c** with crotonyl chloride resulting in a white powder with 29% yield after flash chromatography (mobile phase: DCM: AcOEt 10–90%) and the m.p. was 273–276 °C. ^1^H NMR (200 MHz, DMSO-d_6_) δ (ppm): 10.86 (1H, s), 10.40 (1H, s), 10.25 (1H, s), 8.89 (1H, s), 8.34 (1H, d, *J* = 2 Hz), 7.93 (1H, d, *J* = 10 Hz), 7.79 (1H, dd, *J* = 2 and 9 Hz), 7.58 (2H, d, *J* = 7 Hz), 7.38 (2H, t, *J* = 8 Hz), 7.09 (1H, t, *J* = 7 Hz), 6.99–6.81 (1H, m), 6.20 (1H, d, *J* = 16 Hz), 1.90 (3H, d, *J* = 6 Hz). ^13^C NMR (50 MHz, DMSO-d_6_) δ (ppm): 164.0, 151.8, 147.9, 141.2, 141.1, 139.7, 138.5, 137.5, 135.0, 129.1, 129.1, 125.7, 123.2, 120.4, 119.1, 113.8, 17.7. IR (ATR: cm^−1^): 3274, 2974, 1693, 1673, 1636, 1586. MS: ESI-: *m/z* 346.3 [M-1]-. Purity (HPLC at 254 nm; R.T.): 98.5%; 6.58 minutes.

### 2-chloro-*N*-(3-(3-phenylureido)quinoxalin-6-yl)acetamide (7j)

Compound **7j** was synthetized via condensation of **10c** with 2-chloroacethyl chloride resulting in a white powder with 36% yield after flash chromatography (mobile phase: DCM:AcOEt 10–90%) and the m.p. was 249–252 °C. ^1^H NMR (200 MHz, DMSO-d_6_) δ (ppm): 10.83 (1H, s), 10.76 (1H, s), 10.29 (1H, s), 8.93 (1H, s), 8.26 (1H, s), 7.96 (1H, d, *J* = 8 Hz), 7.76 (1H, d, *J* = 8 Hz), 7.58 (2H, d, *J* = 8 Hz), 7.38 (2H, t, *J* = 7 Hz), 7.09 (1H, t, *J* = 7 Hz), 4.36 (2H, s). ^13^C NMR and DEPT 135 (50 MHz, DMSO-d6) δ (ppm): 165.3, 151.8, 148.0, 140.3, 139.6, 138.5, 137.9, 135.2, 129.3, 129.1, 123.2, 120.2, 119.1, 114.2, 43.7. IR (ATR: cm^−1^): 3267, 2980, 1681, 1622, 1585. MS: ESI-: *m/z* 354.1 [M-1]-; 356.1 [M + 2-1]-. Purity (HPLC at 254 nm; R.T.): 96.4%; 6.12 minutes.

### 2-cyano-*N*-(3-(3-phenylureido)quinoxalin-6-yl)acetamide (*7k*)

Compound **7k** was synthetized via condensation of **10c** with 2-cyanoacethyl chloride resulting in a white powder with 12% yield after flash chromatography (mobile phase: DCM:AcOEt 10–90%) and the m.p. was 238–241 °C. ^1^H NMR (200 MHz, DMSO-d_6_) δ (ppm): 10.79 (1H, s), 10.76 (1H, s), 10.26 (1H, s), 8.94 (1H, s), 8.20 (1H, s), 7.96 (1H, d, *J* = 8 Hz), 7.70 (1H, d, *J* = 8 Hz), 7.58 (2H, d, *J* = 8 Hz), 7.38 (2H, t, *J* = 8 Hz), 7.09 (1H, t, *J* = 6 Hz), 4.01 (2H, s). ^13^C NMR (50 MHz, DMSO-d_6_) δ (ppm): 161.9, 151.8, 148.0, 140.2, 139.6, 138.5, 138.0, 135.2, 129.4, 129.1, 123.2, 120.1, 119.1, 115.7, 114.1, 27.1. MS: ESI-: *m/z* 345.3 [M-1]-. Purity (HPLC at 254 nm; R.T.): 98.1%; 5.05 minutes.

### (*E*)-4-(dimethylamino)-*N*-(3-(3-phenylureido)quinoxalin-6-yl)but-2-enamide (7l)

Compound **7l** was synthetized via condensation of **10c** with 4-(dimethylamino)but-2-enamide chloride resulting in a white powder with 18% yield after flash chromatography (mobile phase: AcOEt:MeOH:NH_3_ in MeOH 9:0.8:0.2) and the m.p. was 222–225 °C. ^1^H NMR (200 MHz, DMSO-d_6_) δ (ppm): 10.86 (1H, s), 10.51 (1H, s), 10.26 (1H, s), 8.90 (1H, s), 8.36 (1H, d, *J* = 2 Hz), 7.93 (1H d, *J* = 9 Hz), 7.80 (1H, dd, *J* = 2 and 8 Hz), 7.58 (2H, d, *J* = 7 Hz), 7.38 (2H, t, *J* = 7 Hz), 7.09 (1H, t, *J* = 6 Hz), 6.90–6.77 (1H, m), 6.34 (1H, d, *J* = 14 Hz), 3.09 (2H, d, *J* = 6 Hz), 2.20 (6H, s). ^13^C NMR and DEPT 135 (50 MHz, DMSO-d_6_)δ(ppm): 163.8, 151.8, 147.9, 142.6, 141.1, 139.7, 138.5, 137.6, 135.1, 129.2, 129.1, 125.6, 123.2, 120.4, 119.1, 113.9, 59.8, 45.2. IR (ATR: cm^−1^): 3228, 2976, 1687, 1636, 1586. MS: ESI-: *m/z* 389.4 [M-1]-. Purity (HPLC at 254 nm; R.T.): 95.5%; 3.16 minutes.

### *N*-(3-(3-(3-chloro-4-fluorophenyl)ureido)quinoxalin-6-yl) acrylamide (7m)

Compound **7m** was synthetized via condensation of **10d** with acryloyl chloride resulting in a white powder with 24% yield after flash chromatography (mobile phase: DCM: AcOEt 10–90%) and the m.p. was 259–262 °C. ^1^H NMR (200 MHz, DMSO-d_6_) δ (ppm): 10.83 (1H, s), 10.59 (1H, s), 10.34 (1H, s), 8.91 (1H, s), 8.35 (1H, d, *J* = 2 Hz), 7.92 (2H, s e dd, *J* = 8 Hz), 7.82 (1H, dd, *J* = 2 and 9 Hz), 7.44 (1H, d, *J* = 8 Hz), 6.52 (1H, dd, *J* = 10 and 17 Hz), 6.34 (1H, dd, *J* = 17 and 2 Hz), 5.85 (1H, dd, *J* = 2.5 and 10 Hz). ^13^C NMR (50 MHz, DMSO-d_6_) δ (ppm): 163.7, 151.9, 147.7, 140.9, 139.6, 137.6, 135.8, 135.7, 135.2, 131.5, 129.2, 127.9, 120.7, 120.6, 119.6, 119.3, 117.3, 116.9, 114.2. IR (ATR: cm^−1^): 3281, 2981, 1686, 1667, 1614, 1572. MS: ESI-: *m/z* 384.3 [M-1]-; 386.3 [M + 2-1]-. Purity (HPLC at 254 nm; R.T.): 100%; 7.68 minutes.

### Biochemical assay (TR-FRET)

IC_50_ determinations for EGFR and its mutants (Carna Biosciences, lot13CBS-0005K for EGFRwt; Carna, lot13CBS-0537B for EGFR-L858R and Carna, lot12CBS-0765B for EGFR-L858R/T790M) were performed with the HTRF KinEASE-TK assay from Cisbio according to the manufacturer’s instructions. Briefly, the amount of EGFR in each reaction well was set to 0.60 ng EGFR wild-type (0.67 nM), 0.10 ng EGFR L858R (0.11 nM) or 0.07 ng EGFR T790M/L858R (0.08 nM), respectively. An artificial substrate peptide (TK-substrate from Cisbio) was phosphorylated by EGFR. After completion of the reaction (reaction times: 25 min for wild-type, 15 min for L858R, 20 min for T790M/L858R), the reaction was stopped by addition of a buffer containing EDTA as well as, an anti-phosphotyrosine antibody labeled with europium cryptate and streptavidin labeled with the fluorophore XL665. FRET between europium cryptate and XL665 was measured after an additional hour of incubation to quantify the phosphorylation of the substrate peptide. ATP concentrations were set at their respective *K*_m_-values (9.5 μM for EGFR-wt, 9 μM for EGFR-L858R and 4 μM for EGFR-L858R/T790M) while a substrate concentration of 1 μM, 225 nM and 200 nM, respectively, was used. Kinase and inhibitor were preincubated for 30 min (EGFR-wt) and 1 h (EGFR-L858R and EGFR-L858R/T790M) before the reaction was started by addition of ATP and substrate peptide. An EnVision multimode plate reader (Perkin Elmer) was used to measure the fluorescence of the samples at 620 nm (Eu-labeled antibody) and 665 nm (XL665 labeled streptavidin) 50 μs after excitation at 320 nm. The quotient of both intensities for reactions made with eight different inhibitor concentrations was then analyzed using the Quattro Software Suite for IC_50_-determination. Each reaction was performed in duplicate, and at least three independent determinations of each IC_50_ were made.

### Molecular docking

All the compounds were constructed with Spartan’16 (Key ID: 713413641076066525). A Monte Carlo conformational search was performed with the molecular mechanics method MMFF (Merck Molecular Force Field) and the geometry of the lowest energy conformer of each compound was re-optimized with the semi-empirical method PM6^51^. For compounds **7e** and **7l**, the amino group was also considered in the protonated form.

The EGFR_wt_ crystallographic structure available in the Protein Data Bank with code 4G5J (resolution: 2.8 Å. in complex with Afatinib)^52^ was used for docking runs with the GOLD 5.4 program (Validation code: 44d6-05f1-f186-5a7f-190c-7fec-7eff-bb85-9e39). The default fitness score function ChemPLP^53^ was evaluated for re-docking of the co-crystallized ligand (Afatinib). Crystallographic water molecules were removed during the docking runs, and the binding site was determined within 6 Å around the ligand (Afatinib) in complex with 4G5J. The covalent docking mode was used with the protein link at Cys797 with the ligand link atom at the α or β carbon to the carbonyl group, depending on the ligand structure.

Docking runs were performed in triplicate and the poses presenting the best scores were analyzed to identify potential interactions with amino acid residues of the enzyme’s binding site. The amino acids with more favorable interactions were selected and a semi-rigid docking was performed, which allowed for flexibility of the side chains of Glu762A, Met766A, Leu788A, Thr790A or Met790A, Met793A, Thr854A and Asp855A residues for EGFR_wt_, EGFR_L858R_ and EGFR_L858R/T790M_.

The reaction enthalpy for the ligands/enzyme complexes (∆H_r_) was calculated from enthalpies for formation (∆H_f_) calculated with the semi-empirical PM7 method using the MOPAC2016 program (Stewart Computational Chemistry), according to the following equation:

$$\begin{array}{rcl}{\rm{L}}+{\rm{E}} & \rightleftharpoons  & {\rm{E}}-{\rm{L}}\,{\rm{or}}\,{\rm{L}}-{\rm{X}}+{\rm{E}}\rightleftharpoons {\rm{E}}-{\rm{L}}+{\rm{X}}\\ {{\rm{\Delta }}{\rm{H}}}_{{\rm{r}}} & = & {{\rm{\Delta }}{\rm{H}}}_{{\rm{f}}}({\rm{L}}-{\rm{E}})\mbox{--}{[({\rm{\Delta }}{\rm{H}}}_{{\rm{f}}}({\rm{E}})+{{\rm{\Delta }}{\rm{H}}}_{{\rm{f}}}({\rm{L}})]\,{\rm{or}}\\ {{\rm{\Delta }}{\rm{H}}}_{{\rm{r}}} & = & {{\rm{\Delta }}{\rm{H}}}_{{\rm{f}}}({\rm{L}}-{\rm{E}})+{{\rm{\Delta }}{\rm{H}}}_{{\rm{f}}}({\rm{X}})\\  &  & \mbox{--}\,{[({\rm{\Delta }}{\rm{H}}}_{{\rm{f}}}({\rm{E}})+{{\rm{\Delta }}{\rm{H}}}_{{\rm{f}}}({\rm{LX}})]\end{array}$$

where L is the ligand; X, leaving group; E, the enzyme; and E − L, the covalently bonded complex.

To reduce the computational cost, only the amino acid residues located within 12 Å from Met793 were used and the calculations were performed with the solvent continuum model using the water dielectric constant, 78.4^51^.

## Electronic supplementary material

Supplementary Material
