# Supplementary material for: A novel scaffold for EGFR inhibition: Introducing N-(3-(3-phenylureido)quinoxalin-6-yl) acrylamide derivatives
Source: Sci Rep. 2019 Jan 9;9:14. doi: 10.1038/s41598-018-36846-7 (PMC6327040; doi:10.1038/s41598-018-36846-7)
Supplement: Supplementary file 1 — Supplementary Material [file 41598_2018_36846_MOESM1_ESM.pdf]

## Supplementary Material

***Manuscript Title:*** "A novel scaffold for EGFR inhibition: Introducing N-(3-(3-phenylureido)quinoxalin-6-yl) acrylamide derivatives."

***Authors:*** Daniel Nascimento do Amaral, Jonas Lategahn, Harold Hilarion Fokoue, Eduardo Miguez Bastos da Silva, Carlos Mauricio R. Sant'Anna, Daniel Rauh, Eliezer J. Barreiro, Stefan Laufer*, Lídia Moreira Lima *

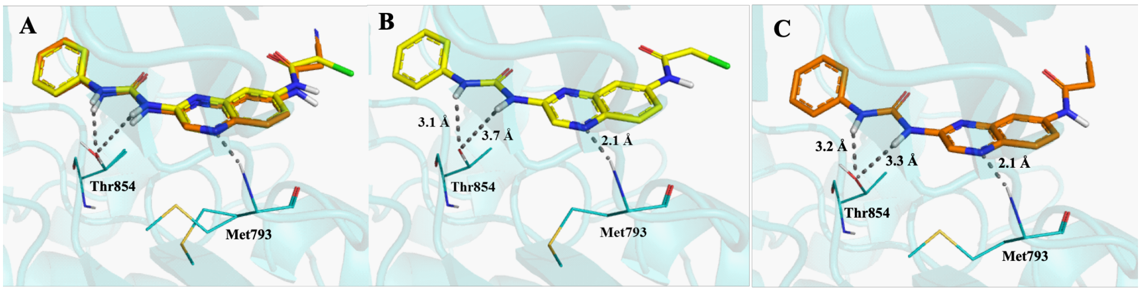


**Figure S1**: Simple docking best poses for compounds **7j** and **7k,** with electrophilic carbon of the α-carbon of carbonyl subunit, in the ATP binding site of EGFRwt. (A) Superposition of **7j** and **7k**; (B) Interaction profile of compound **7j** (*carbon atoms in yellow*); (C) Interaction profile of compound **7i** (*carbon atoms in orange*). *Dashed gray lines*: Hydrogen bonds.

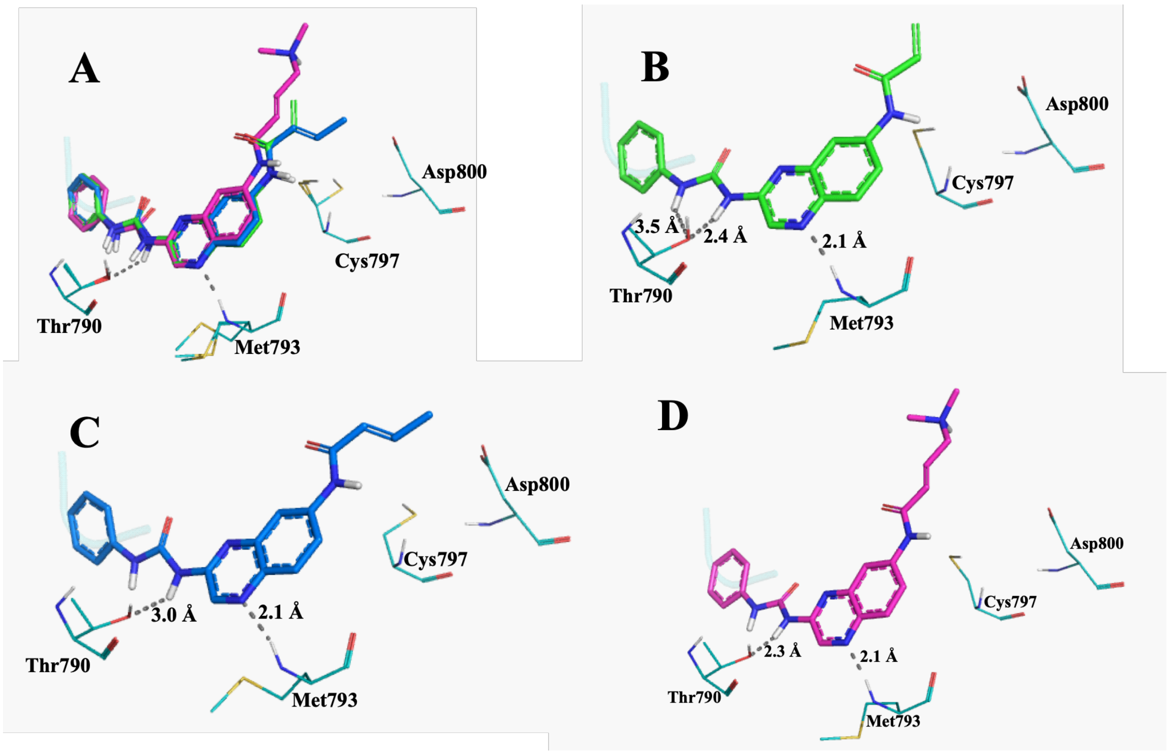


**Figure S2**: Simple docking best poses for compounds **7h, 7i and 7l**, with electrophilic carbon of the enone subunit, in the ATP binding site of $EGFR_{wt}$. (A) Superposition of **7h, 7i and 7l**; (B) Interaction profile of compound **7h** (*carbon atoms in green*); (C) Interaction profile of compound **7i** (*carbon atoms in blue*); (D) Interaction profile of compound **7l** (*carbon atoms in magenta*). *Dashed gray lines*: Hydrogen bonds.

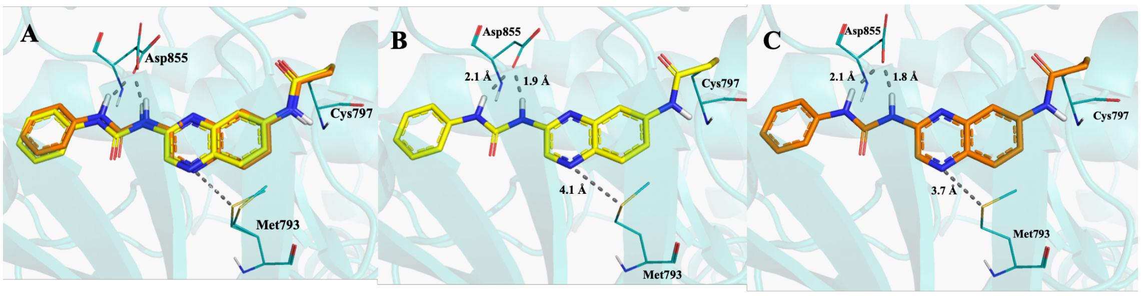


**Figure S3**: Covalent docking best poses for compounds **7j** and **7k,** with electrophilic carbon of the α-carbon of carbonyl subunit, in the ATP binding site of $EGFR_{wt}$. (A) Superposition of **7j** and **7k**; (B) Interaction profile of compound **7j** (*carbon atoms in yellow*); (C) Interaction profile of compound **7k** (*carbon atoms in orange*). *Dashed gray lines*: Hydrogen bonds.

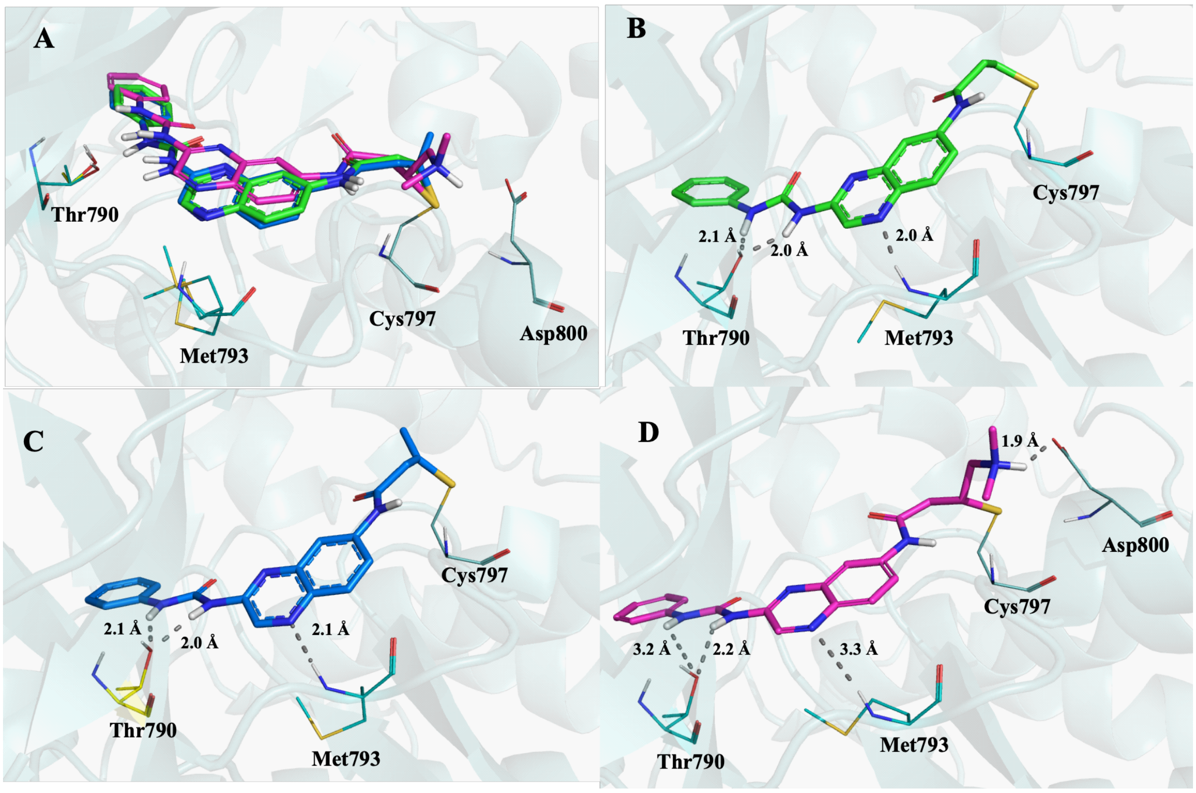


**Figure S4**: Covalent docking best poses for compounds **7h, 7i and 7l**, with electrophilic carbon of the enone subunit, in the ATP binding site of $EGFR_{wt}$. (A) Superposition of **7h, 7i and 7l**; (B) Interaction profile of compound **7h** (*carbon atoms in green*); (C) Interaction profile of compound **7i** (*carbon atoms in blue*); (D) Interaction profile of compound **7l** (*carbon atoms in magenta*). *Dashed gray lines*: Hydrogen bonds.

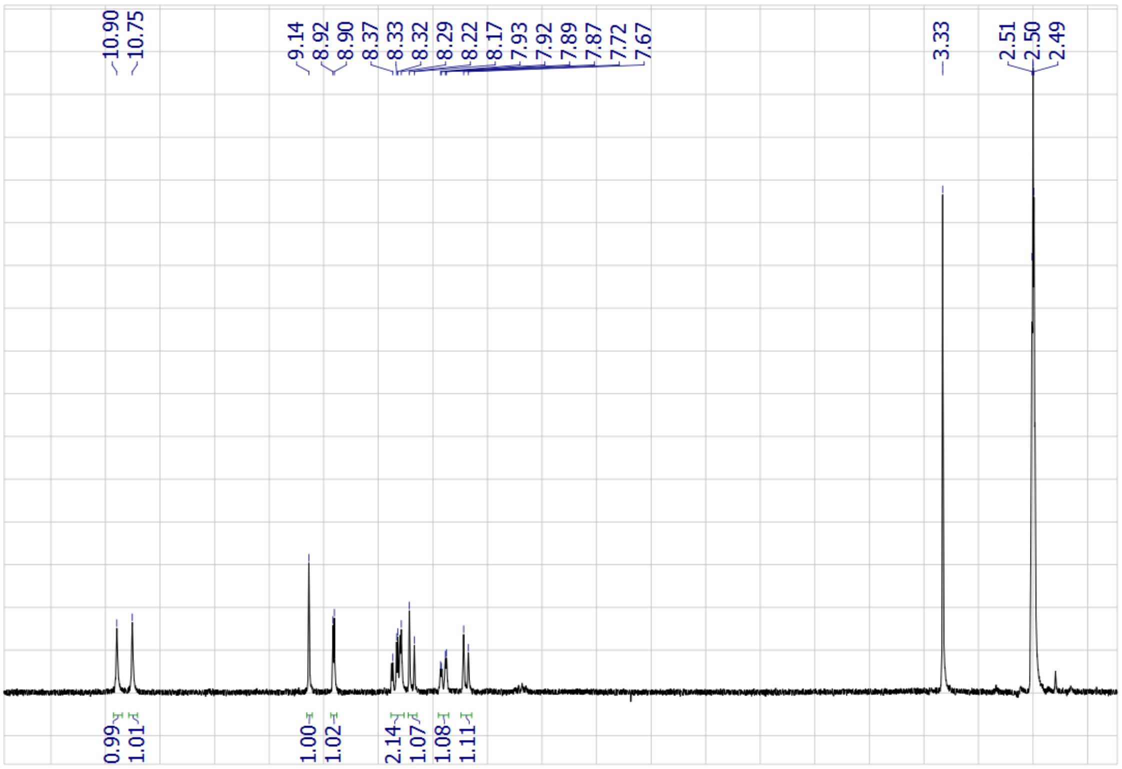


**Figure S5.** $^{1}$H NMR spectrum of compound 1-(7-nitroquinoxalin-2-yl)-3-(4-chloro-3 (trifluormethyl)phenyl)urea **(9a)** (DMSO-$d_6$/200MHz/TMS).

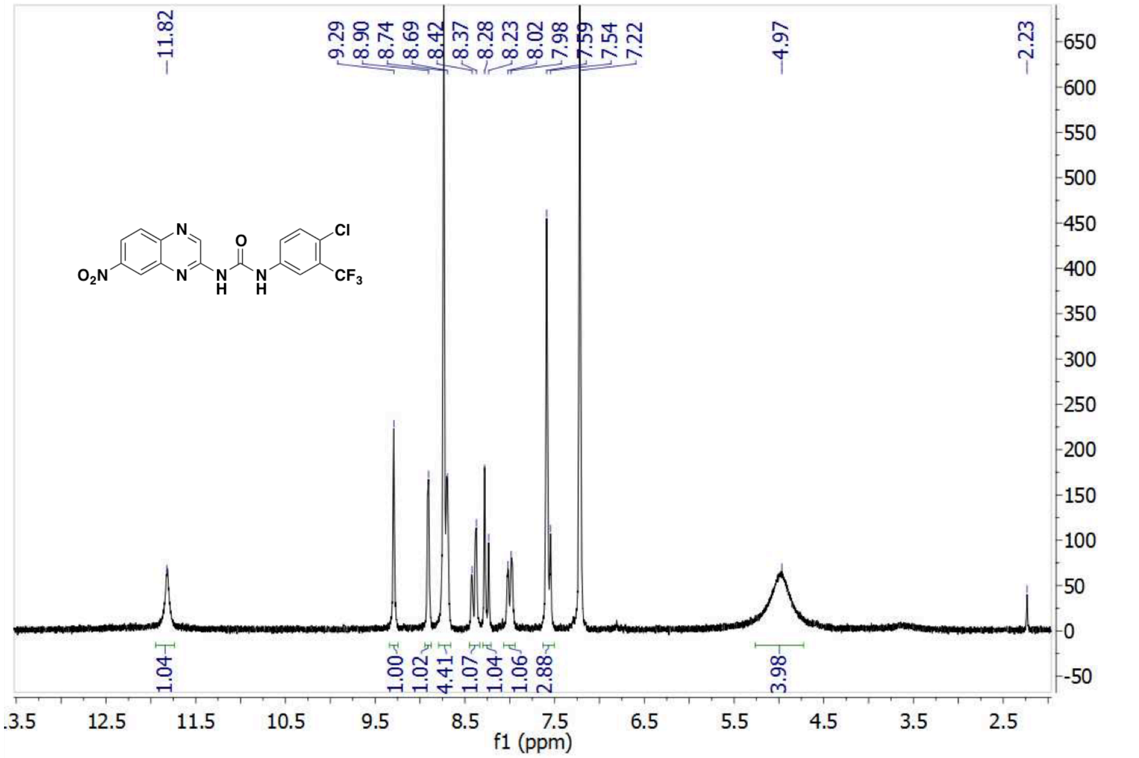


**Figure S6.** $^{1}$H NMR spectrum of compound 1-(7-nitroquinoxalin-2-yl)-3-(4-chloro-3-(trifluormethyl)phenyl)urea **(9a)** (pyridine-$d_5$/200MHz/TMS)

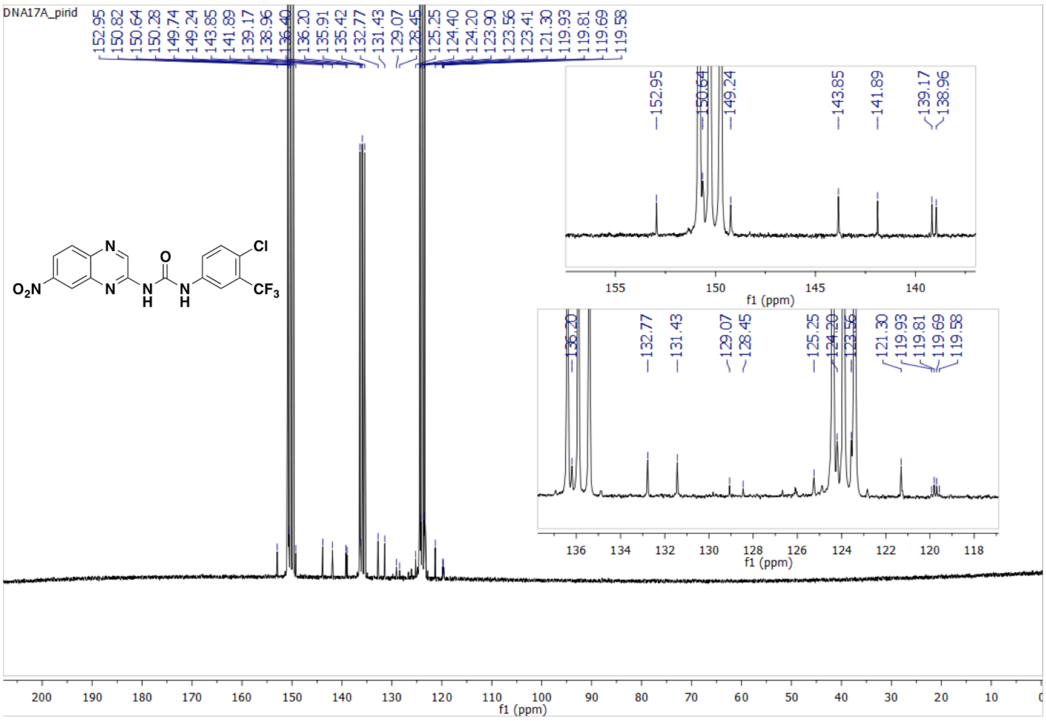


**Figure S7.** $^{13}C$ NMR spectrum of compound 1-(7-nitroquinoxalin-2-yl)-3-(4-chloro-3-(trifluormethyl)phenyl)urea **(9a)** (pyridine-$d_5$/200MHz/TMS)

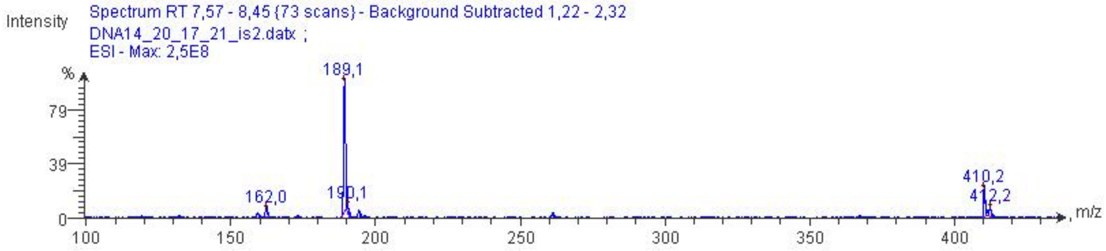


**Figure S8.** Mass spectrometry of compound 1-(7-nitroquinoxalin-2-yl)-3-(4-chloro-3-(trifluormethyl) phenyl)urea **(9a)** in negative mode (ESI-).

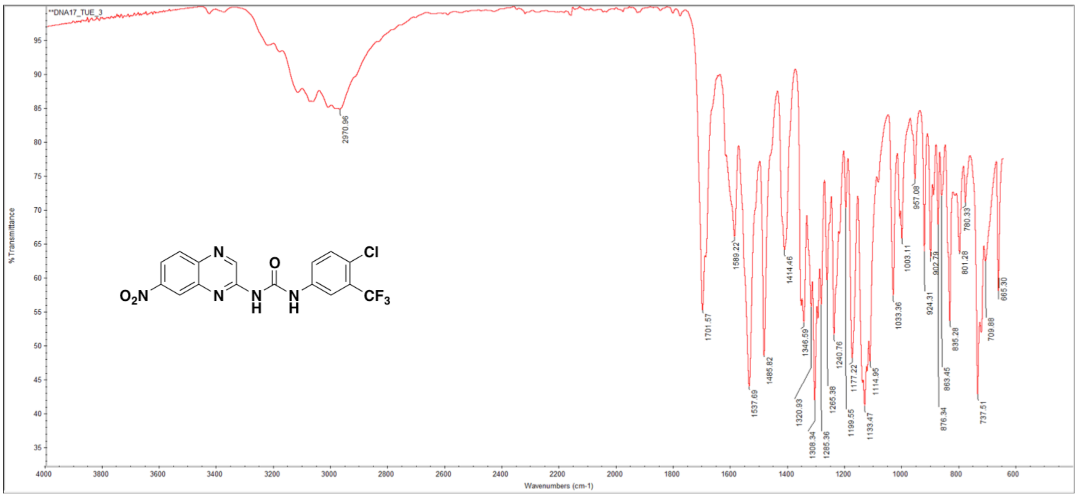


**Figure S9.** Infra red spectrum (ATR-FTIR) of compound 1-(7-nitroquinoxalin-2-yl)-3-(4-chloro-3-(trifluormethyl)phenyl)urea **(9a)** (bands in $cm^{-}$).

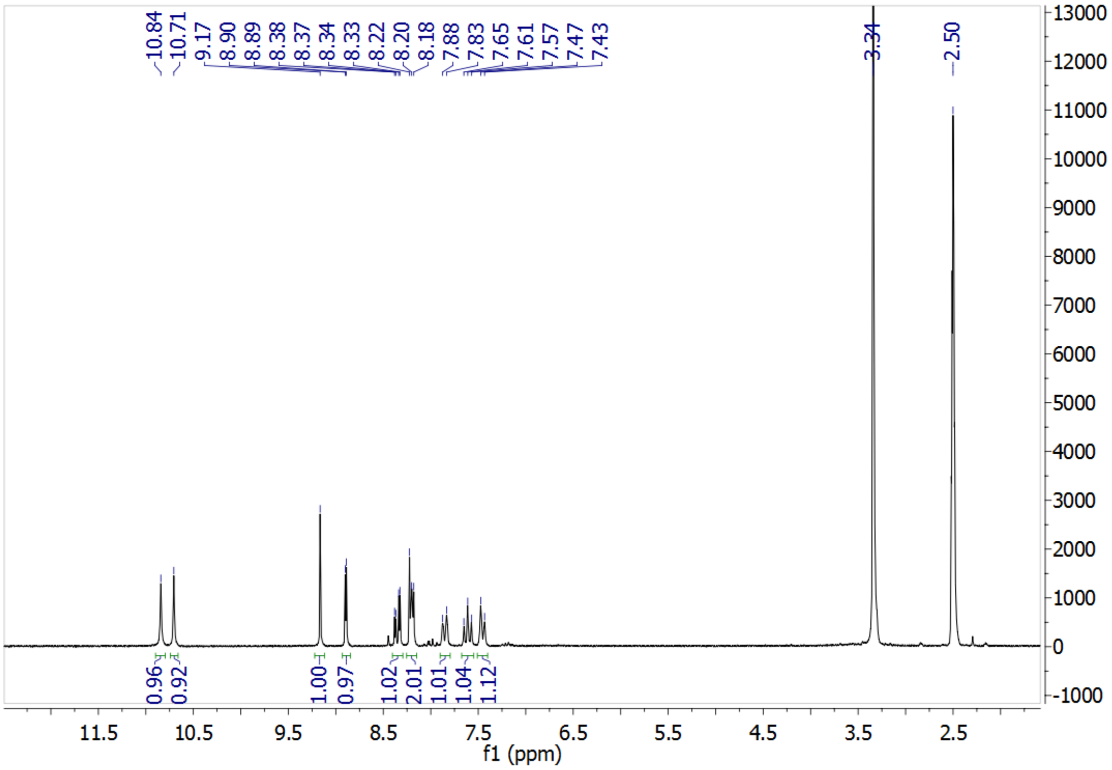


**Figure S10.** $^{1}$H NMR spectrum of compound 1-(7-nitroquinoxalin-2-yl)-3-(3-(trifluormethyl) phenyl)urea **(9b)** (DMSO-$d_6$/200MHz/TMS)

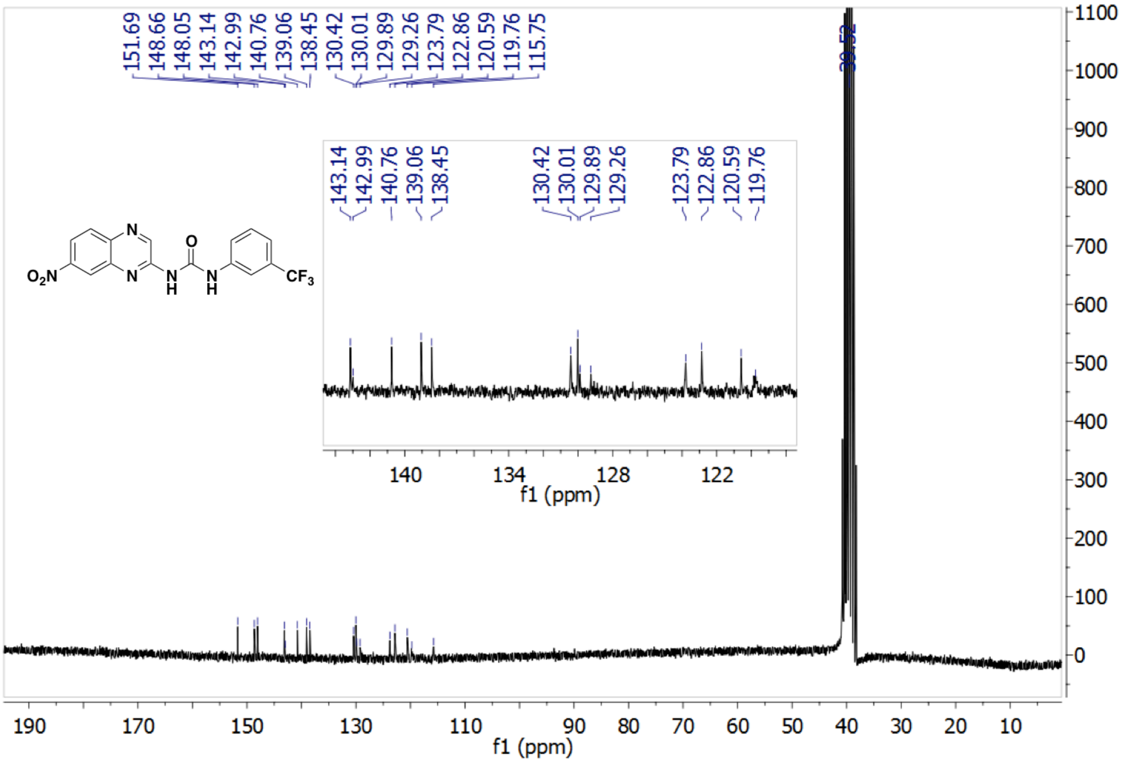


**Figure S11.** $^{13}$C NMR spectrum of compound 1-(7-nitroquinoxalin-2-yl)-3-(3-(trifluormethyl )phenyl) **(9b)** (DMSO-$d_6$/50MHz/TMS)

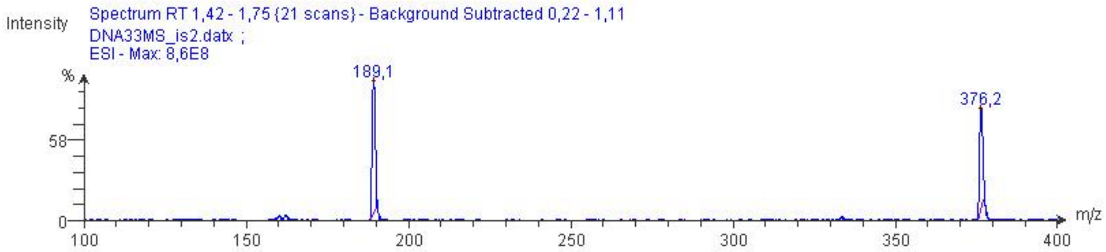


**Figure S12.** Mass spectrometry of compound 1-(7-nitroquinoxalin-2-yl)-3-(3-(trifluormethyl)phenyl) urea **(9b)** in negative mode (ESI-).

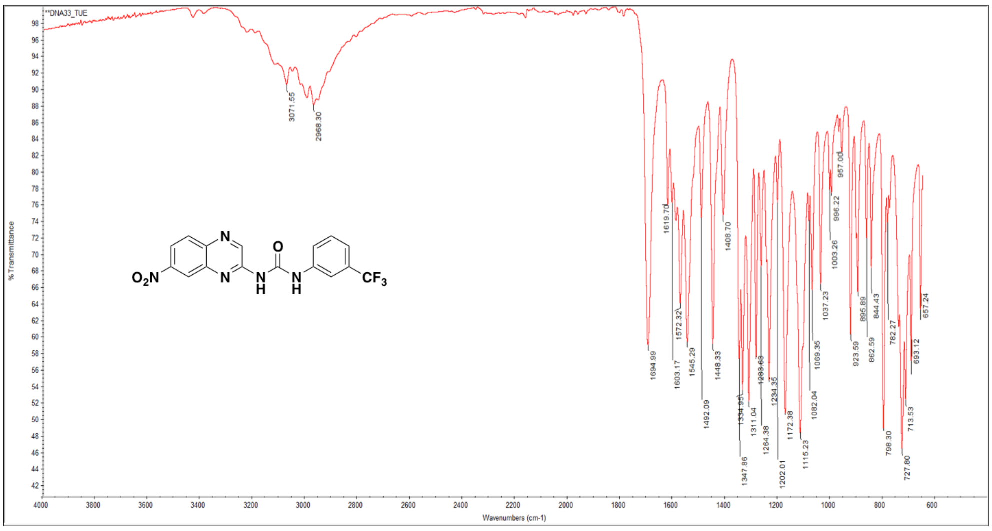


**Figure S13.** Infra red spectrum (ATR-FTIR) of compound 1-(7-nitroquinoxalin-2-yl)-3-(3-(trifluormethyl) phenyl) urea **(9b)** (bands in cm-).

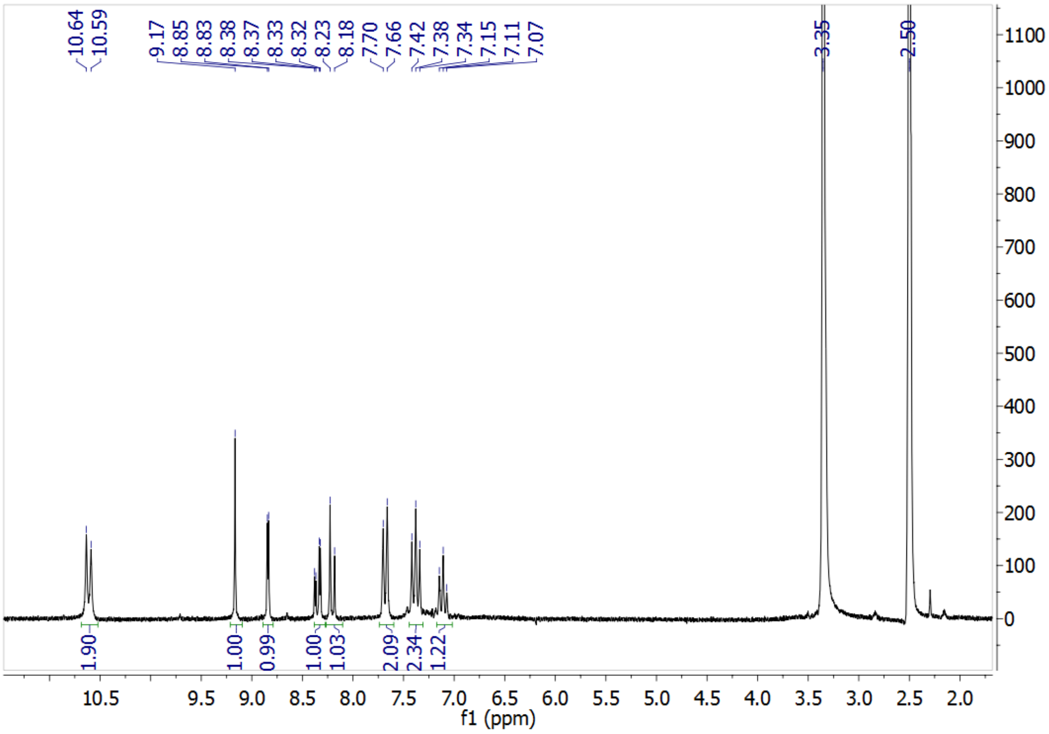


**Figure S14.** $^1$H NMR spectrum of compound 1-(7-nitroquinoxalin-2-yl)-3-phenylurea **(9c)** (DMSO-$d_6$/200MHz/TMS)

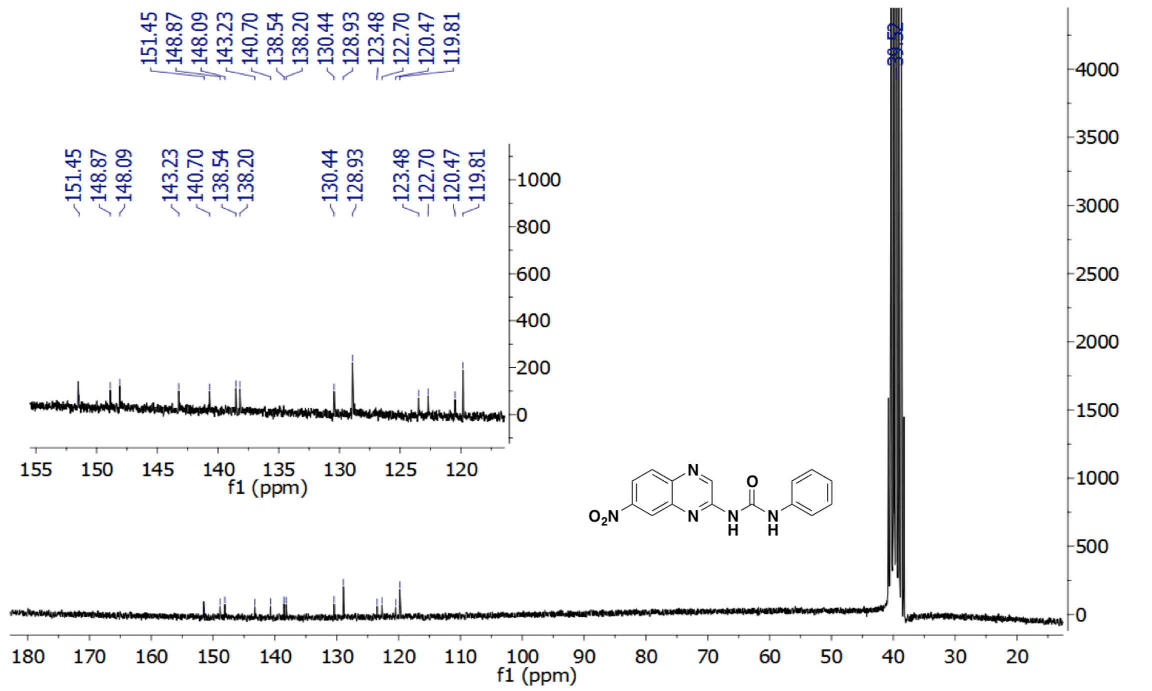


**Figure S15.** $^{13}C$ NMR spectrum of compound 1-(7-nitroquinoxalin-2-yl)-3-phenylurea **(9c)** (DMSO-$d_6$/50MHz/TMS)

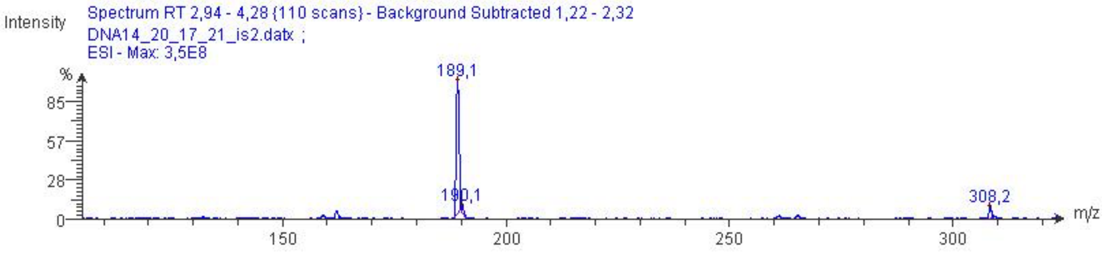


**Figure S16.** Mass spectrometry of compound 1-(7-nitroquinoxalin-2-yl)-3-phenylurea **(9c)** in negative mode (ESI-).

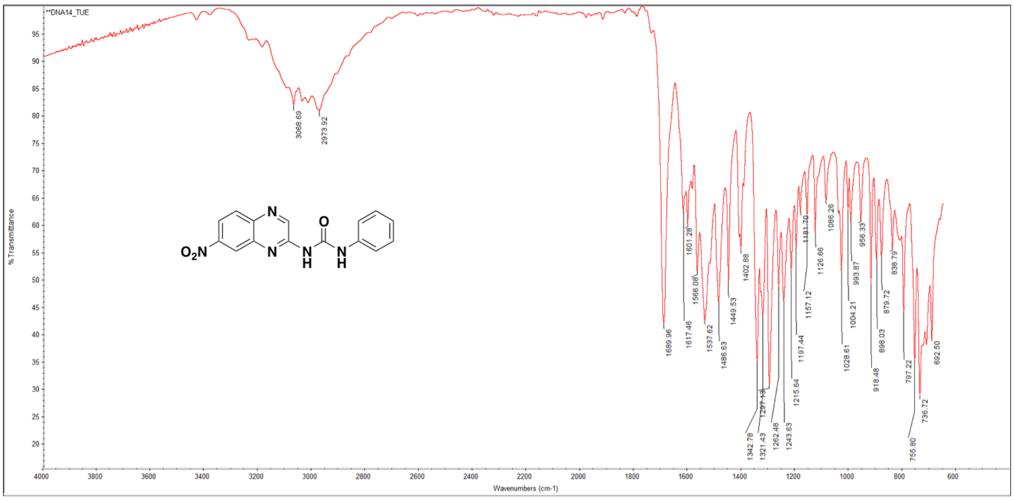


**Figure S17.** Infra red spectrum (ATR-FTIR) of compound 1-(7-nitroquinoxalin-2-yl)-3-phenylurea **(9c)** (bands in $cm^{-}$).

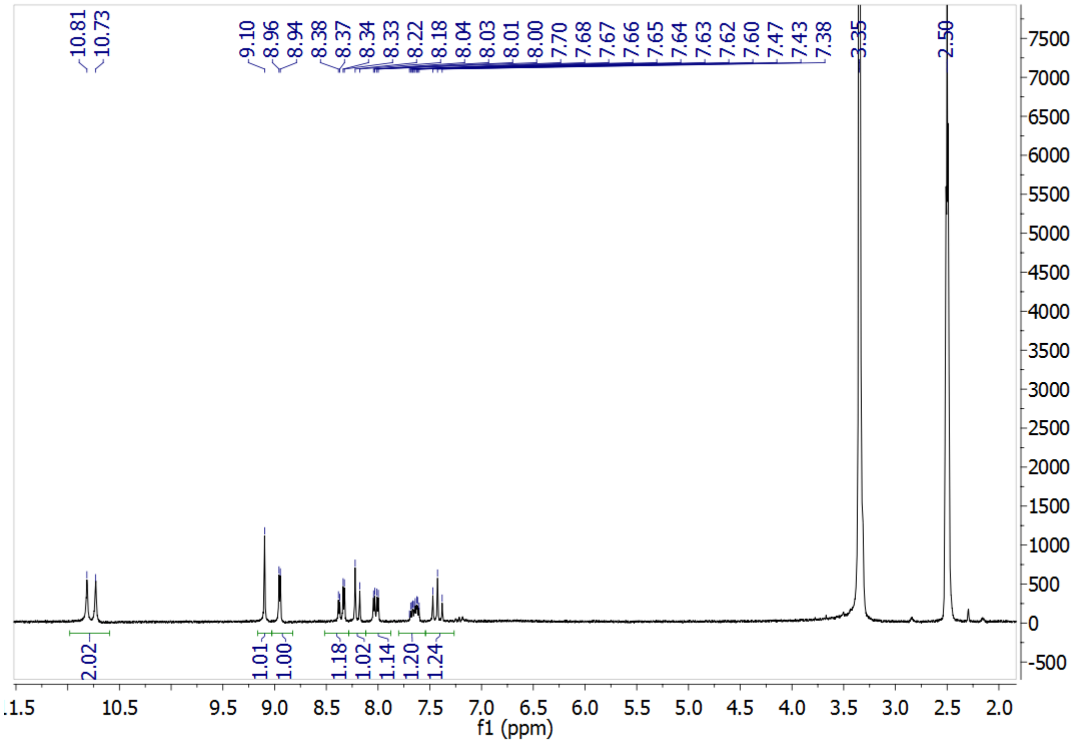

**Figure S18.** $^{1}H$ NMR spectrum of compound 1-(3-chloro-4-fluorophenyl)-3-(7-nitroquinoxalin-2-yl)urea **(9d)** (DMSO-$d_6$/200MHz/TMS)

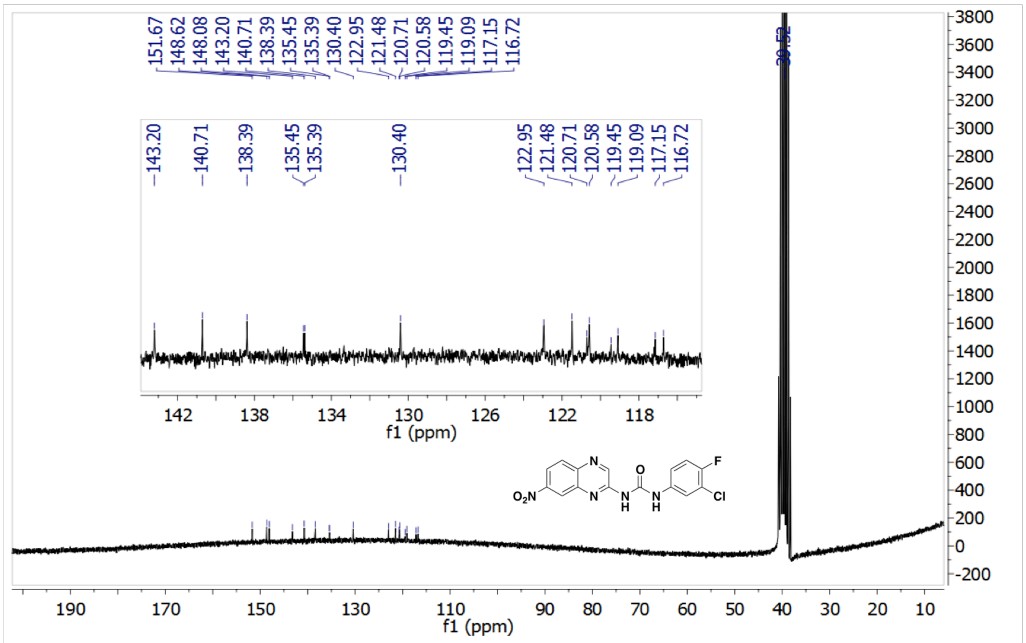

**Figure S19.** $^{13}C$ NMR spectrum of compound 1-(3-chloro-4-fluorophenyl)-3-(7-nitroquinoxalin-2-yl)urea **(9d)** (DMSO-$d_6$/50MHz/TMS)

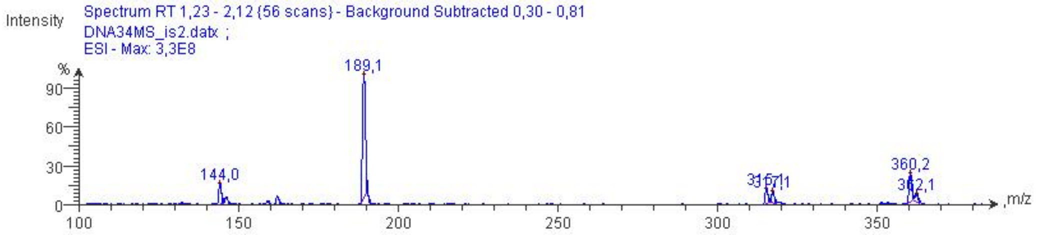


**Figure S20.** Mass spectrometry of compound 1-(3-chloro-4-fluorophenyl)-3-(7-nitroquinoxalin-2-yl)urea **(9d)** in negative mode (ESI-).

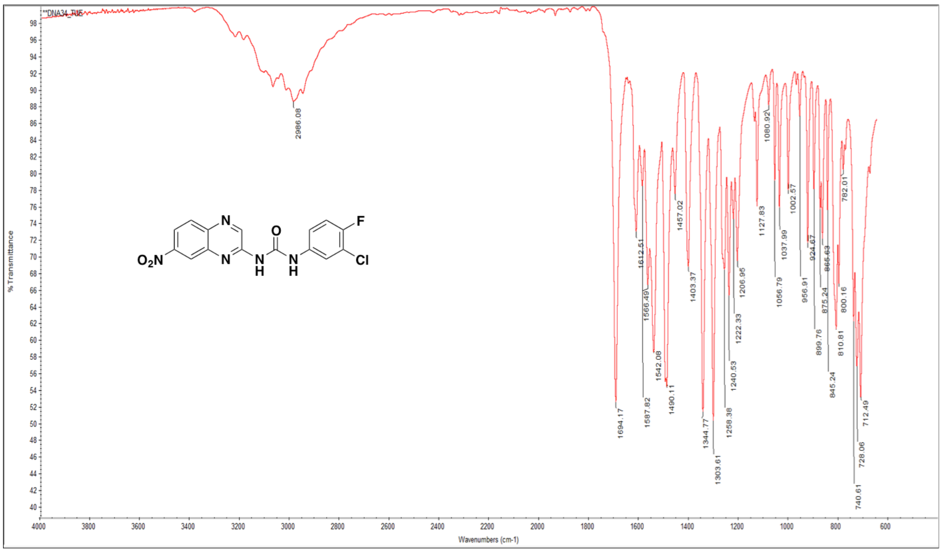


**Figure S21.** Infra red spectrum (ATR-FTIR) of compound 1-(3-chloro-4-fluorophenyl)-3-(7-nitroquinoxalin-2-yl)urea **(9d)** (bands in cm-).

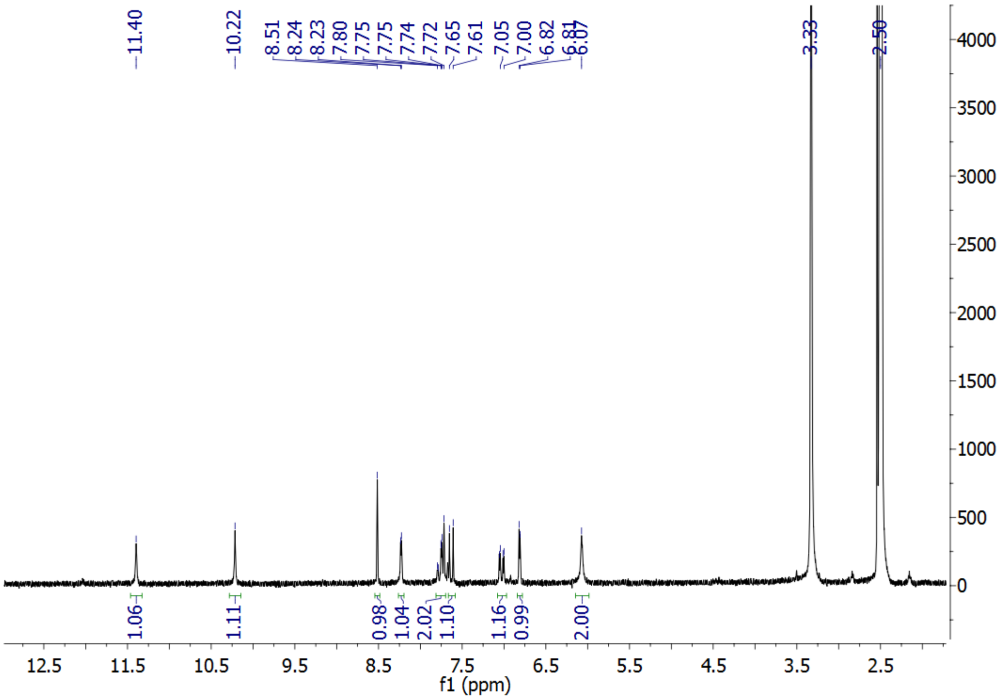


**Figure S22.** $^{1}H$ NMR spectrum do 1-(7-aminoquinoxalin-2-yl)-3-(4-chloro-3-(trifluoromethyl)phenyl)urea **(10a)** (DMSO-$d_6$/200MHz/TMS)

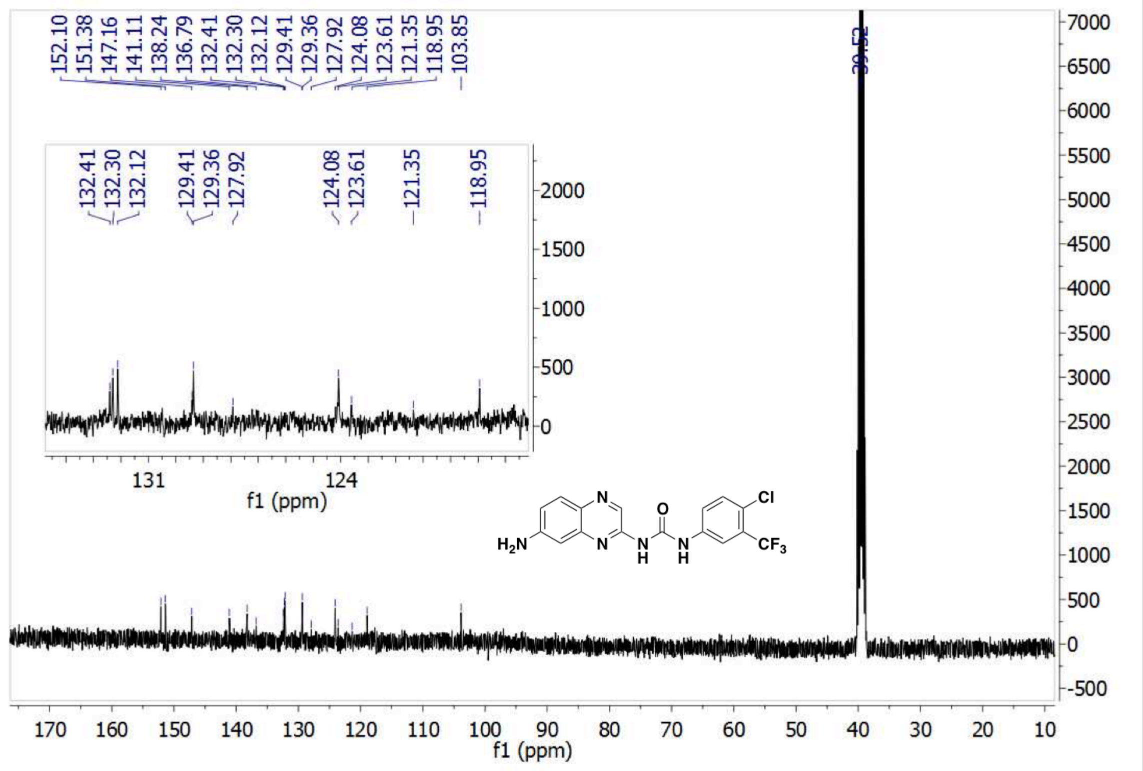


**Figure S23.** $^{13}C$ NMR spectrum of compound 1-(7-aminoquinoxalin-2-yl)-3-(4-chloro-3-(trifluoromethyl) phenyl)urea **(10a)** (DMSO-$d_6$/100MHz/TMS)

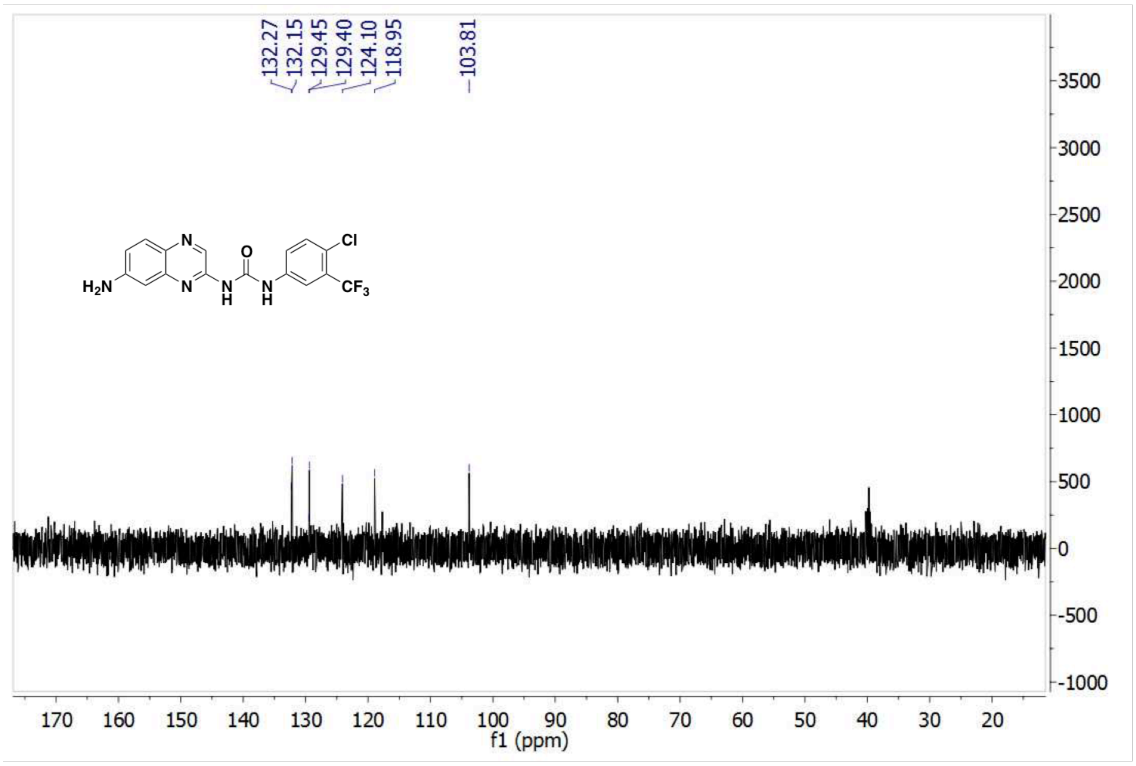


**Figure S24.** Espectro de DEPT 135 of compound 1-(7-aminoquinoxalin-2-yl)-3-(4-chloro-3-(trifluoromethyl) phenyl)urea **(10a)** (DMSO-$d_6$/100MHz/TMS)

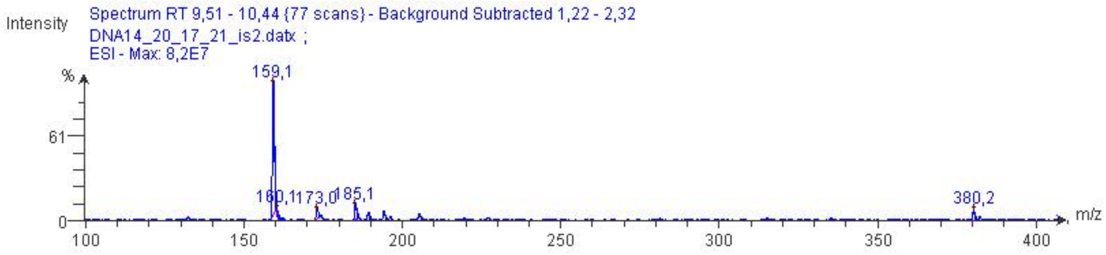


**Figure S25.** Mass spectrometry of compound 1-(7-aminoquinoxalin-2-yl)-3-(4-chloro-3-(trifluormethyl) phenyl) urea **(10a)** in negative mode (ESI-).

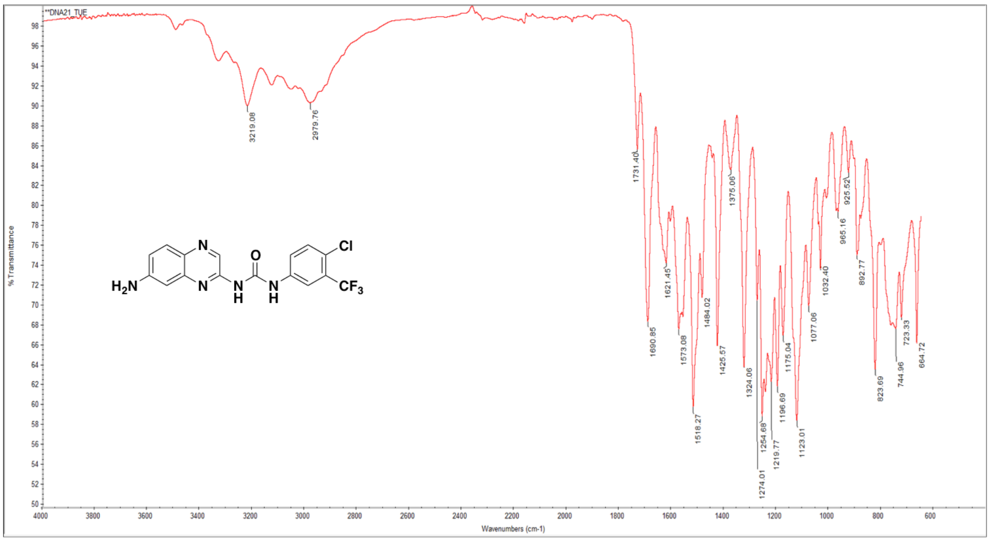


**Figure S26.** Infra red spectrum (ATR-FTIR) of compound 1-(7-aminoquinoxalin-2-yl)-3-(4-chloro-3-(trifluormethyl) phenyl) urea **(10a)** (bands in cm$^{-}$).

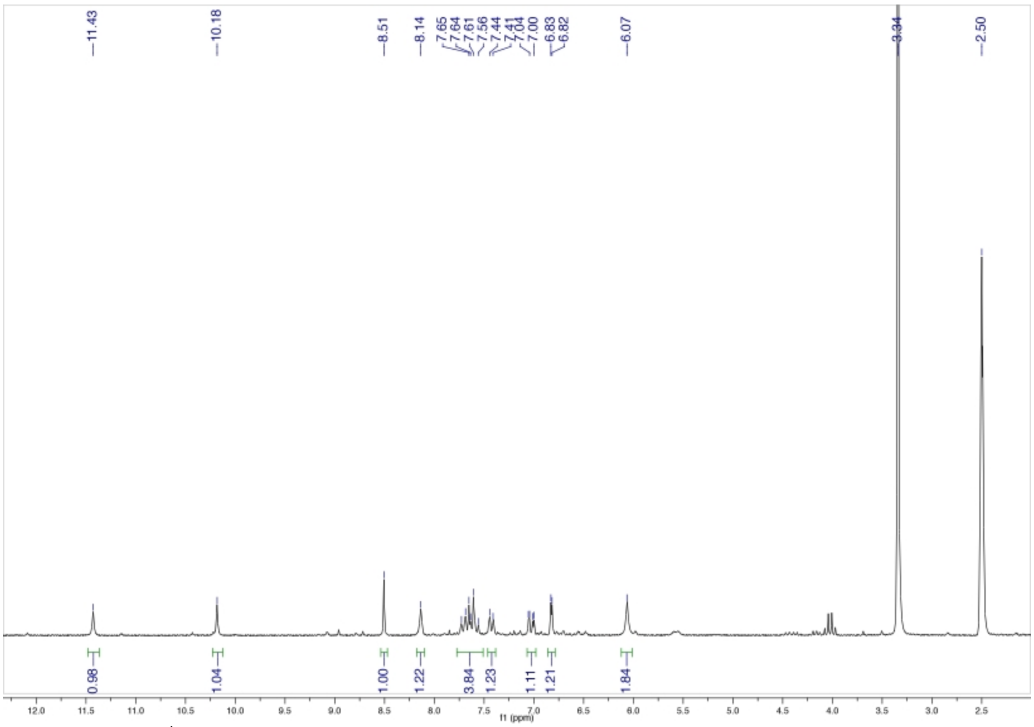


**Figure S27.** $^{1}$H NMR spectrum of compound 1-(7-aminoquinoxalin-2-yl)-3-(3-(trifluormethyl)phenyl) urea **(10b)** (DMSO-d$_6$/200MHz/TMS)

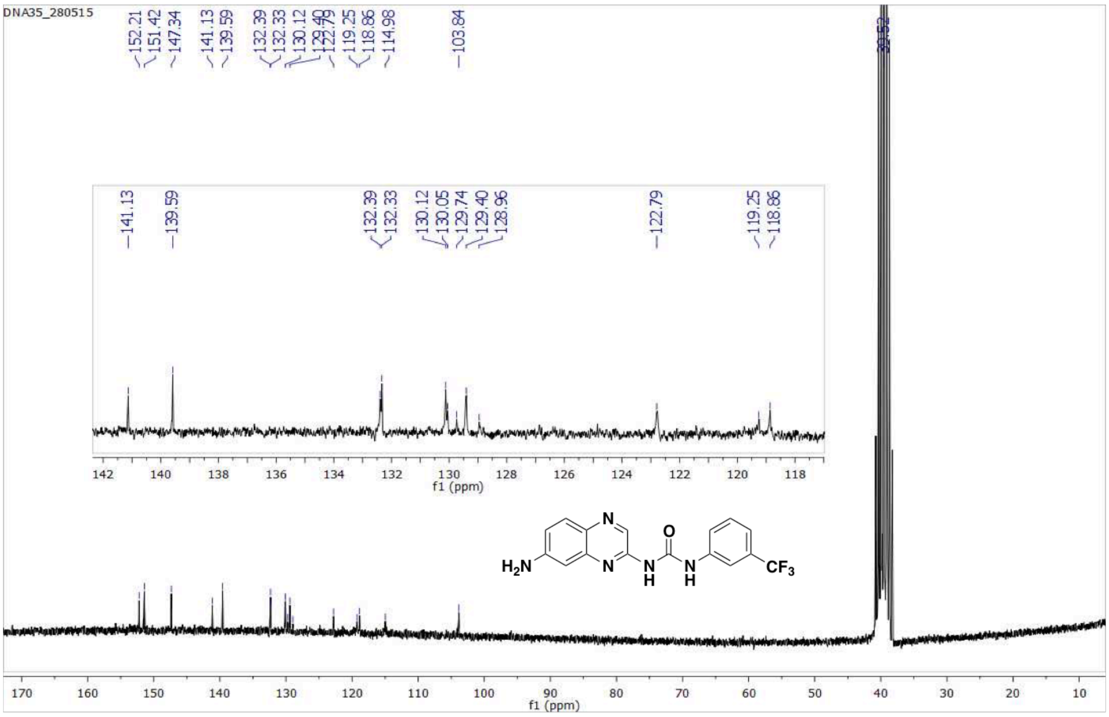


**Figure S28.** $^{13}C$ NMR spectrum of compound 1-(7-aminoquinoxalin-2-yl)-3-(3-(trifluormethyl)phenyl) urea **(10b)** (DMSO-$d_6$/50MHz/TMS)

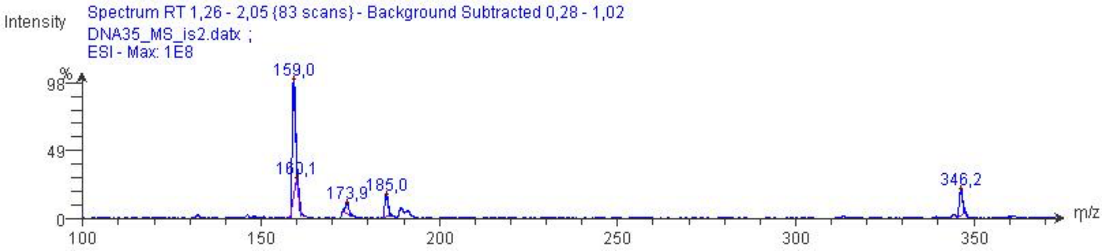


**Figure S29.** Mass spectrometry of compound 1-(7-aminoquinoxalin-2-yl)-3-(3-(trifluormethyl)phenyl) urea **(10b)** in negative mode (ESI-).

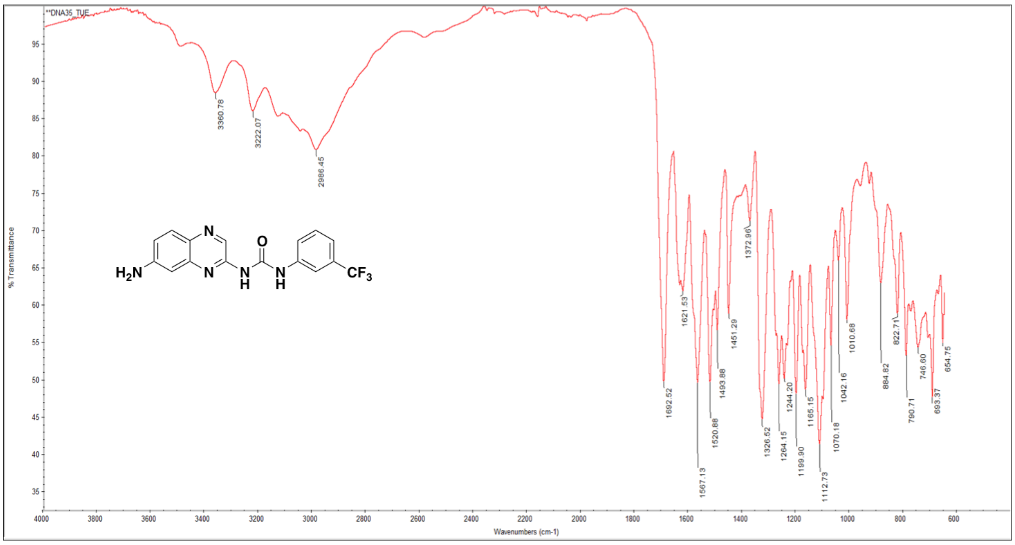


**Figure S30.** Infra red spectrum (ATR-FTIR) of compound 1-(7-aminoquinoxalin-2-yl)-3-(3-(trifluormethyl)phenyl) urea **(10b)** (bands in $cm^{-}$).

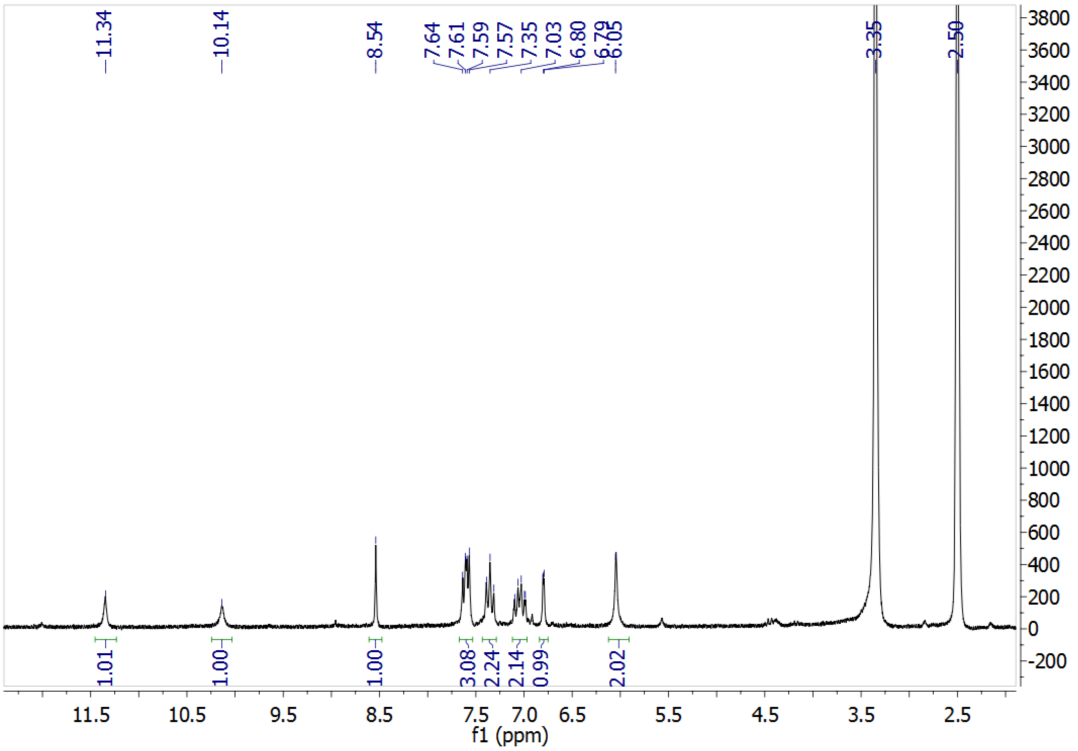


**Figure S31.** $^{1}$H NMR spectrum of compound 1-(7-aminoquinoxalin-2-yl)-3-phenylurea **(10c)** (DMSO-$d_6$/200MHz/TMS)

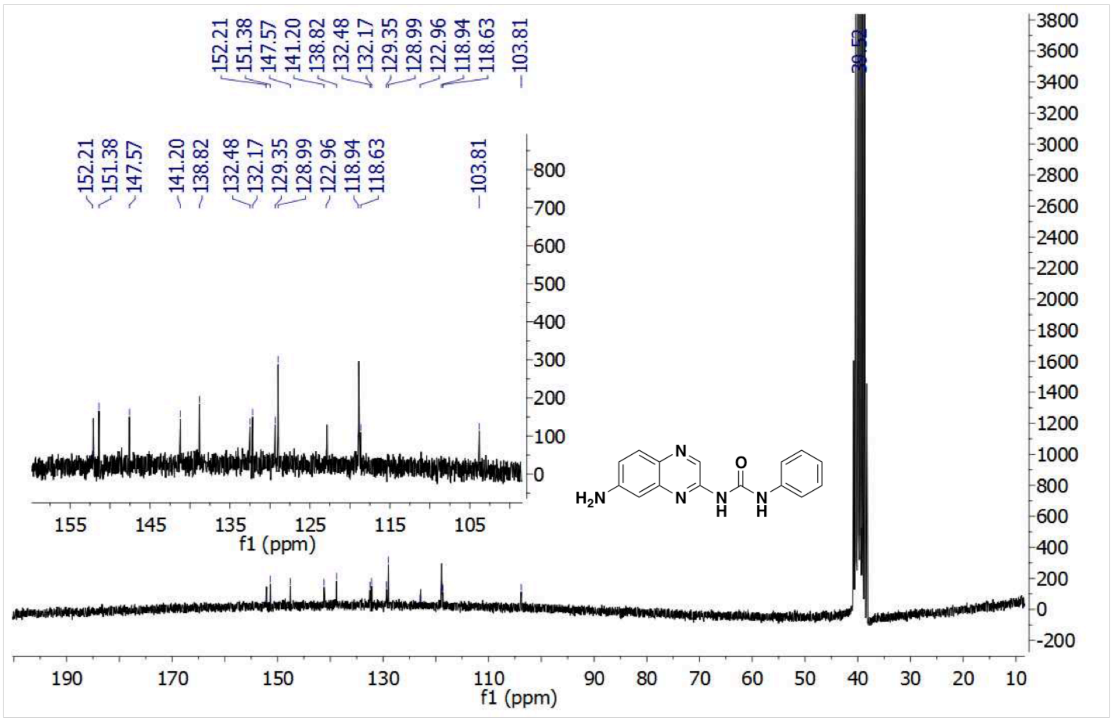


**Figure S32.** $^{13}$C NMR spectrum of compound 1-(7-aminoquinoxalin-2-yl)-3-phenylurea **(10c)** (DMSO-$d_6$/50MHz/TMS)

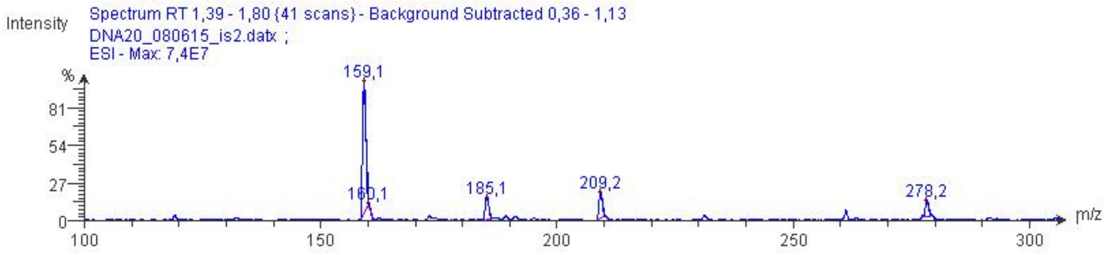


**Figure S33.** Mass spectrometry of compound 1-(7-aminoquinoxalin-2-yl)-3-phenylurea **(10c)** in negative mode (ESI-).

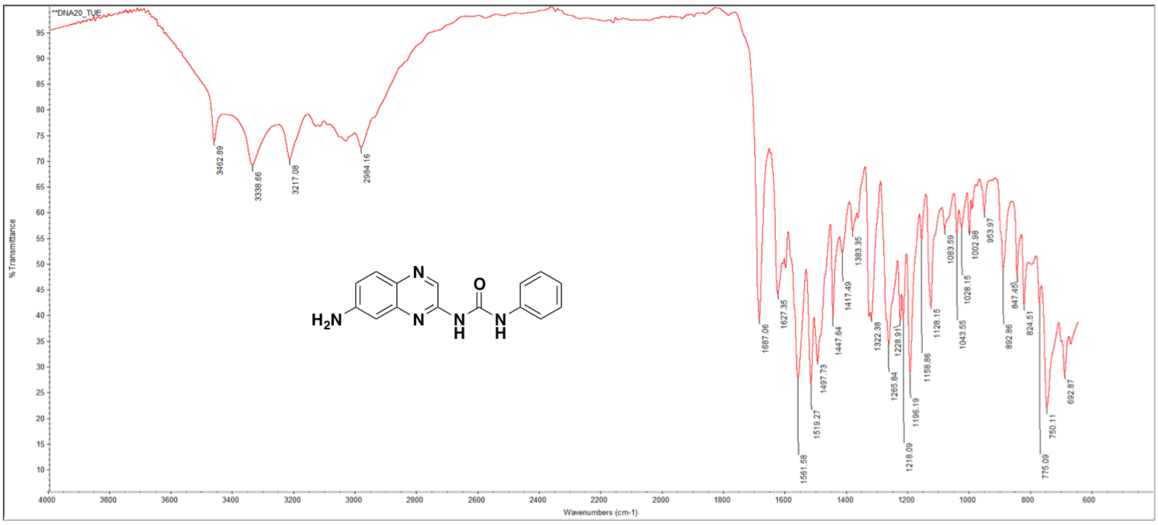


**Figure S34.** Infra red spectrum (ATR-FTIR) of compound 1-(7-aminoquinoxalin-2-yl)-3-phenylurea **(10c)** (bands in $cm^{-}$)

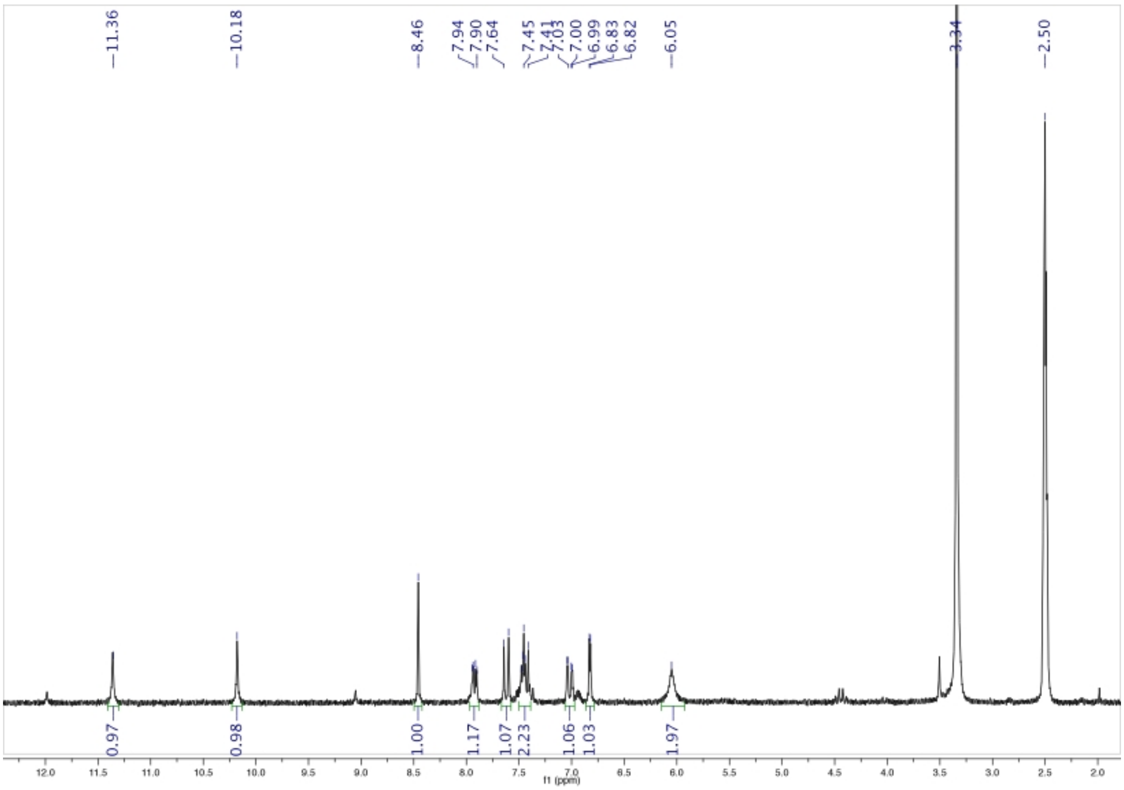


**Figure S35.** $^{1}$H NMR spectrum of compound 1 - (7-aminoquinoxalin-2-yl)-3-(3-chloro-4-fluorophenyl) urea **(10d)** (DMSO-$d_6$/200MHz/TMS)

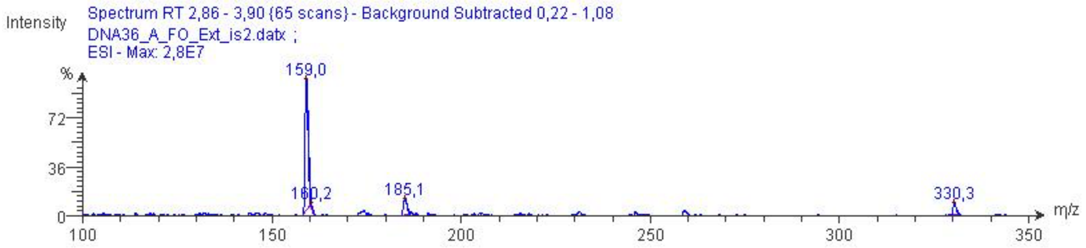


**Figure S36.** Mass spectrometry of compound 1 - (7-aminoquinoxalin-2-yl)-3-(3-chloro-4-fluorophenyl) urea **(10d)** in negative mode (ESI-).

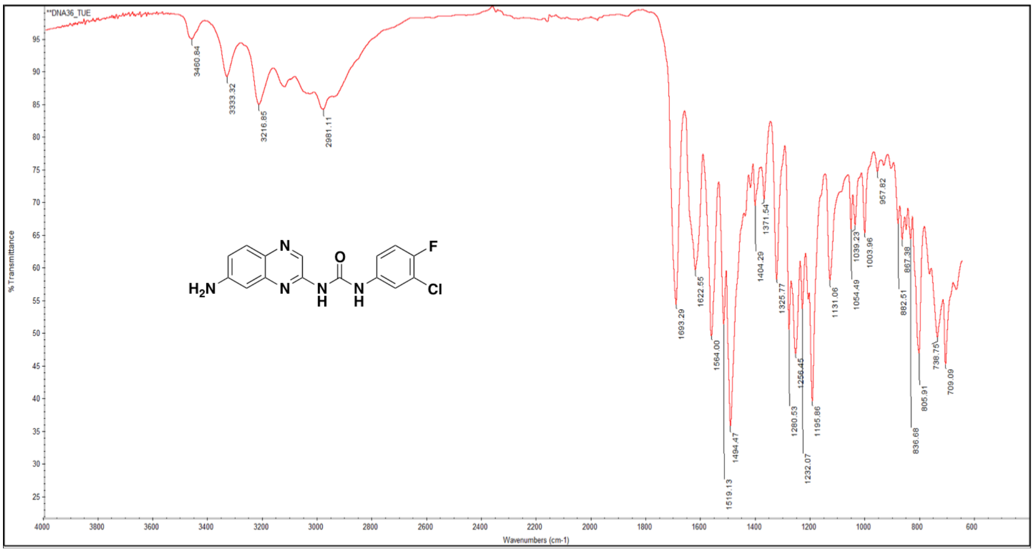


**Figure S37.** Infra red spectrum (ATR-FTIR) of compound 1 - (7-aminoquinoxalin-2-yl)-3-(3-chloro-4-fluorophenyl) urea **(10d)** (bands in cm$^{-}$).

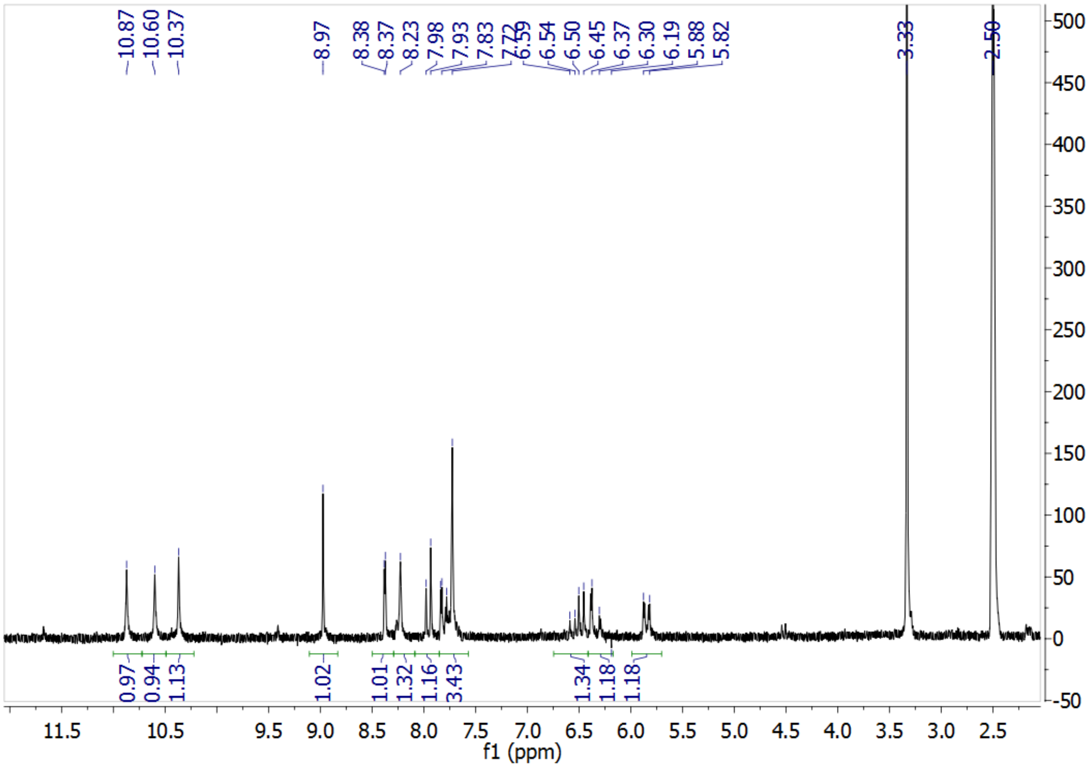

**Figure S38.** $^{1}H$ NMR spectrum of compound *N*-(3-(3-(4-chloro-3-(trifluoromethyl)phenyl) ureido)quinoxalin-6-yl)acrylamide **(7a)** (DMSO-$d_6$/200MHz/TMS)

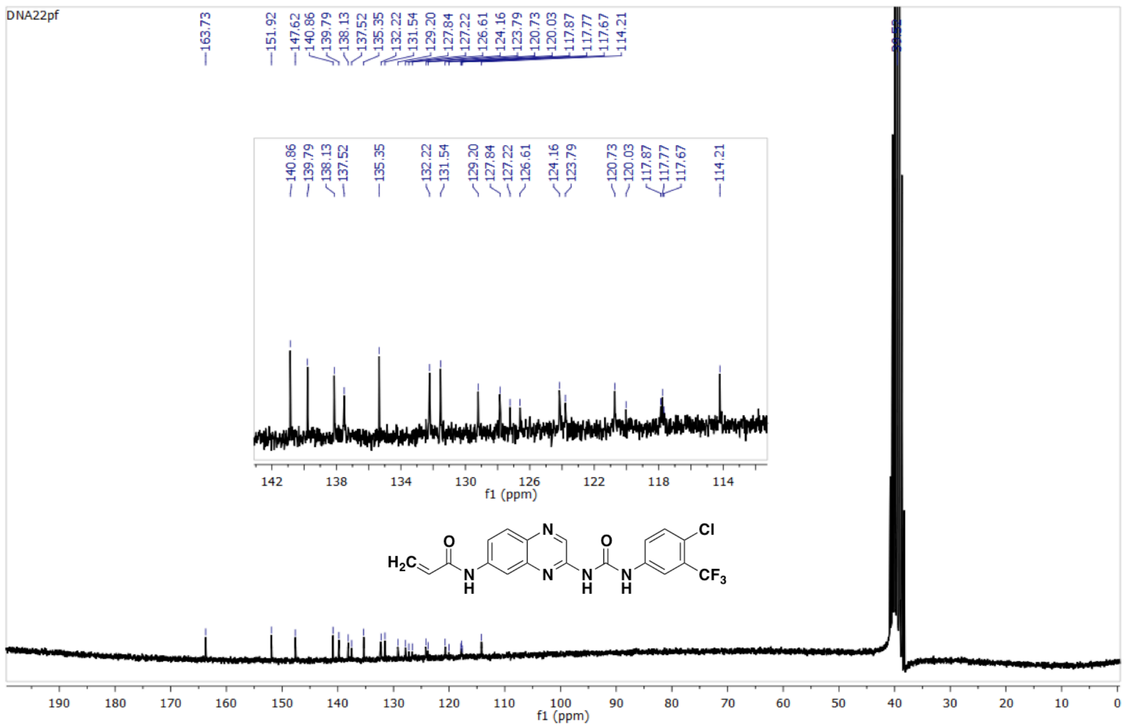


**Figure S39.** $^{13}C$ NMR spectrum of compound *N*-(3-(3-(4-chloro-3-(trifluoromethyl)phenyl) ureido)quinoxalin-6-yl)acrylamide **(7a)** (DMSO-$d_6$/50MHz/TMS)

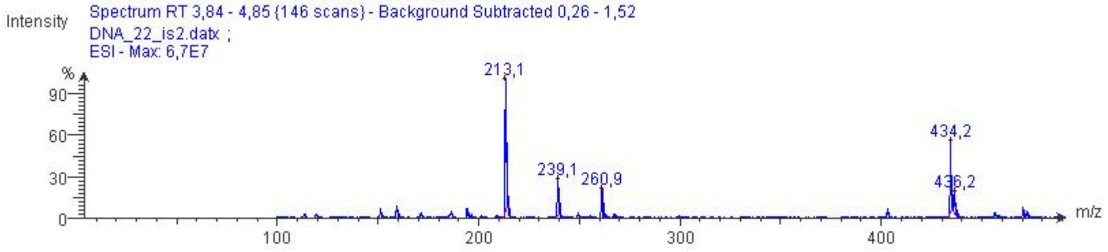


**Figure S40.** Mass spectrometry of compound *N*-(3-(3-(4-chloro-3-(trifluoromethyl)phenyl)ureido) quinoxalin-6-yl) acrylamide **(7a)** in negative mode (ESI-).

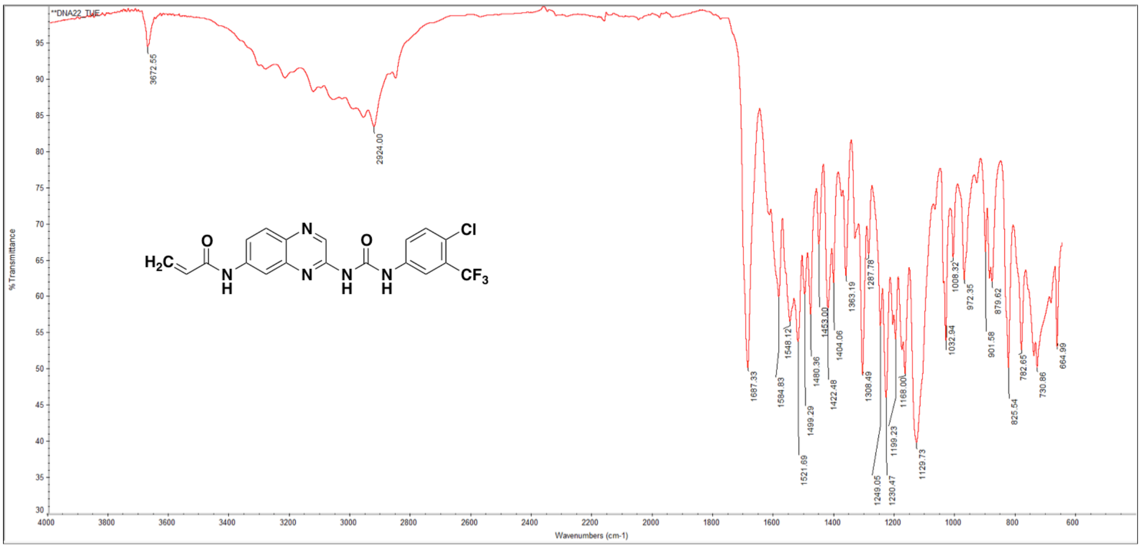

**Figure S41.** Infra red spectrum (ATR-FTIR) of compound *N*-(3-(3-(4-chloro-3-(trifluoromethyl) phenyl)ureido) quinoxalin-6-yl) acrylamide **(7a)** (bands in cm⁻).

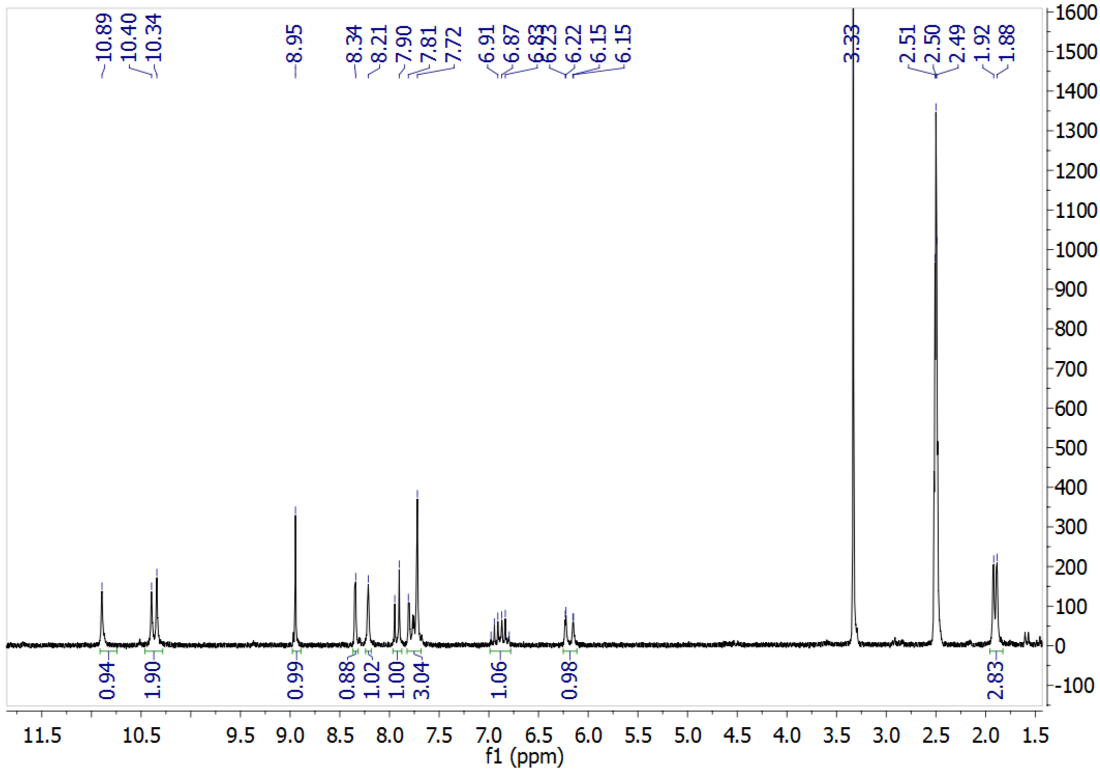


**Figure S42.** $^{1}H$ NMR spectrum of compound (*E*)-*N*-(3-(3-(4-chloro-3-(trifluoromethyl) phenyl)ureido)quinoxalin-6-yl)but-2-enamide **(7b)** (DMSO-$d_6$/200MHz/TMS)

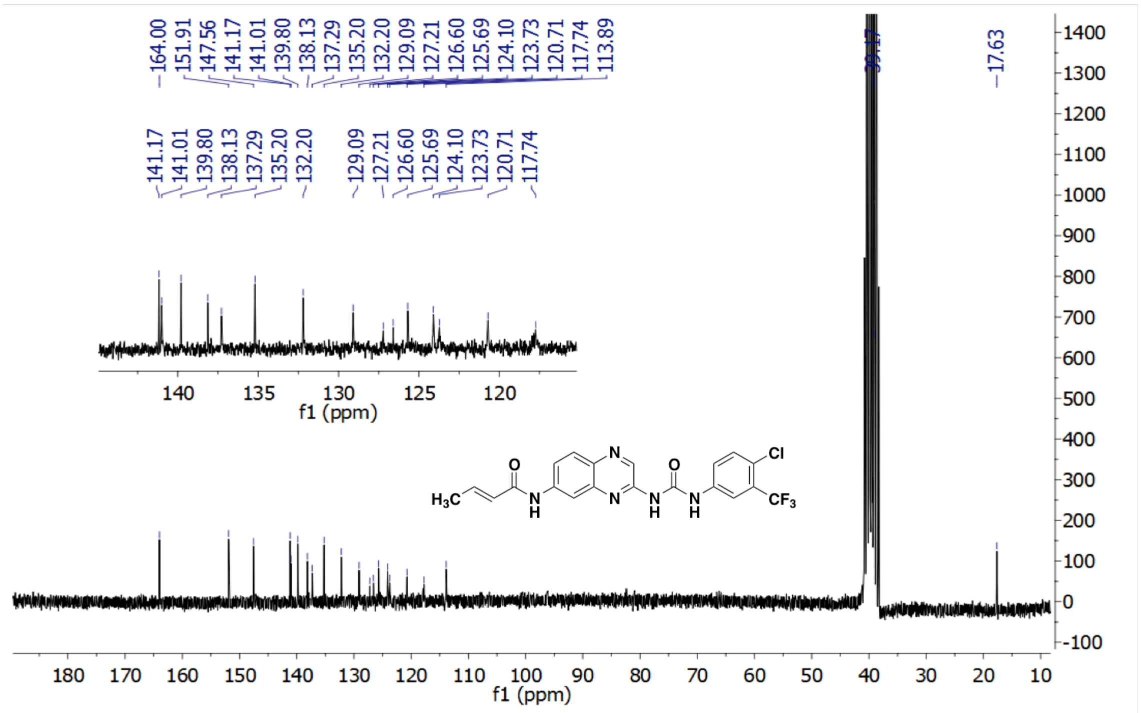


**Figure S43.** $^{13}C$ NMR spectrum of compound (*E*)-*N*-(3-(3-(4-chloro-3-(trifluoromethyl)phenyl)ureido)quinoxalin-6-yl)but-2-enamide **(7b)** (DMSO-$d_6$/50MHz/TMS).

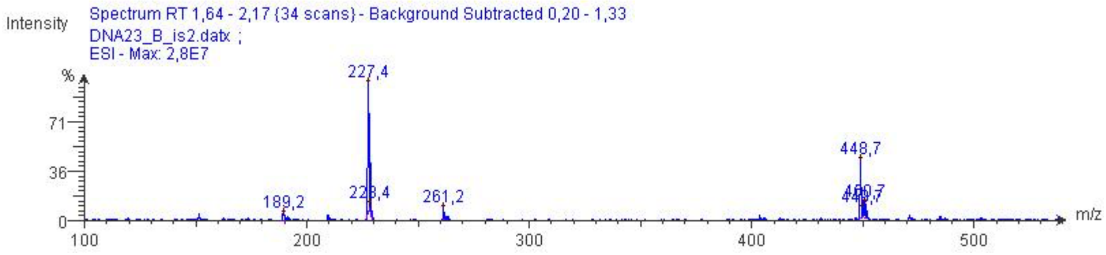


**Figure S44.** Mass spectrometry of compound (*E*)-*N*-(3-(3-(4-chloro-3-(trifluoromethyl)phenyl)ureido) quinoxalin-6-yl)but-2-enamide **(7b)** in negative mode (ESI-).

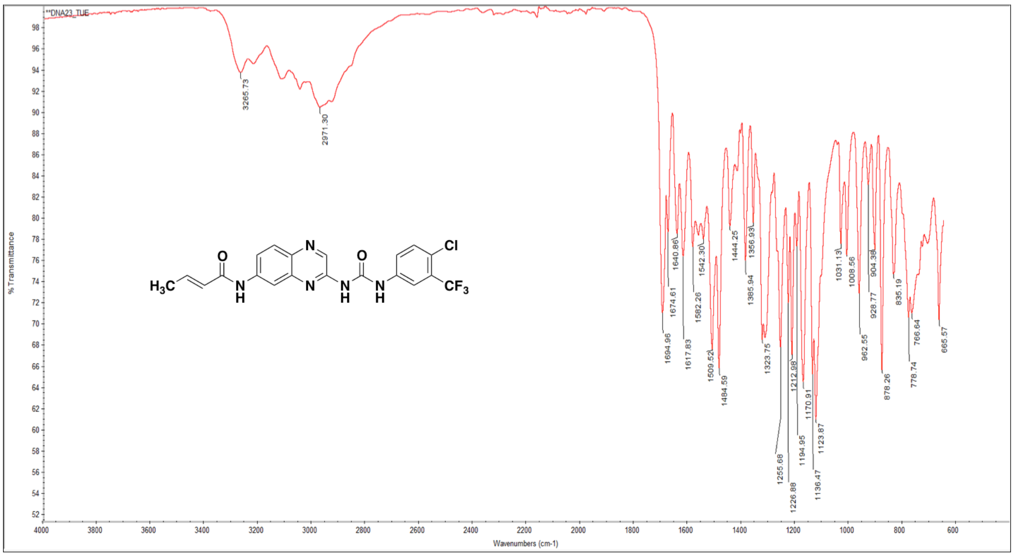


**Figure S45.** Infra red spectrum (ATR-FTIR) of compound (*E*)-*N*-(3-(3-(4-chloro-3-(trifluoromethyl)phenyl)ureido) quinoxalin-6-yl)but-2-enamide **(7b)** (bands in $cm^{-}$).

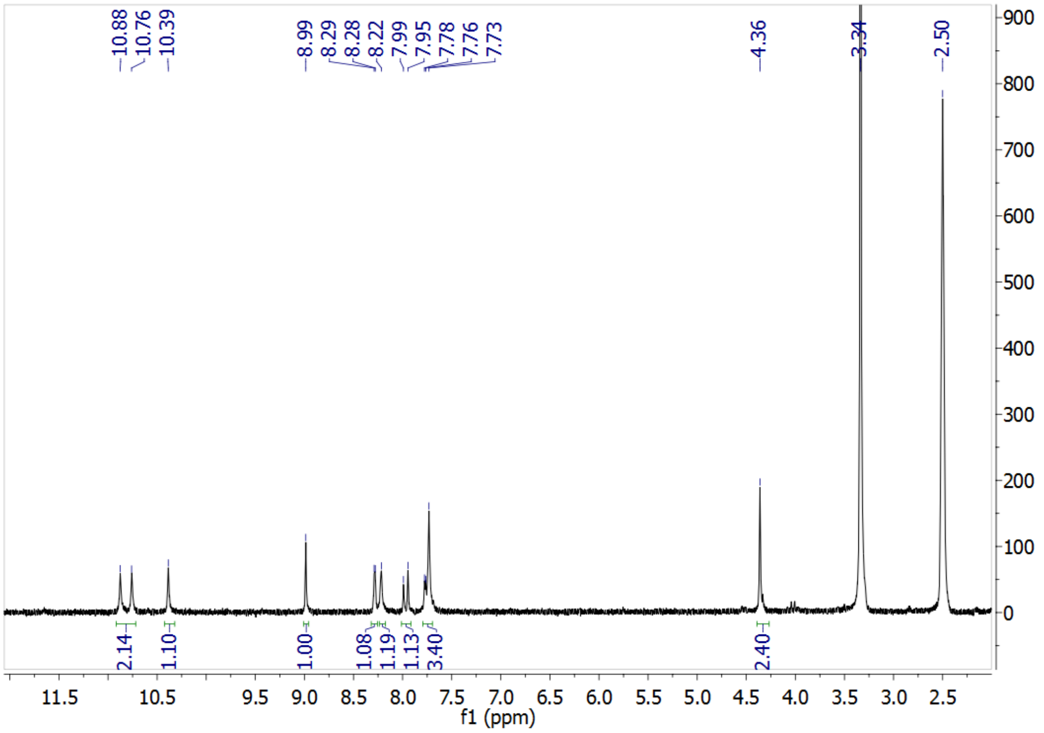


**Figure S46.** $^{1}$H NMR spectrum of compound 2-chloro-*N*-(3-(3-(4-chloro-3-(trifluoromethyl) phenyl)ureido) quinoxalin-6-yl)acetamide **(7c)** (DMSO-d6/200MHz/TMS)

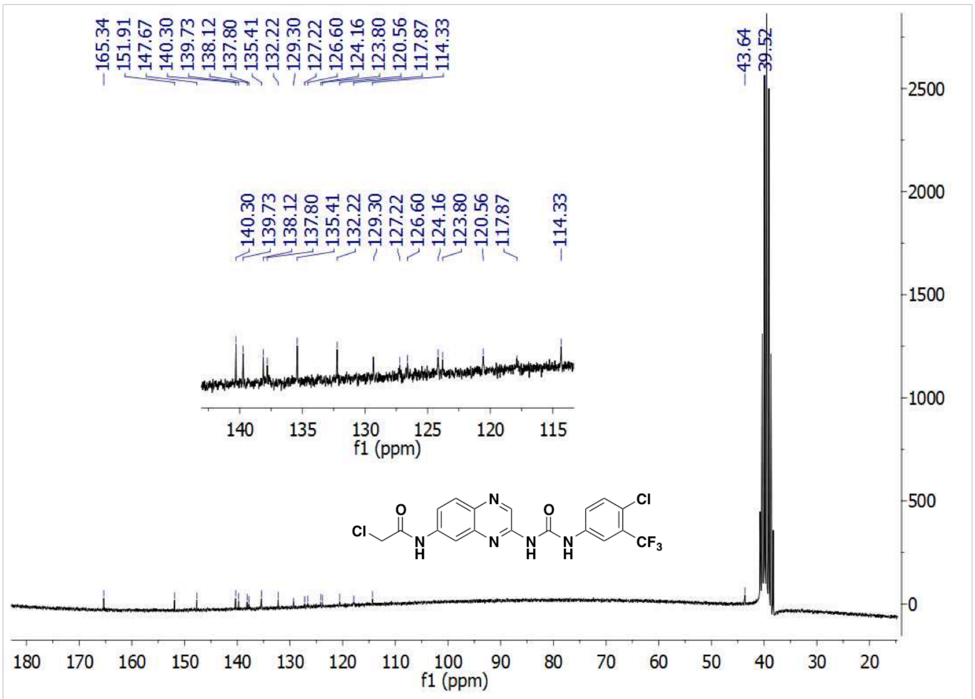


**Figure S47.** $^{13}$C NMR spectrum of compound 2-chloro-*N*-(3-(3-(4-chloro-3-(trifluoromethyl) phenyl)ureido) quinoxalin-6-yl)acetamide **(7c)** (DMSO-$d_6$/50MHz/TMS)

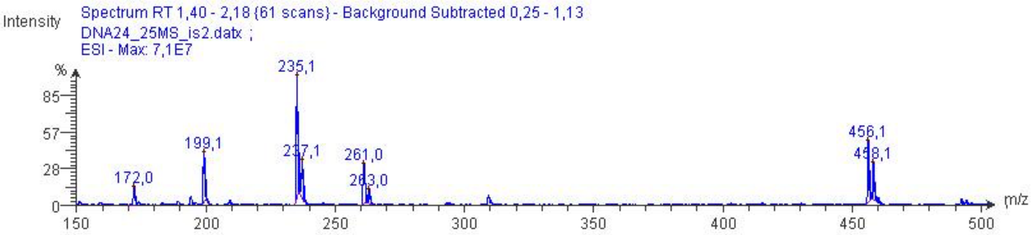


**Figure S48.** Mass spectrometry of compound 2-chloro-*N*-(3-(3-(4-chloro-3-(trifluoromethyl) phenyl)ureido) quinoxalin-6-yl)acetamide **(7c)** in negative mode (ESI-).

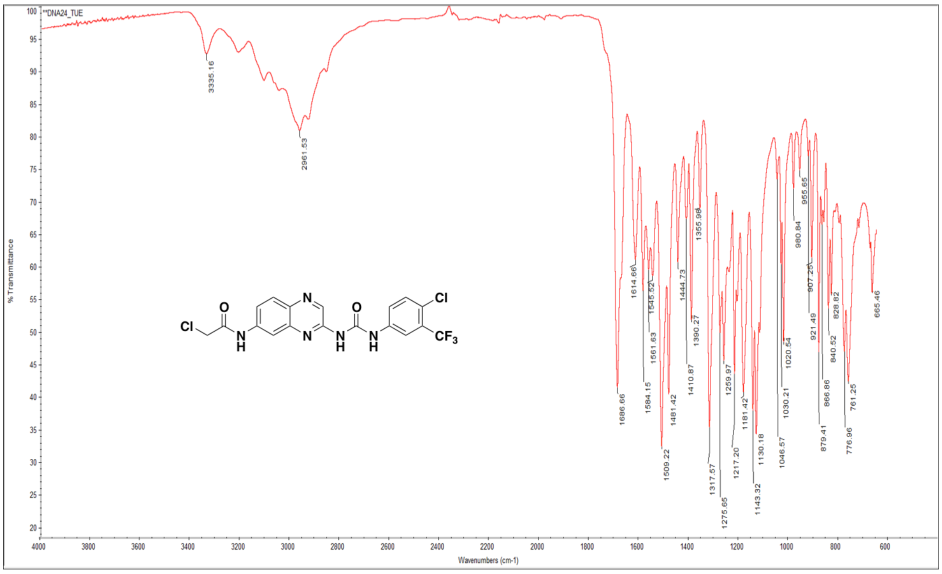


**Figure S49.** Infra red spectrum (ATR-FTIR) of compound 2-chloro-*N*-(3-(3-(4-chloro-3-(trifluoromethyl)phenyl)ureido) quinoxalin-6-yl)acetamide **(7c)** (bands in $cm^{-}$).

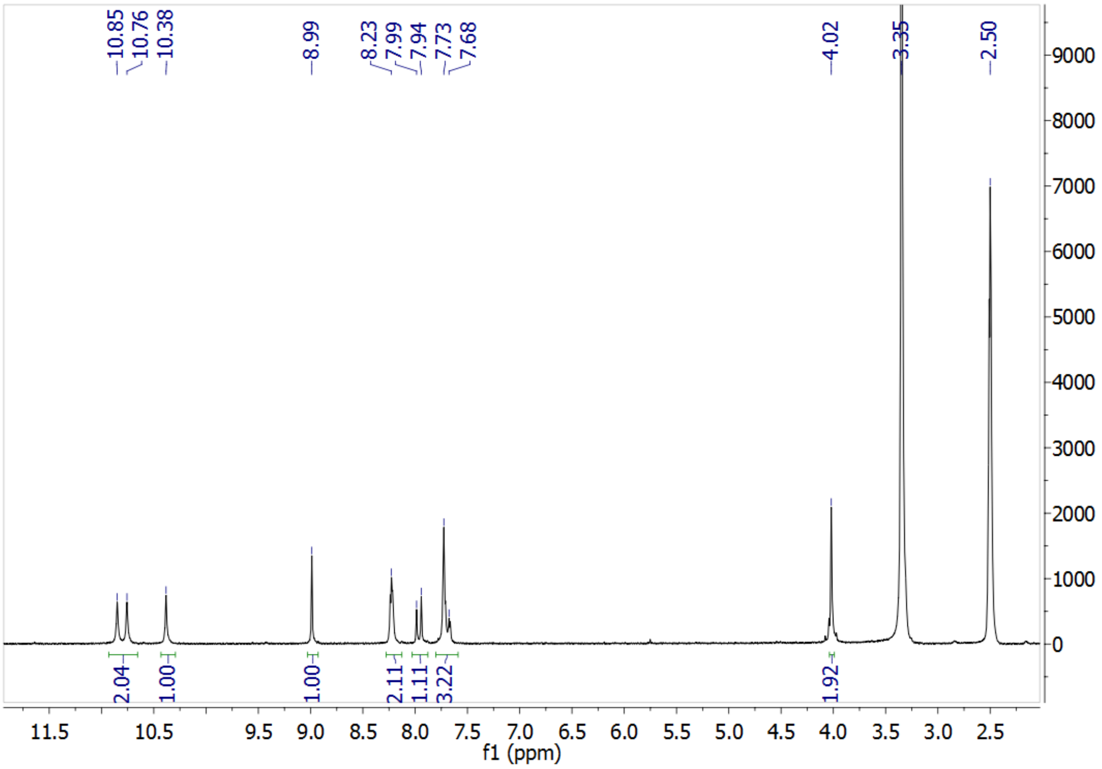

**Figure S50.** $^{1}H$ NMR spectrum of compound 2-chloro- *N*-(3-(3-(4-chloro-3-(trifluoromethyl) phenyl)ureido)quinoxalin-6-yl)-2-cianoacetamide **(7d)** (DMSO-$d_6$/200MHz/TMS)

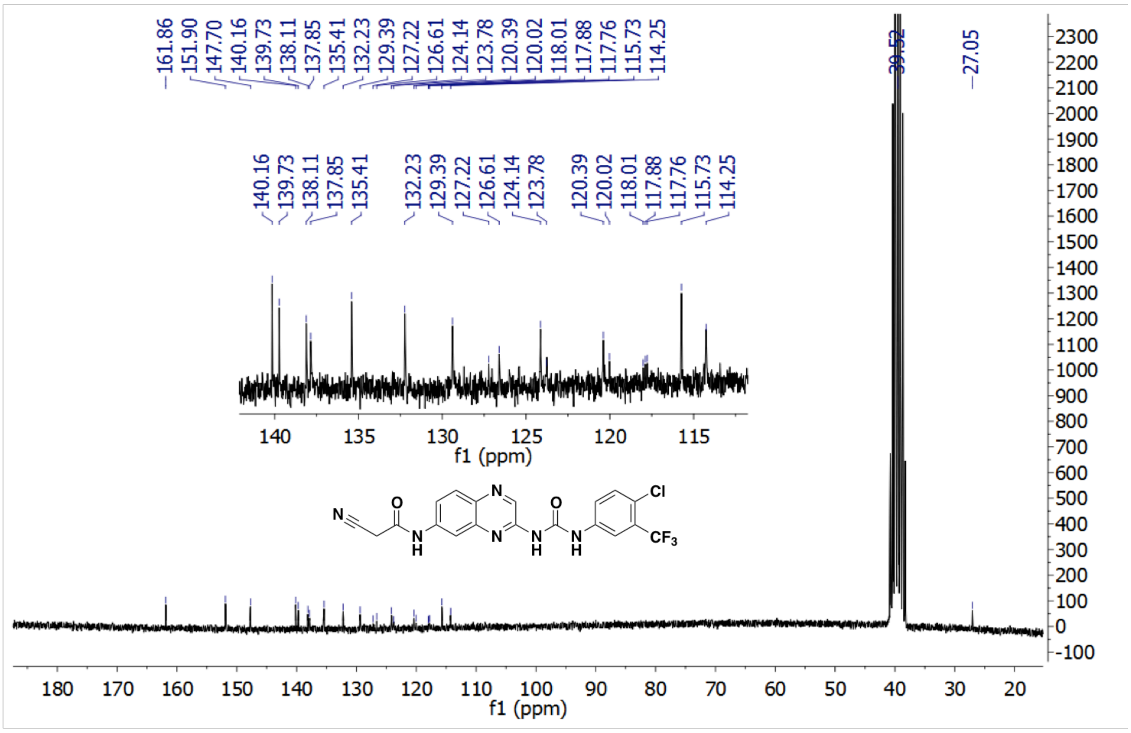

**Figure S51.** $^{13}C$ NMR spectrum of compound 2-chloro- *N*-(3-(3-(4-chloro-3-(trifluoromethyl) phenyl)ureido)quinoxalin-6-yl)-2-cianoacetamide **(7d)** (DMSO-$d_6$/50MHz/TMS)

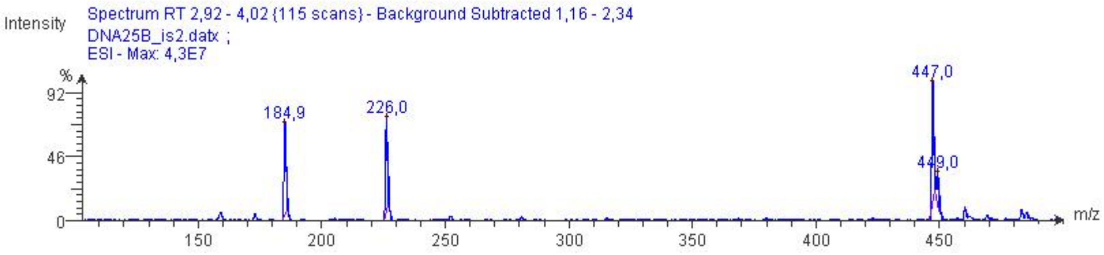


**Figure S52.** Mass spectrometry of compound *N*-(3-(3-(4-chloro-3-(trifluoromethyl)phenyl)ureido) quinoxalin-6-yl) -2-cianoacetamide **(7d)** in negative mode (ESI-).

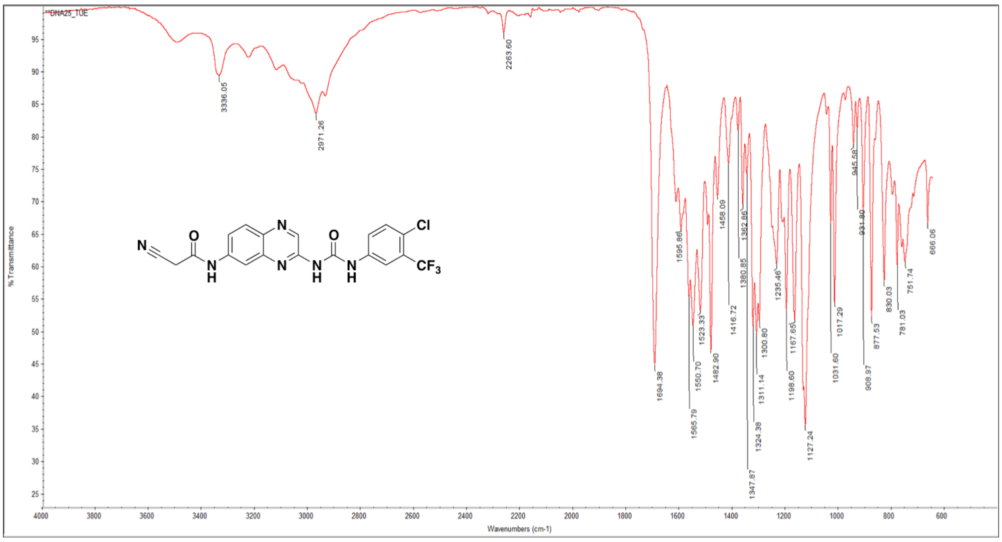


**Figure S53.** Infra red spectrum (ATR-FTIR) of compound *N*-(3-(3-(4-chloro-3-(trifluoromethyl) phenyl)ureido) quinoxalin-6-yl)-2-cianoacetamide **(7d)** (bands in cm$^{-}$).

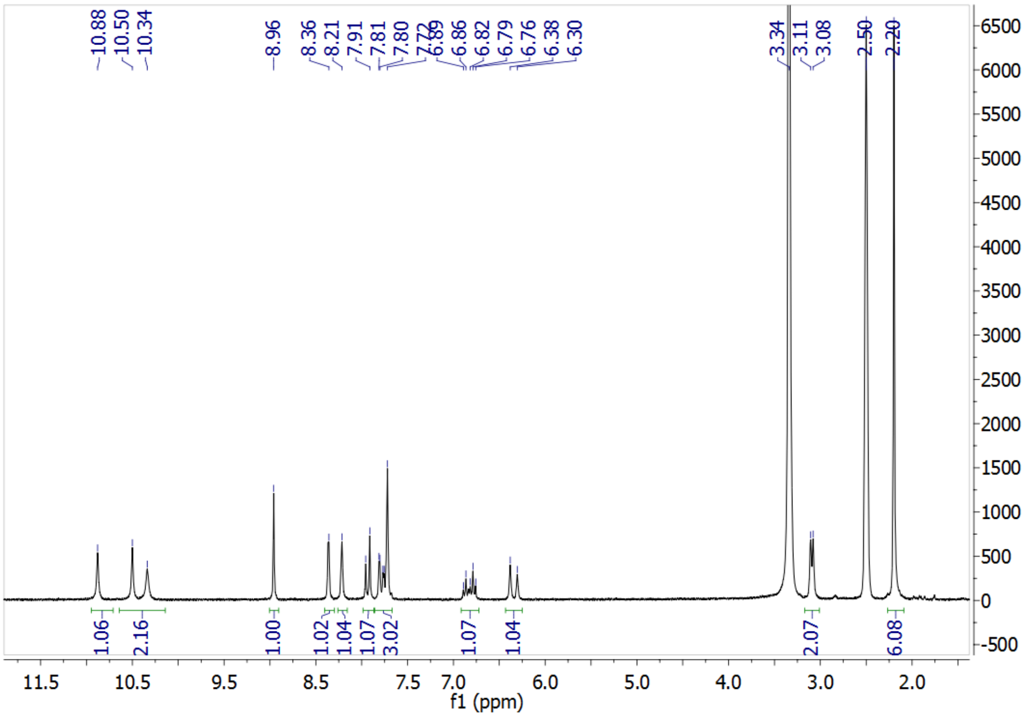


**Figure S54.** $^{1}$H NMR spectrum of compound (*E*)-*N*-(3-(3-(4-chloro-3-(trifluoromethyl)phenyl) ureido)quinoxalin-6-yl)-4-(dimethylamino)but-2-enamide **(7e)** (DMSO-$d_6$/200MHz/TMS)

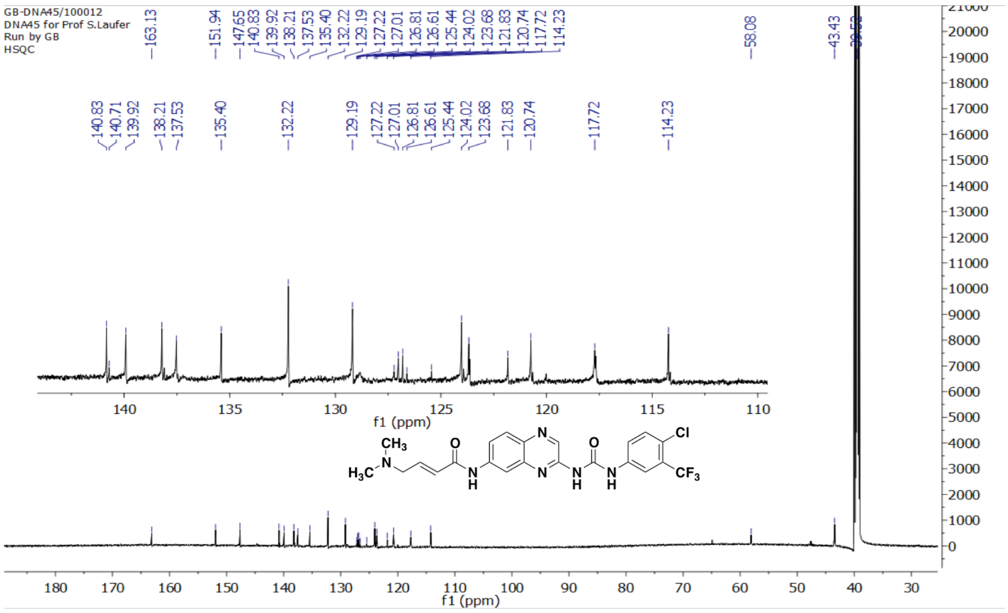


**Figure S55.** $^{13}$C NMR spectrum of compound (*E*)-*N*-(3-(3-(4-chloro-3-(trifluoromethyl)phenyl) ureido)quinoxalin-6-yl)-4-(dimethylamino)but-2-enamide **(7e)** (DMSO-$d_6$/150MHz/TMS)

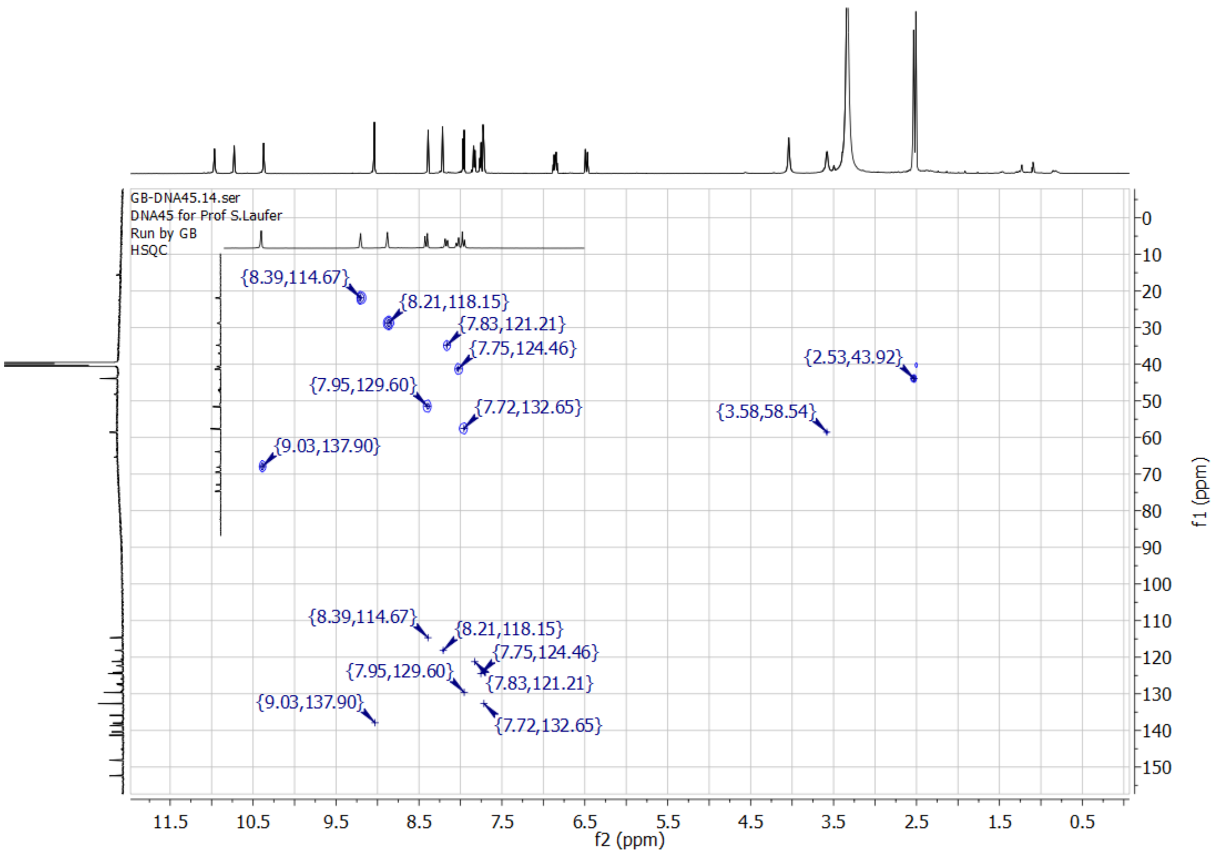


**Figure S56.** HSQC spectrum of compound (*E*)-*N*-(3-(3-(4-chloro-3-(trifluoromethyl)phenyl)ureido)quinoxalin-6-yl)-4-(dimethylamino)but-2-enamide **(7e)** (DMSO-$d_6$/150MHz/TMS)

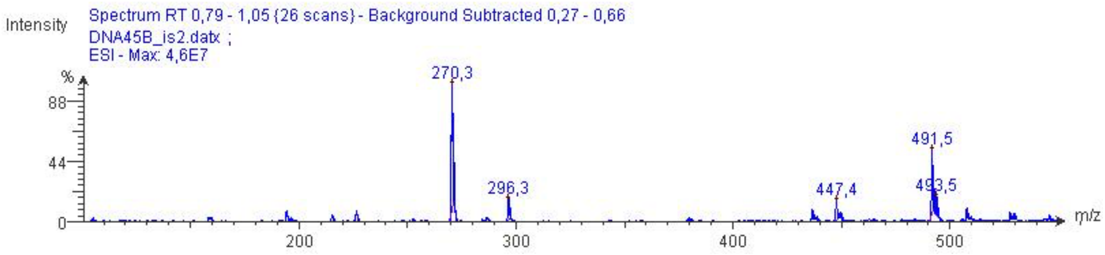


**Figure S57.** Mass spectrometry of compound *E*)-*N*-(3-(3-(4-chloro-3-(trifluoromethyl) phenyl)ureido) quinoxalin-6-yl)-4-(dimethylamino)but-2-enamide **(7e)** in negative mode (ESI-).

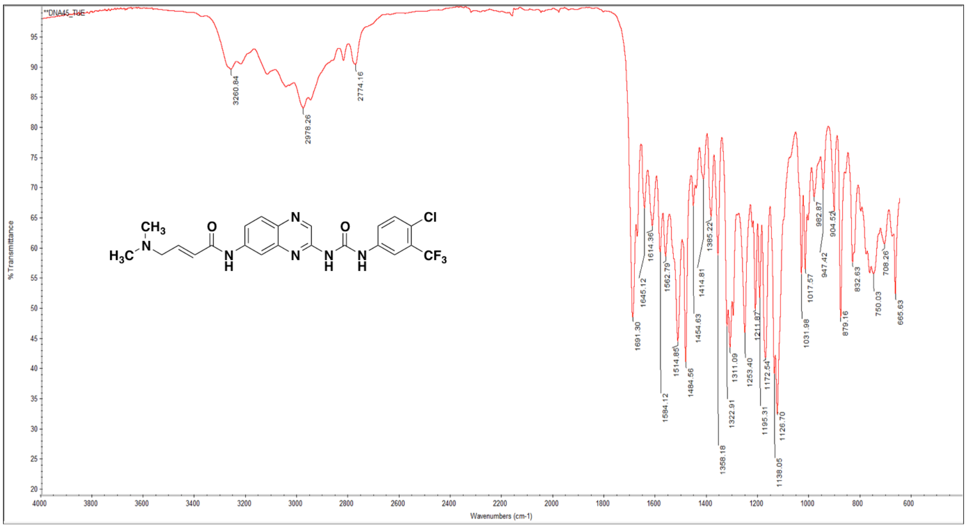

**Figure S58.** Infra red spectrum (ATR-FTIR) of compound (*E*)-*N*-(3-(3-(4-chloro-3-(trifluoromethyl) phenyl)ureido) quinoxalin-6-yl)-4-(dimethylamino)but-2-enamide **(7e)** (bands in cm-).

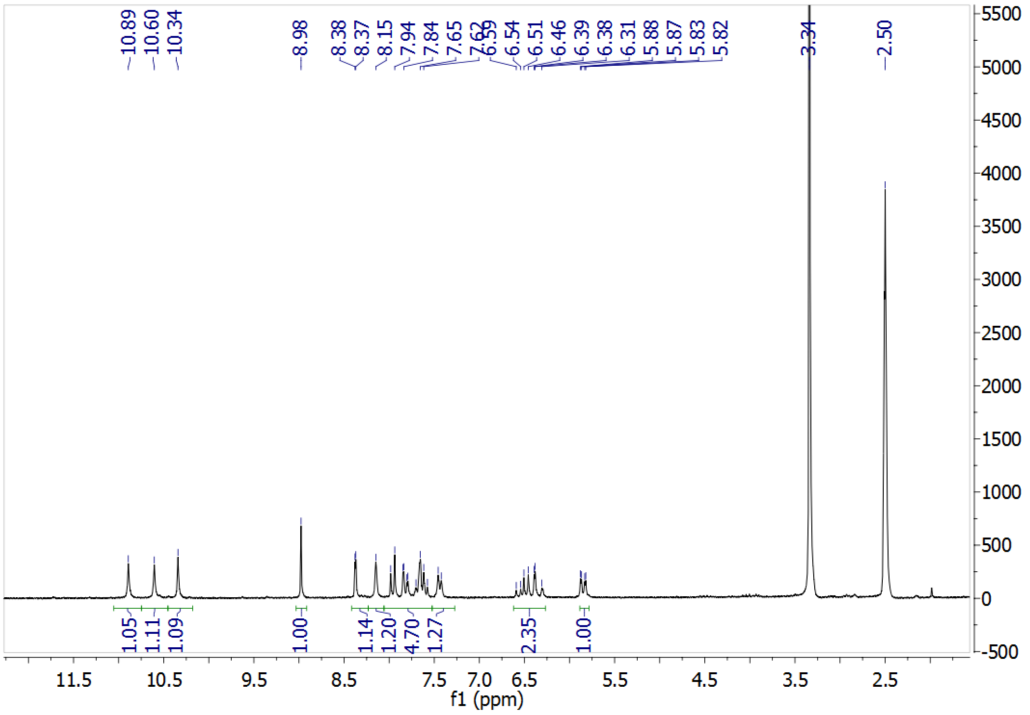


**Figure S59.** ¹H NMR spectrum of compound *N*-(3-(3-(3-(trifluoromethyl)phenyl)ureido) quinoxalin-6-yl)acrylamide **(7f)** (DMSO-$d_6$/200MHz/TMS)

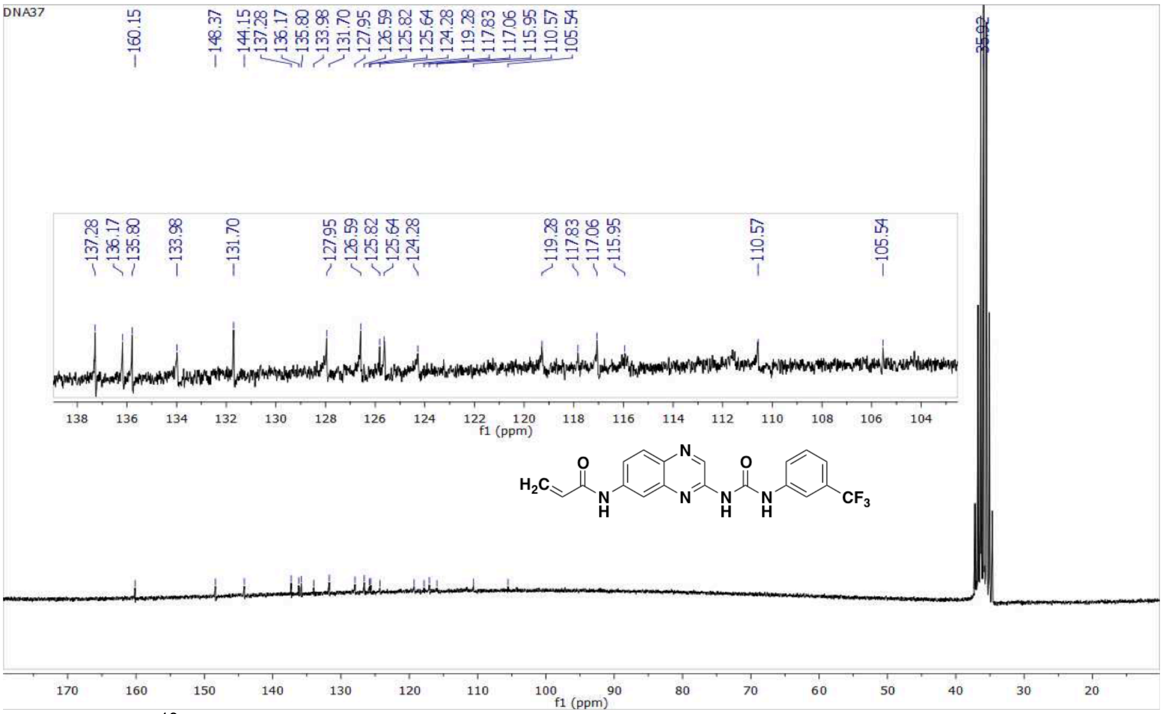


**Figure S60.** $^{13}C$ NMR spectrum of compound *N*-(3-(3-(3-(trifluoromethyl)phenyl)ureido) quinoxalin-6-yl)acrylamide **(7f)** (DMSO-$d_6$/50MHz/TMS)

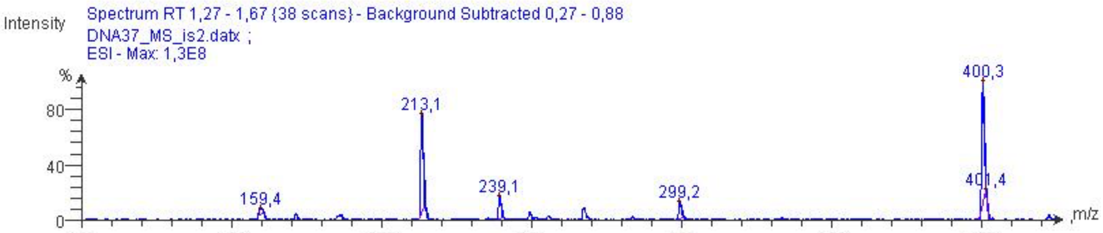


**Figure S61.** Mass spectrometry of compound *N*-(3-(3-(3-(trifluoromethyl)phenyl)ureido) quinoxalin-6-yl) acrylamide **(7f)** in negative mode (ESI-)

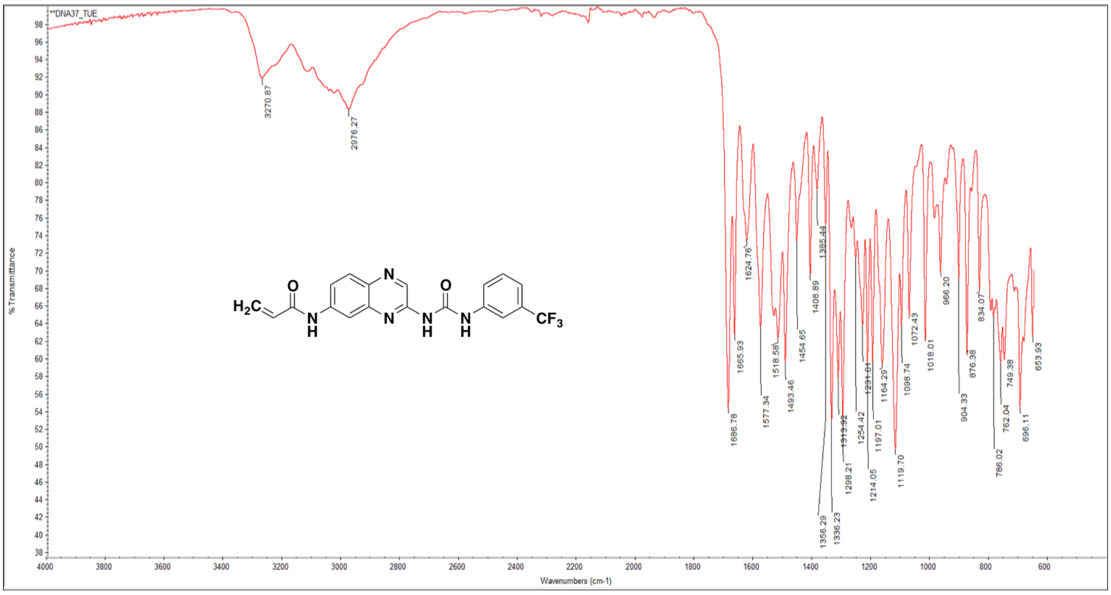


**Figure S62.** Infra red spectrum (ATR-FTIR) do *N*-(3-(3-(3-(trifluoromethyl)phenyl)ureido) quinoxalin-6-yl)acrylamide **(7f)** (bands in $cm^{-}$).

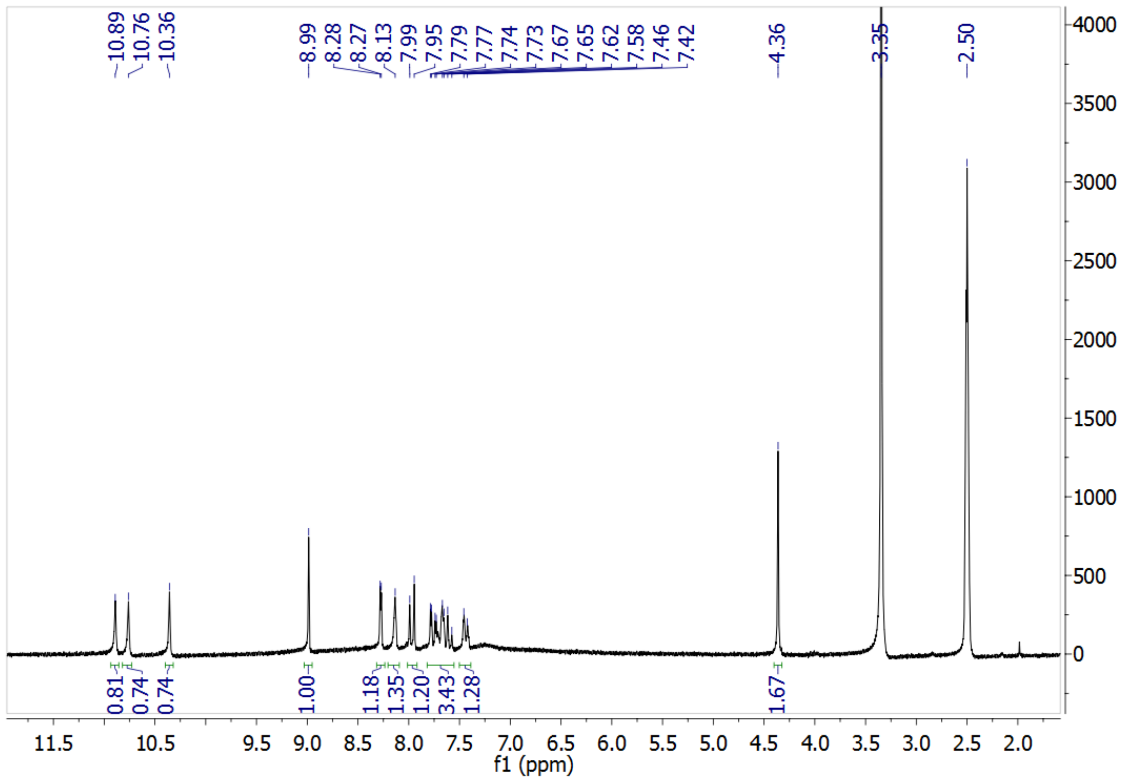

**Figure S63.** $^{1}$H NMR spectrum of compound 2-chloro-*N*-(3-(3-(3-(trifluoromethyl) phenyl)ureido) quinoxalin-6-yl) acetamide**(7g)** (DMSO-$d_6$/200MHz/TMS)

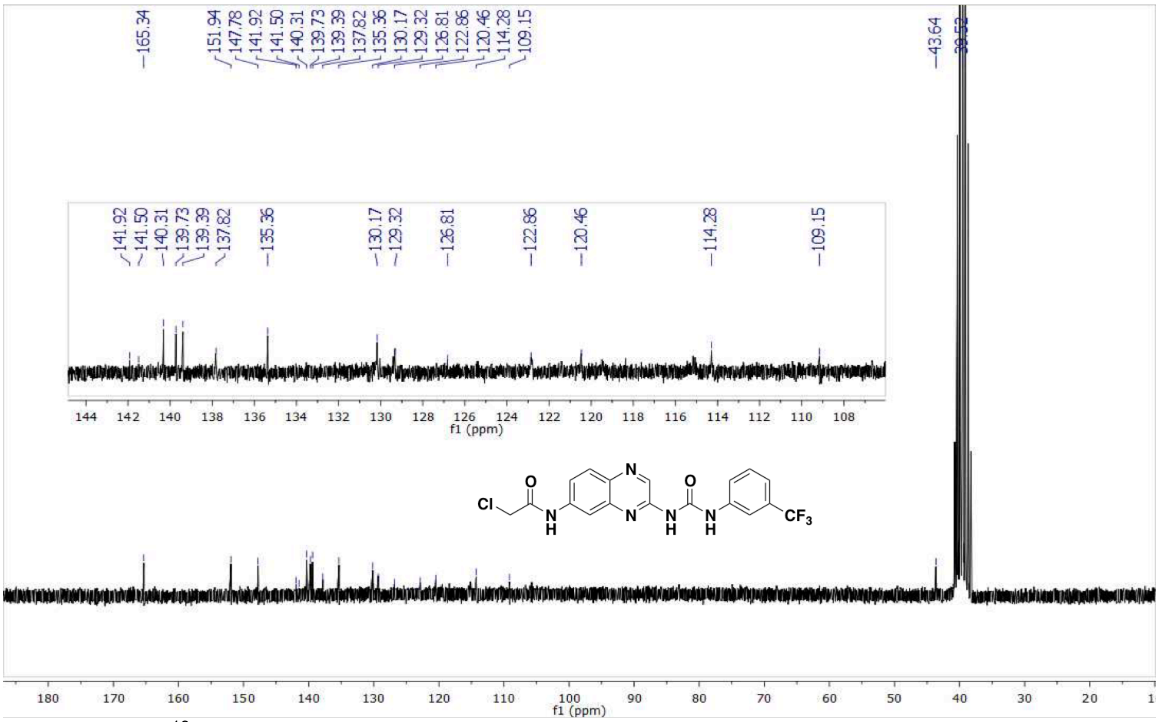

**Figure S64.** $^{13}$C NMR spectrum of compound 2-chloro-*N*-(3-(3-(3-(trifluoromethyl)phenyl) ureido) quinoxalin-6-yl) acetamide **(7g)** (DMSO-$d_6$/50MHz/TMS)

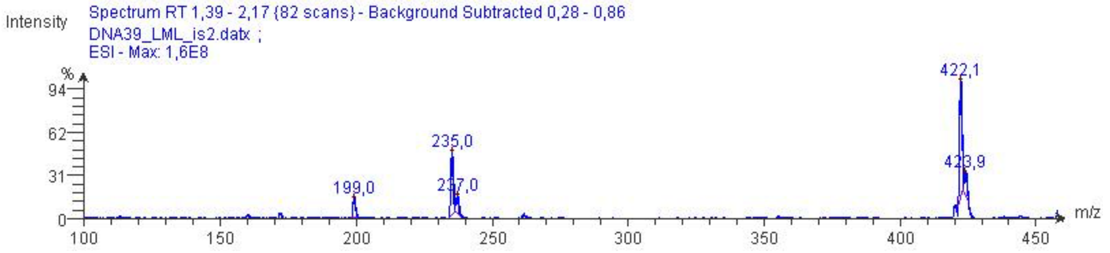


**Figure S65.** Mass spectrometry of compound 2-chloro-*N*-(3-(3-(3-(trifluoromethyl)phenyl) ureido) quinoxalin-6-yl) acetamide **(7g)** in negative mode (ESI-).

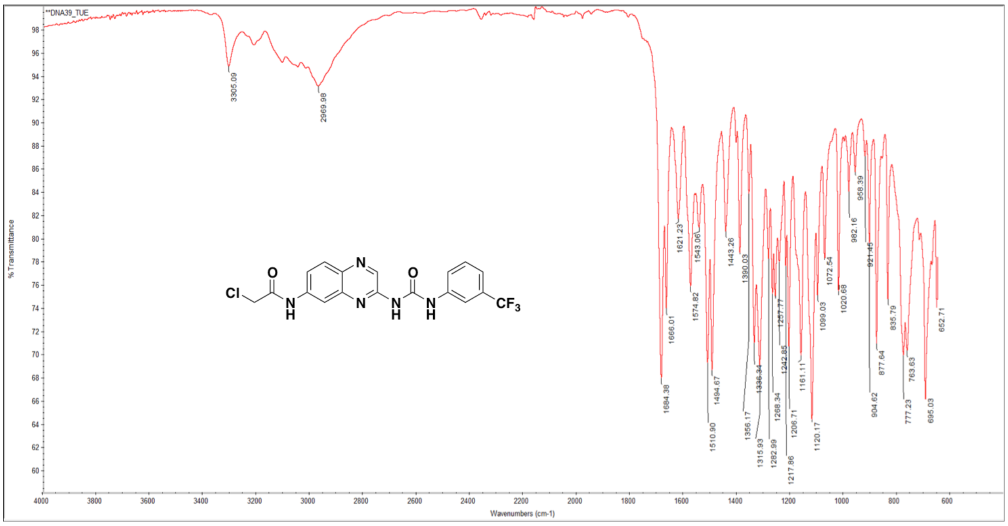


**Figure S66.** Infra red spectrum (ATR-FTIR) do 2-chloro-*N*-(3-(3-(3-(trifluoromethyl)phenyl) ureido) quinoxalin-6-yl) acetamide **(7g)** (bands in cm$^{-}$).

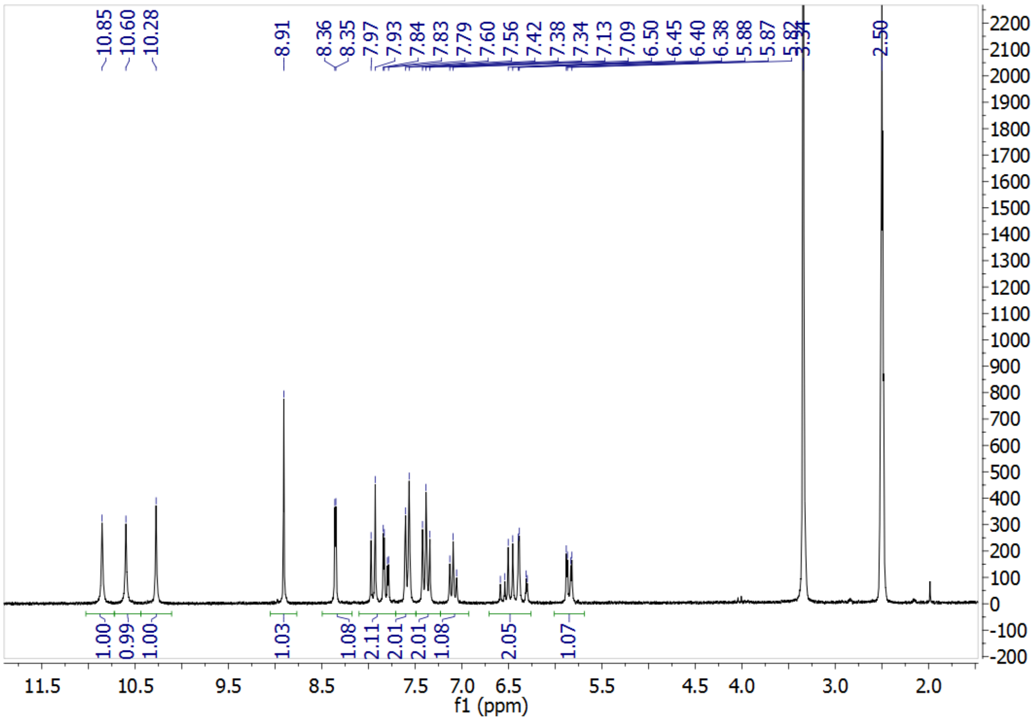


**Figure S67.** $^{1}$H NMR spectrum of compound *N*-(3-(3-phenylureido)quinoxalin-6-yl)acrylamide **(7h)** (DMSO-$d_6$/200MHz/TMS)

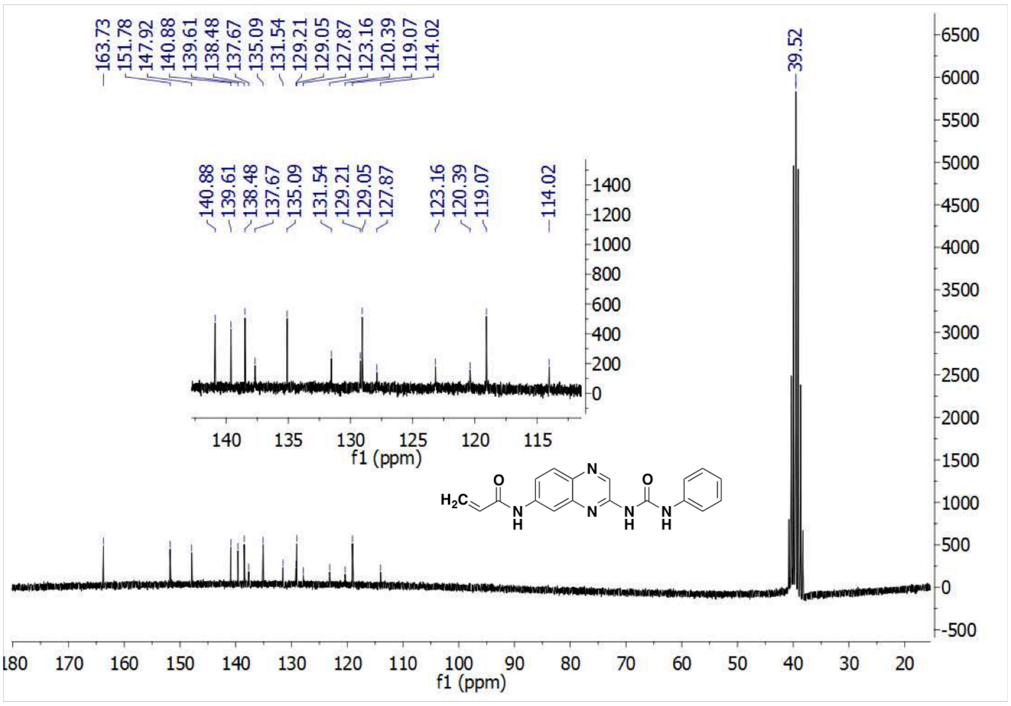


**Figure S68.** $^{13}$C NMR spectrum of compound *N*-(3-(3-phenylureido)quinoxalin-6-yl) acrylamide **(7h)** (DMSO-$d_6$/50MHz/TMS)

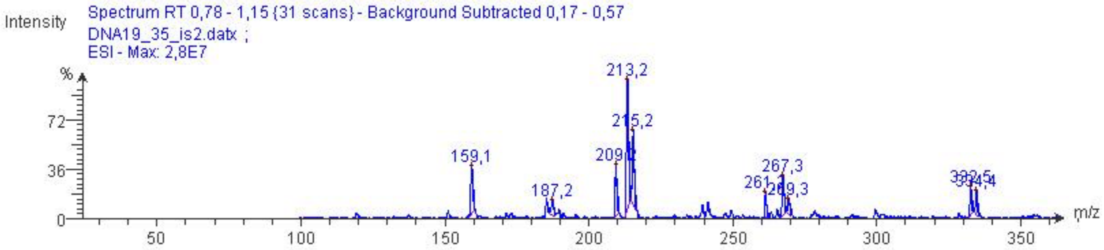


**Figure S69.** Mass spectrometry of compound *N*-(3-(3-phenylureido)quinoxalin-6-yl)acrylamide **(7h)** in negative mode (ESI-).

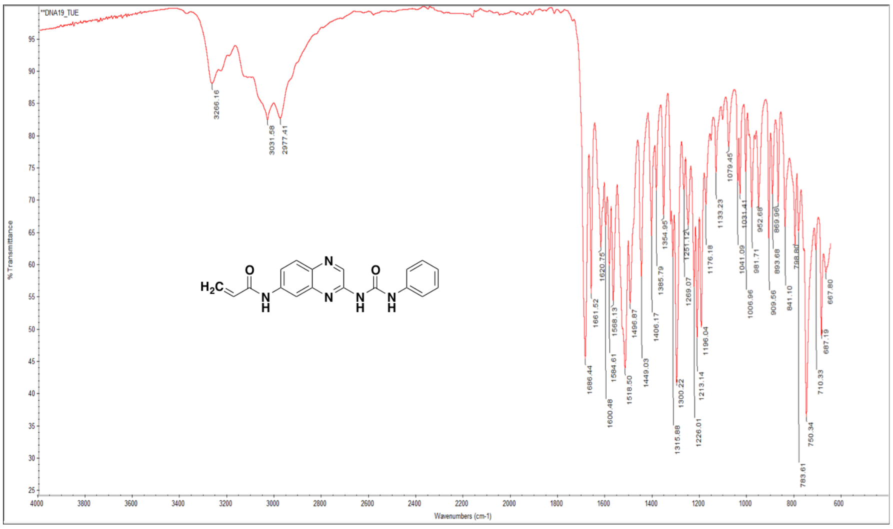

**Figure S70.** Infra red spectrum (ATR-FTIR) of compound *N*-(3-(3-phenylureido)quinoxalin-6-yl)acrylamide **(7h)** (bands in cm$^{-}$).

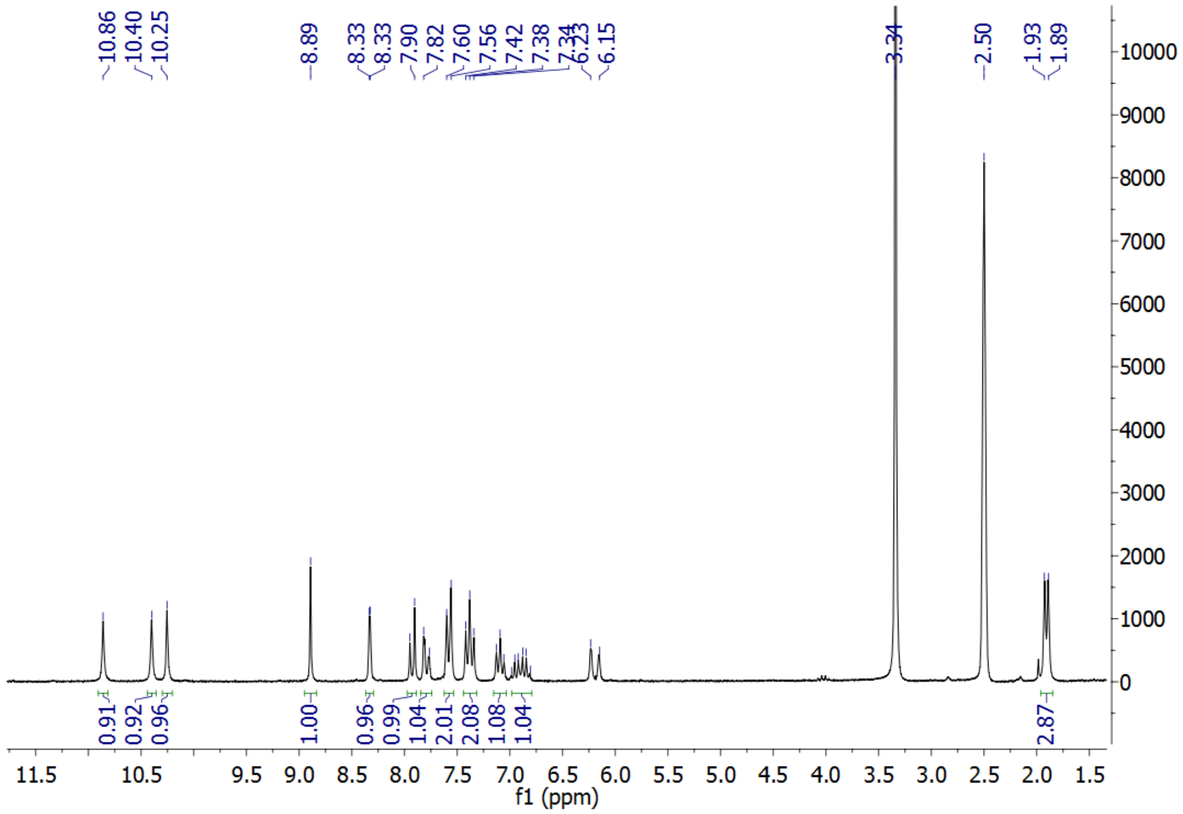


**Figure S71.** $^{1}$H NMR spectrum of compound 1(*E*)-*N*-(3-(3-phenylureido)quinoxalin-6-yl)but-2-enamide **(7i)** (DMSO-d6/200MHz/TMS)

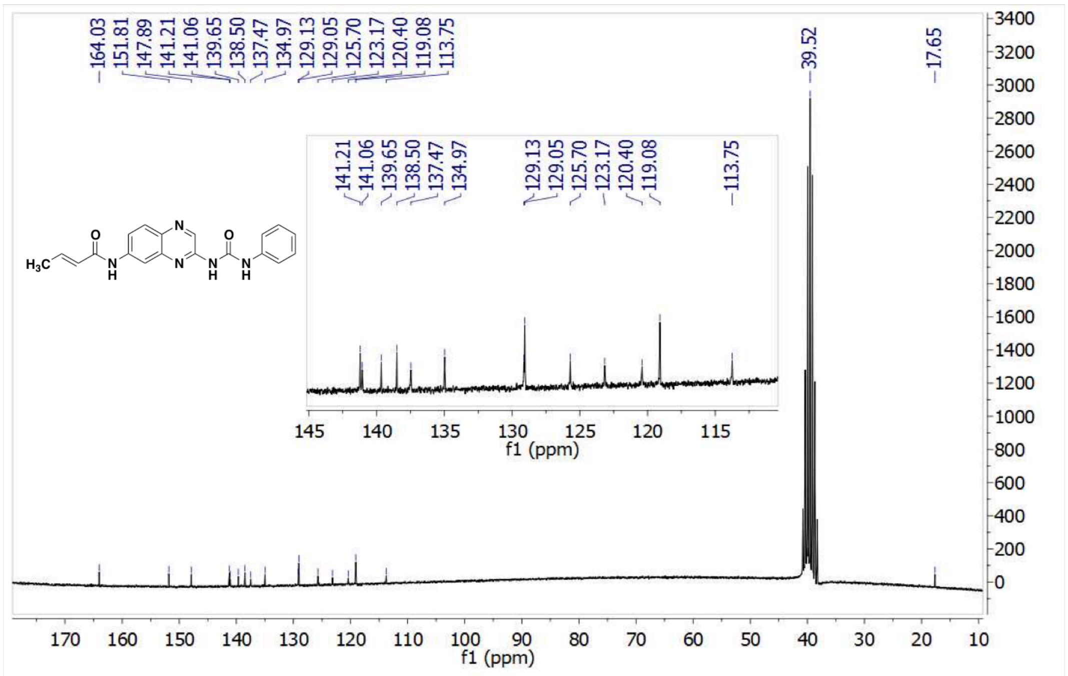


**Figure S72.** $^{13}$C NMR spectrum of compound (*E*)-*N*-(3-(3-phenylureido)quinoxalin-6-yl)but-2-enamide **(7i)** (DMSO-d6/50MHz/TMS)

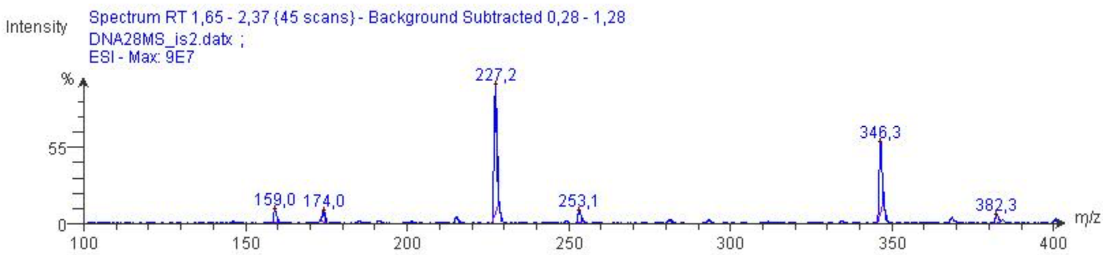


**Figure S73.** Mass spectrometry of compound (*E*)-*N*-(3-(3-phenylureido)quinoxalin-6-yl)but-2-enamide **(7i)** in negative mode (ESI-).

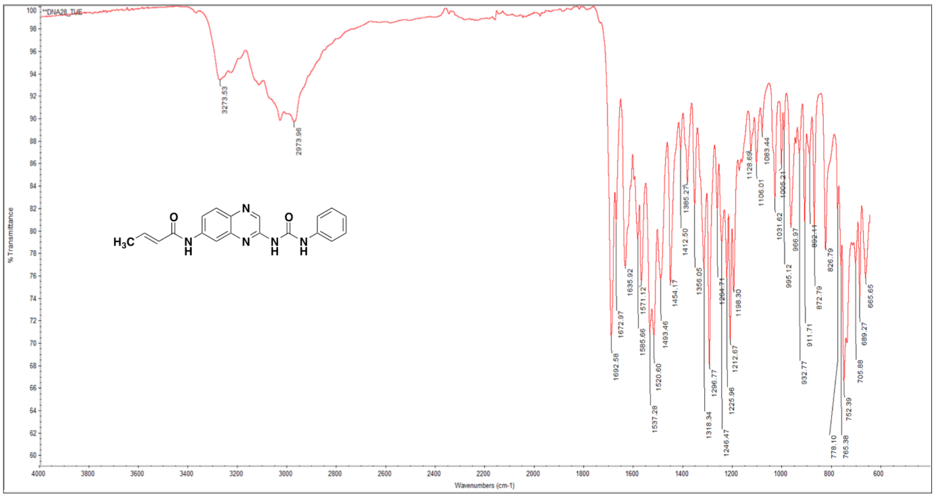

**Figure S74.** Infra red spectrum (ATR-FTIR) of compound (*E*)-*N*-(3-(3-phenylureido)quinoxalin-6-yl)but-2-enamide **(7i)** (bands in cm$^{-}$).

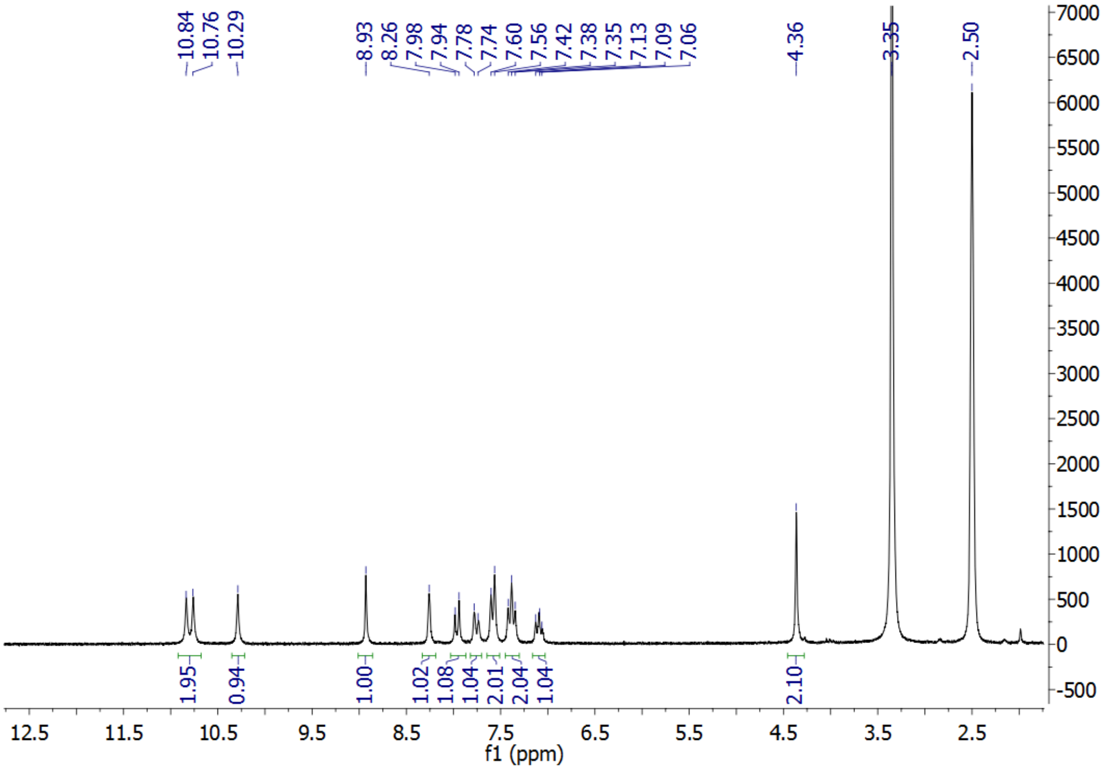


**Figure S75.** $^{1}$H NMR spectrum of compound 2-chloro-*N*-(3-(3-phenylureido)quinoxalin-6-yl)acetamide **(7j)** DMSO-d$_6$/200MHz/TMS)

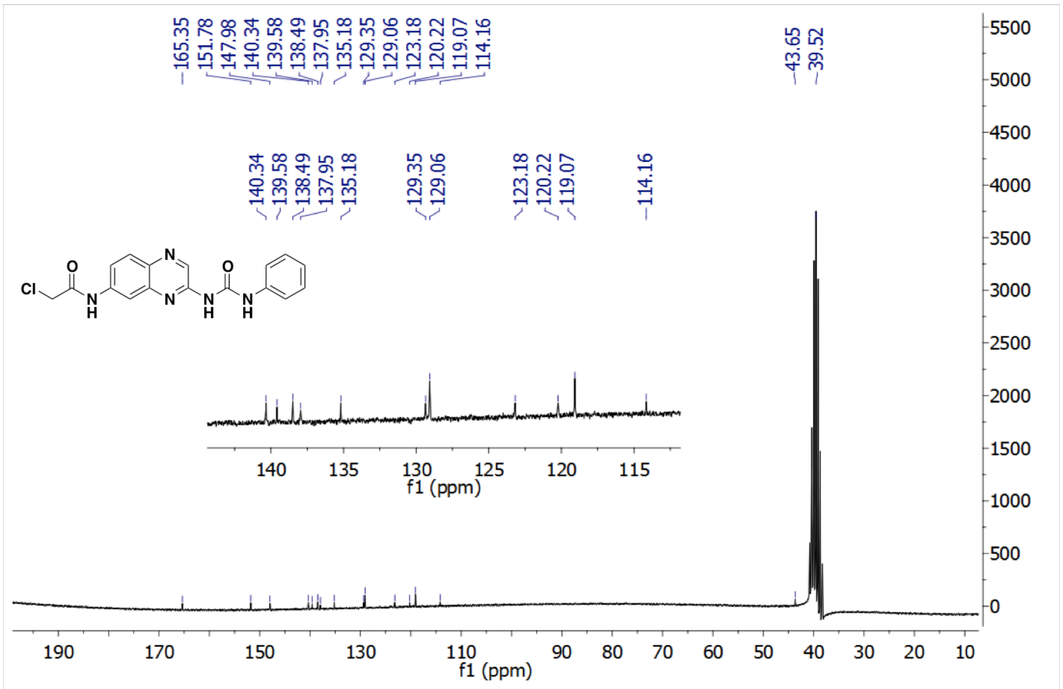


**Figure S76.** $^{13}C$ NMR spectrum do 2-chloro-*N*-(3-(3-phenylureido)quinoxalin-6-yl)acetamide **(7j)** (DMSO-$d_6$/50MHz/TMS)

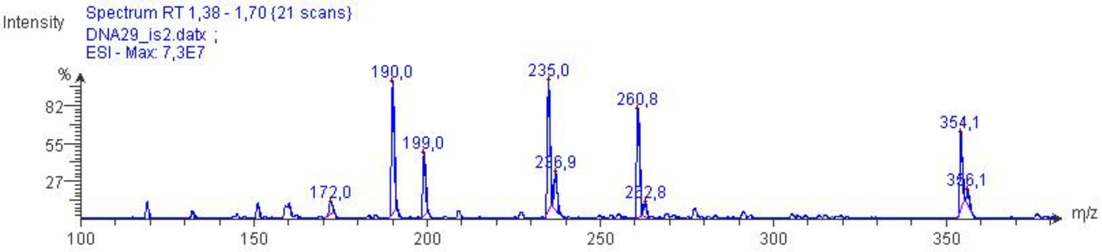


**Figure S77.** Mass spectrometry of compound 2-chloro-*N*-(3-(3-phenylureido)quinoxalin-6-yl) acetamide **(7j)** in negative mode (ESI-).

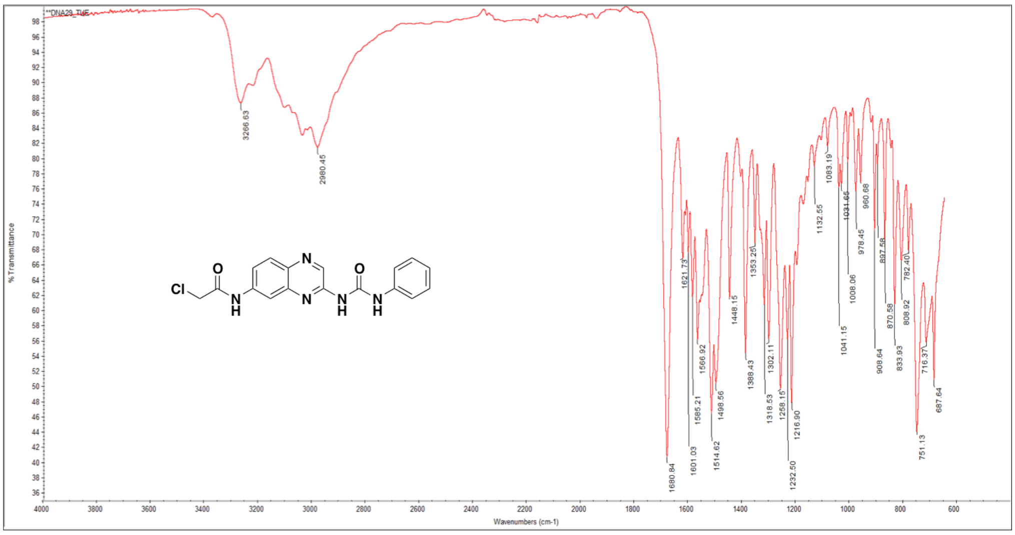


**Figure S78.** Infra red spectrum (ATR-FTIR) of compound 2-chloro-*N*-(3-(3-phenylureido) quinoxalin-6-yl)acetamide **(7j)** (bands in $cm^{-}$).

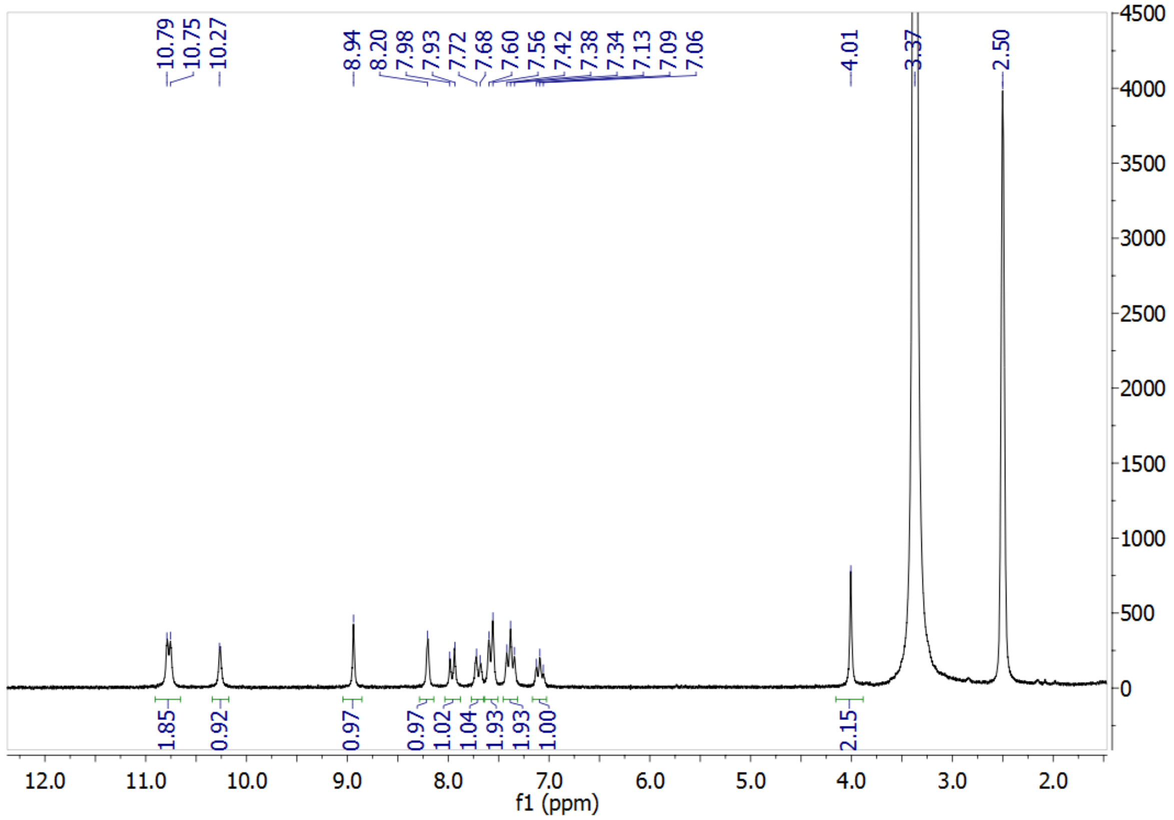


**Figure S79.** $^{1}$H NMR spectrum of compound 2-ciano-*N*-(3-(3-phenylureido)quinoxalin-6-yl)acetamide **(7k)** (DMSO-d$_6$/200MHz/TMS)

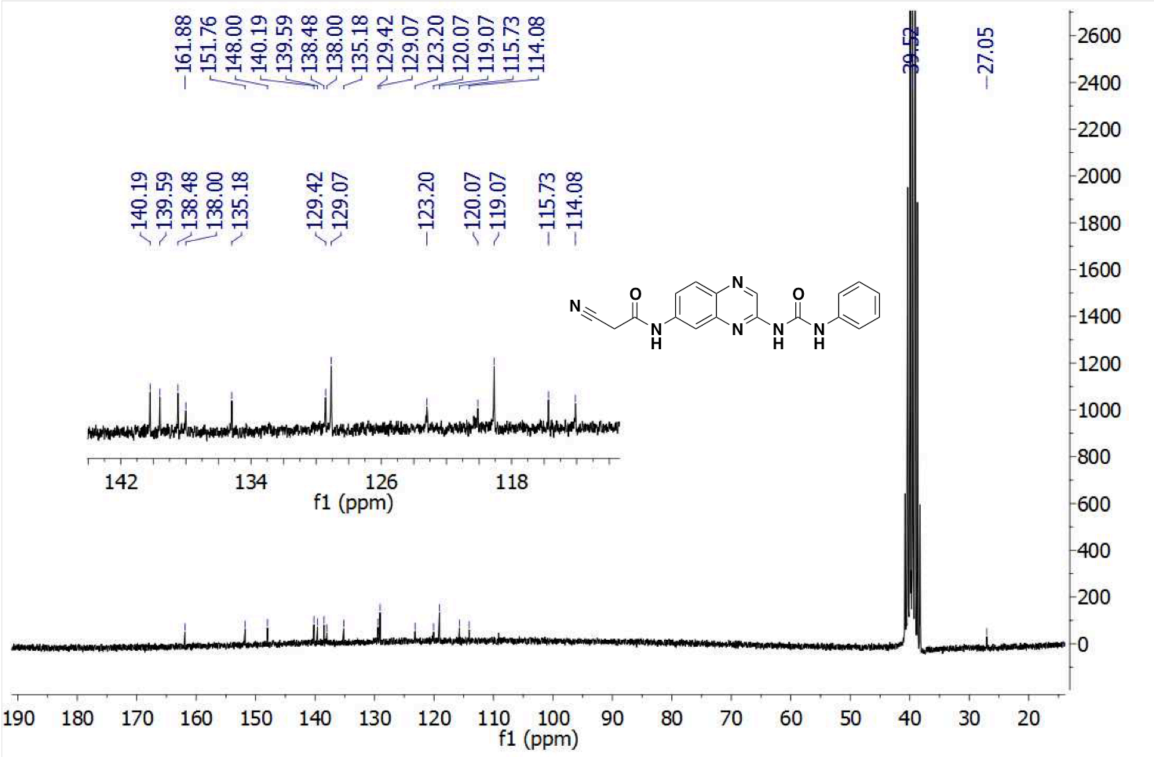


**Figure S80.** $^{13}$C NMR spectrum of compound 2-ciano-*N*-(3-(3-phenylureido)quinoxalin-6-yl)acetamide **(7k)** (DMSO-d$_6$/50MHz/TMS)

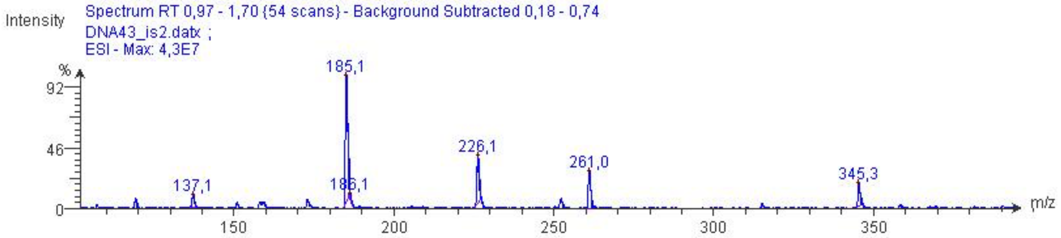


**Figure S81.** Mass spectrometry of compound 2-ciano-*N*-(3-(3-phenylureido)quinoxalin-6-yl) acetamide **(7k)** in negative mode (ESI-).

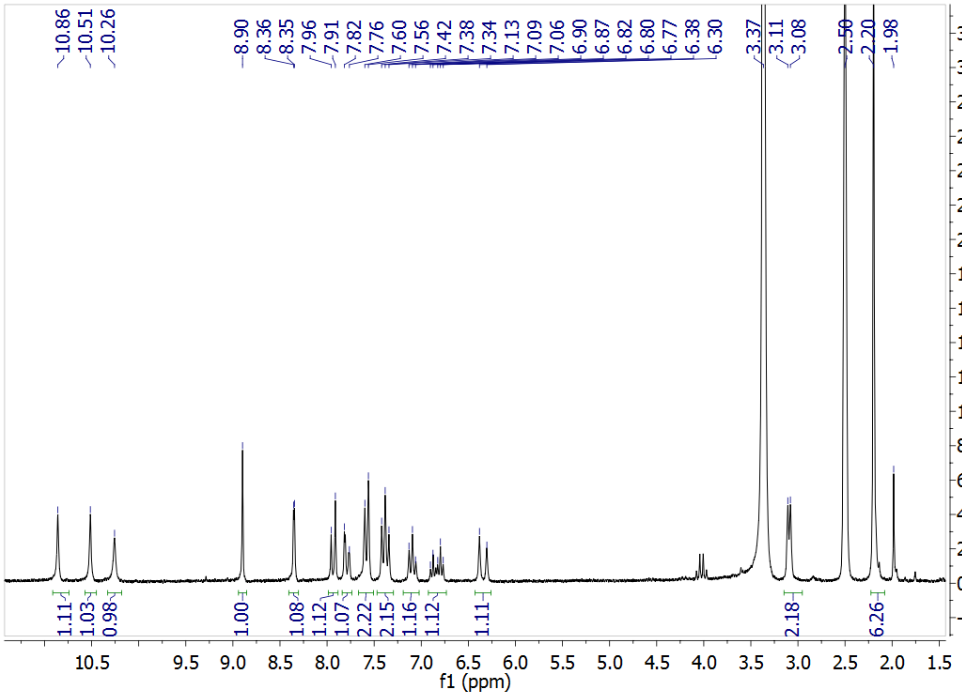


**Figure S82.** $^{1}$H NMR spectrum of compound (*E*)-4-(dimethylamino)-*N*-(3-(3-phenylureido) quinoxalin-6-yl)but-2-enamide **(7l)** (DMSO-d$_6$/200MHz/TMS)

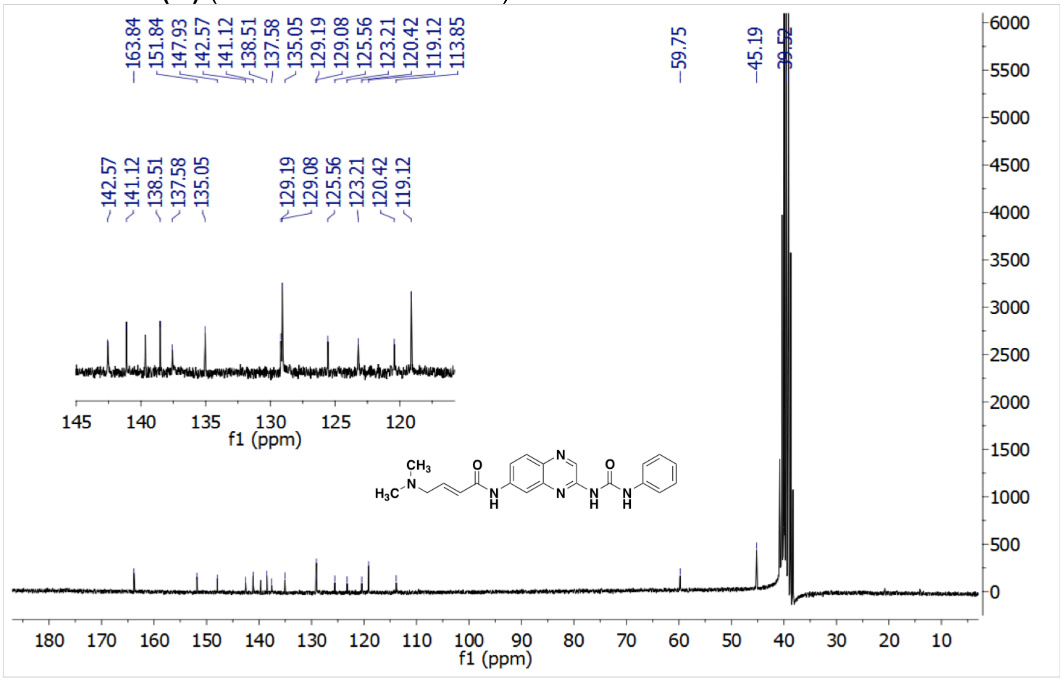


**Figure S83.** $^{13}$C NMR spectrum of compound *E*)-4-(dimethylamino)-*N*-(3-(3-phenylureido) quinoxalin-6-yl)but-2-enamide **(7l)** (DMSO-d$_6$/50MHz/TMS)

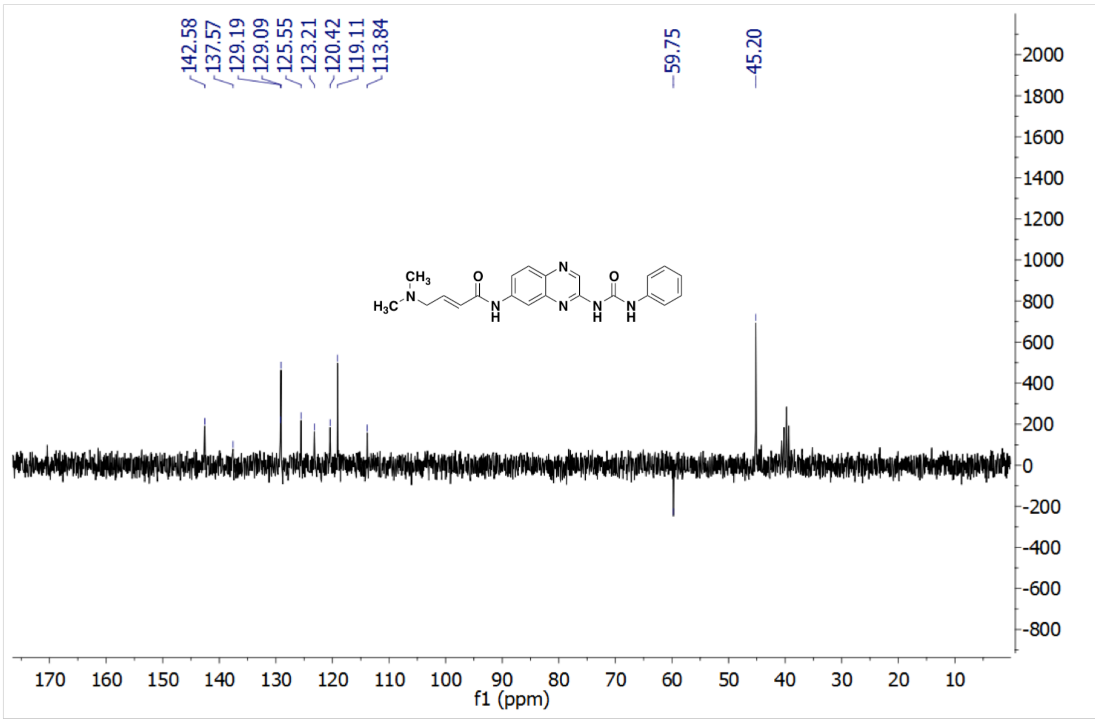


**Figure S84.** Espectro de DEPT 135 of compound *E*)-4-(dimethylamino)-*N*-(3-(3-phenylureido)quinoxalin-6-yl)but-2-enamide **(7l)** (DMSO-d6/50MHz/TMS).

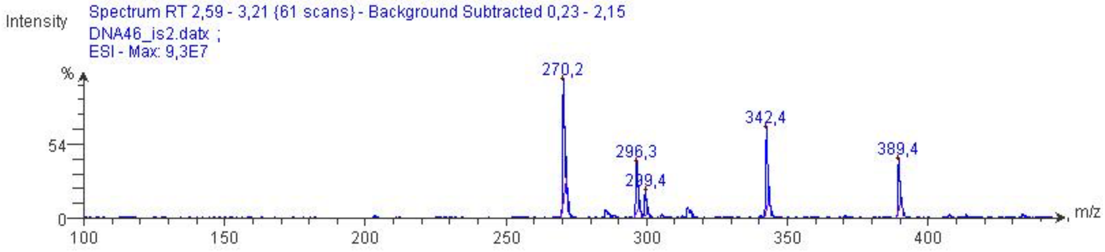


**Figure S85.** Mass spectrometry of compound (*E*)-4-(dimethylamino)-*N*-(3-(3-phenylureido) quinoxalin-6-yl)but-2-enamide **(7l)** in negative mode (ESI-).

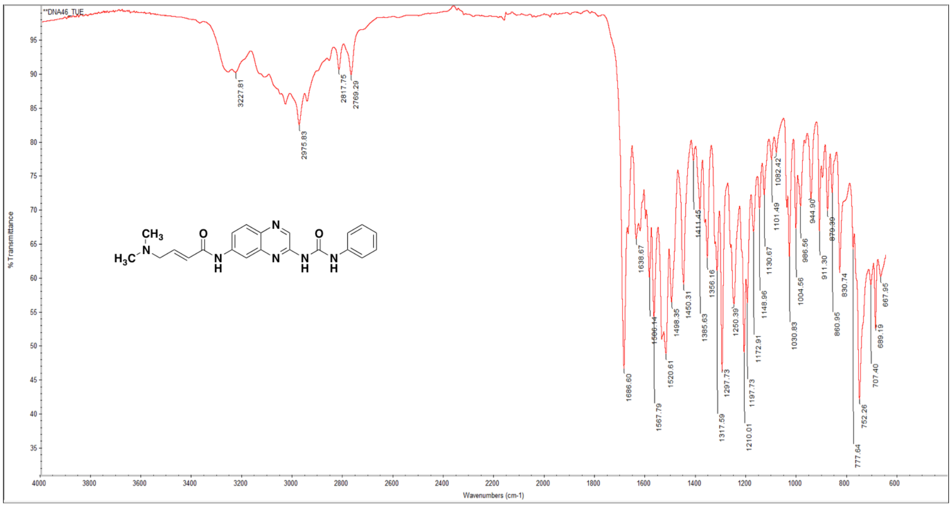

**Figure S86.** Infra red spectrum (ATR-FTIR) of compound (*E*)-4-(dimethylamino)-*N*-(3-(3-phenylureido) quinoxalin-6-yl)but-2-enamide **(7l)** (bands in cm$^{-}$).

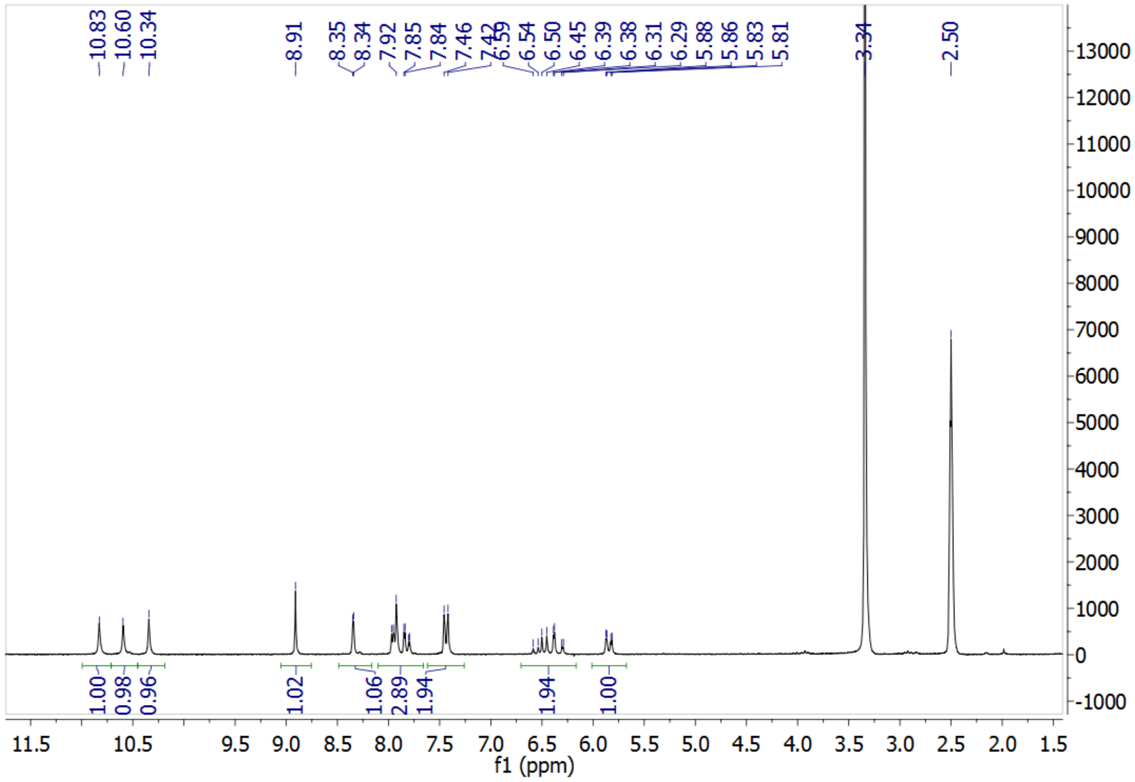

**Figure S87.** $^{1}$H NMR spectrum of compound *N*-(3-(3-(3-chloro-4-fluorophenyl) ureido)quinoxalin-6-yl) acrylamide **(7m)** (DMSO-$d_6$/200MHz/TMS)

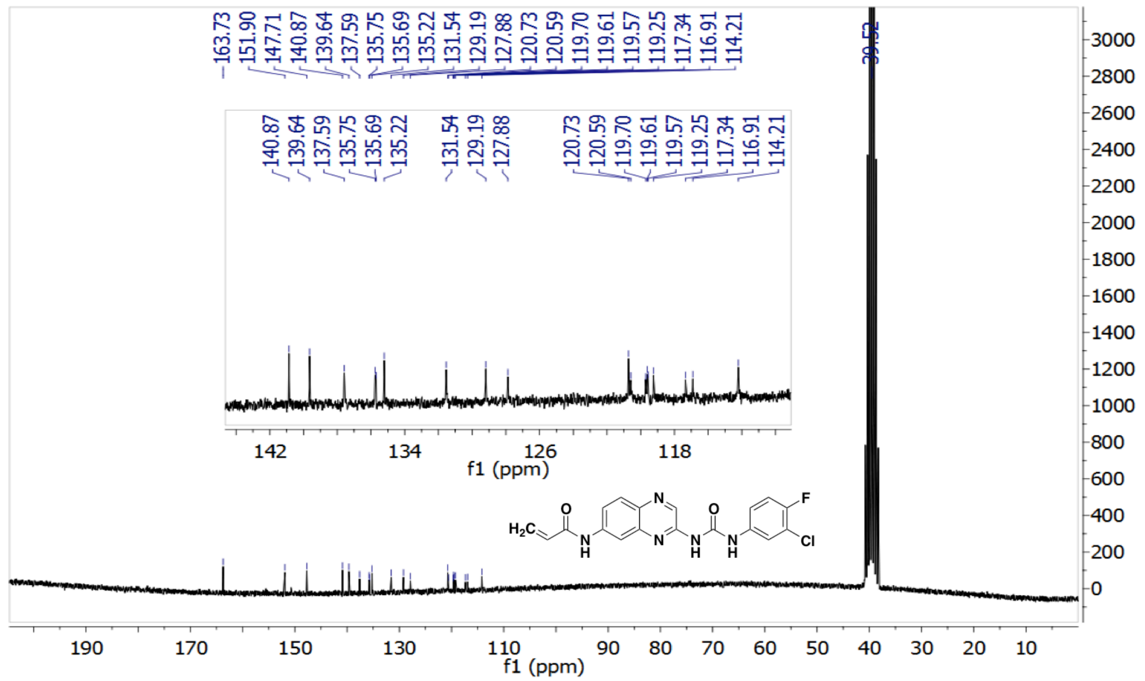

**Figure S88.** $^{13}$C NMR spectrum of compound *N*-(3-(3-(3-chloro-4-fluorophenyl)ureido) quinoxalin-6-yl) acrylamide **(7m)** (DMSO-$d_6$/50MHz/TMS)

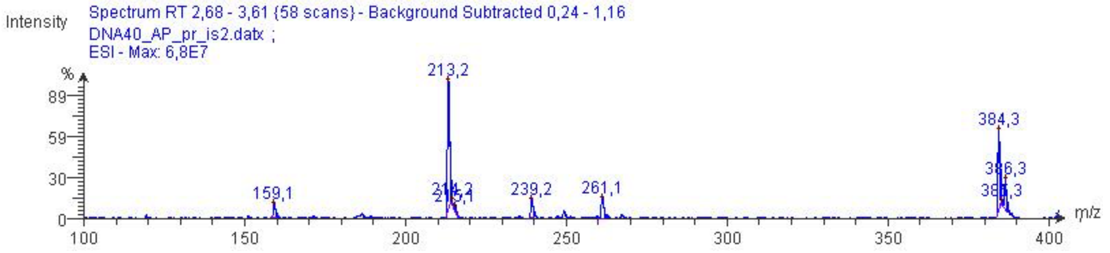


**Figure S89.** Mass spectrometry of compound *N*-(3-(3-(3-chloro-4-fluorophenyl)ureido) quinoxalin-6-yl) acrylamide **(7m)** in negative mode (ESI-).

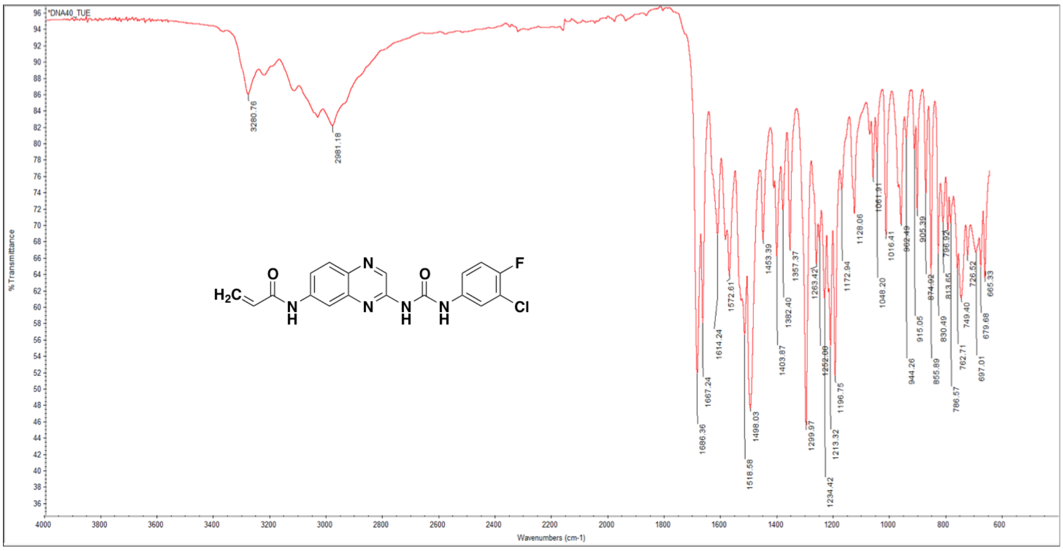

**Figure S90.** Infra red spectrum (ATR-FTIR) of compound *N*-(3-(3-(3-chloro-4-fluorophenyl) ureido)quinoxalin-6-yl) acrylamide **(7m)** (bands in cm$^{-}$).

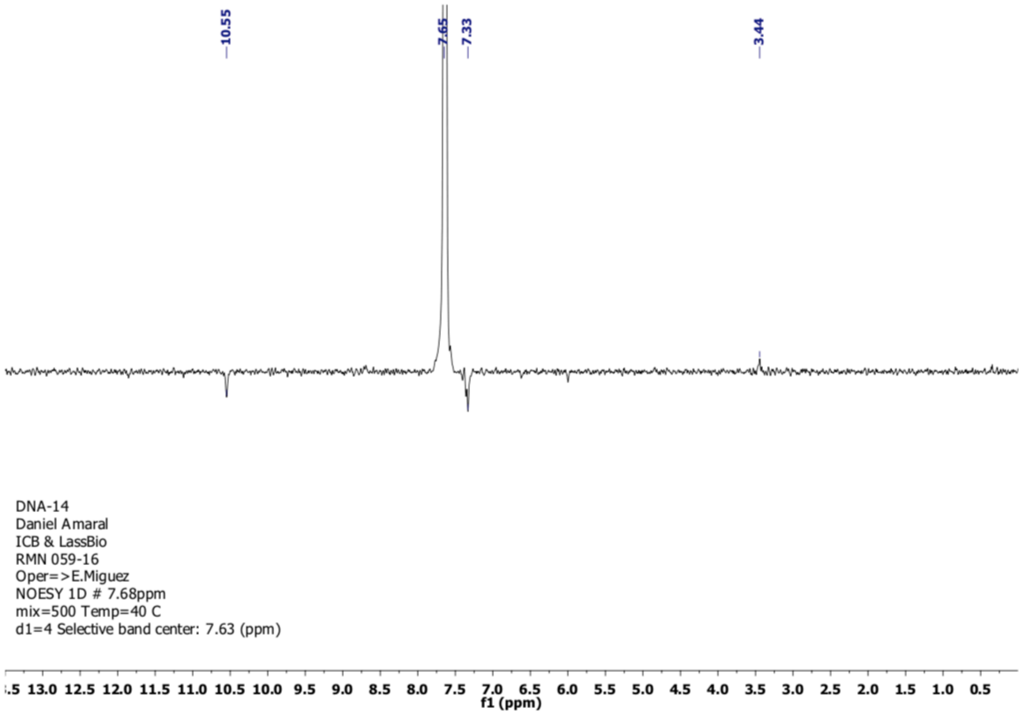


**Figure S91.** NOESY 1D spectrum of of compound 1-(7-nitroquinoxalin-2-yl)-3-phenylurea **(9c)** with irradiation at 7.68 ppm (DMSO-$d_6$/300MHz/TMS)

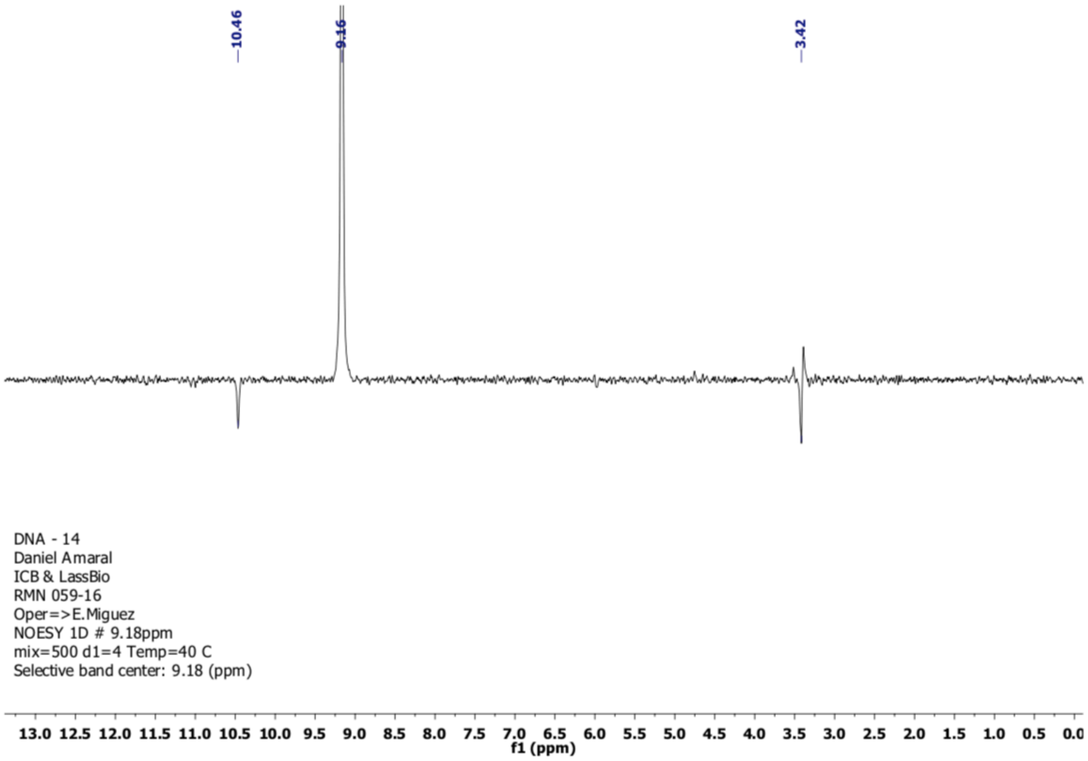


**Figure S92.** NOESY 1D spectrum of of compound 1-(7-nitroquinoxalin-2-yl)-3-phenylurea **(9c)** with irradiation at 9.18 ppm (DMSO-$d_6$/300MHz/TMS)

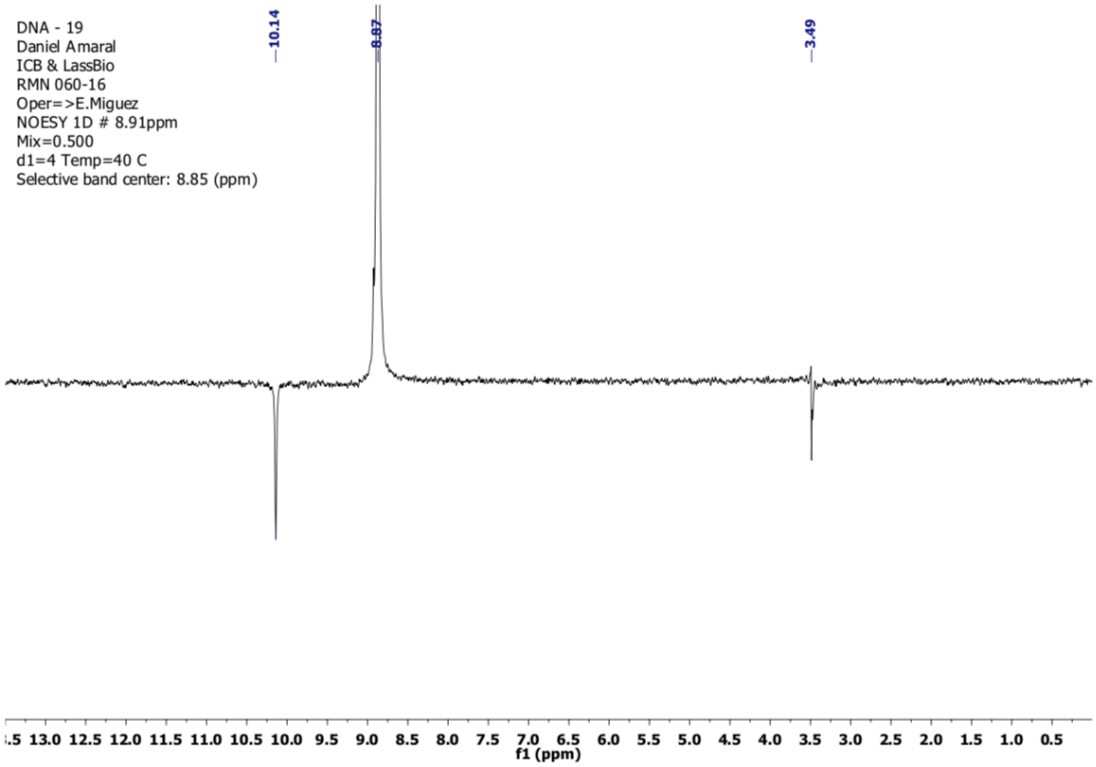


**Figure S93.** NOESY 1D spectrum of compound *N*-(3-(3-phenylureido)quinoxalin-6-yl) acrylamide **(7h)** with irradiation at 8.91 ppm (DMSO-$d_6$/300MHz/TMS)

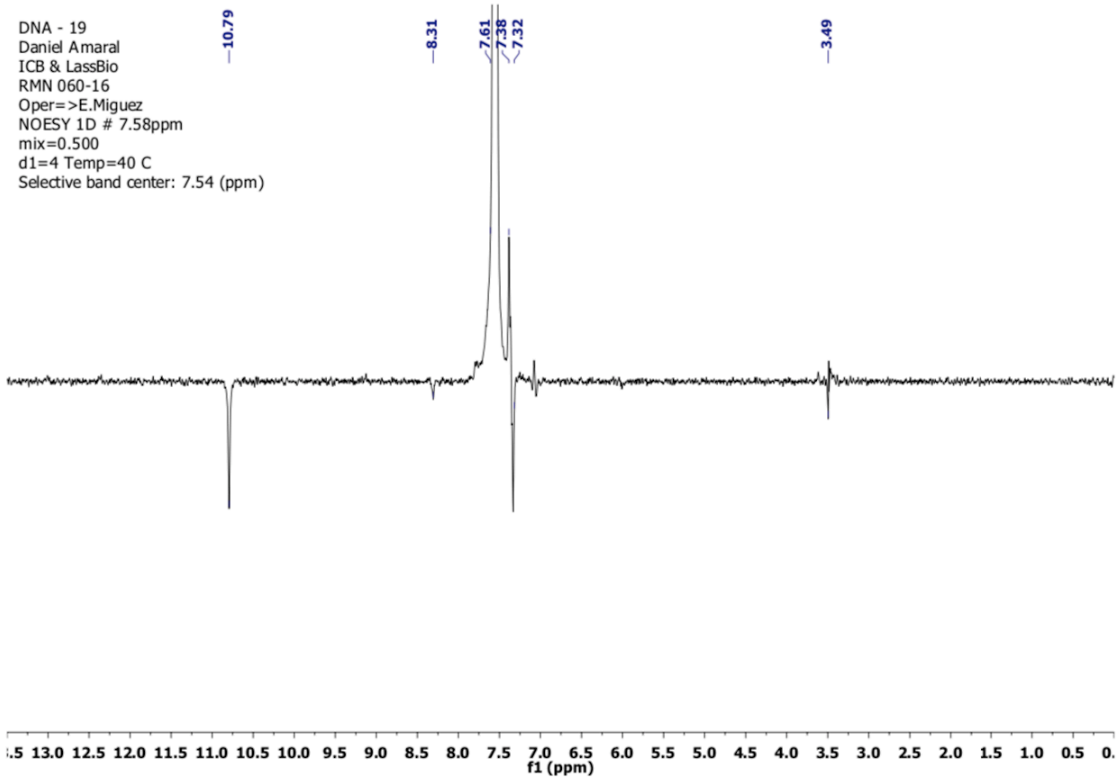


**Figure S94.** NOESY 1D spectrum of compound *N*-(3-(3-phenylureido)quinoxalin-6-yl) acrylamide **(7h)** with irradiation at 7.58 ppm (DMSO-$d_6$/300MHz/TMS)
